# Acupuncture-related therapies for opioid-induced constipation in patients with cancer: a systematic review and network meta-analysis

**DOI:** 10.3389/fmed.2026.1869529

**Published:** 2026-08-26

**Authors:** Xinkun Liu, Huaqiang Shao, Jiayue Yang, He Chen, Ruimin Jiao, Guangxian Yu, Jiaxiang Shi, Wenquan Su, E. Zhang, Yiqi Liang, Zhishun Liu, Weiming Wang

**Affiliations:** 1 Department of Acupuncture and Moxibustion, Guang'anmen Hospital, China Academy of Chinese Medical Sciences, Beijing, China; 2 Department of Specialty Examination, Affiliated Hospital of Shandong University of Traditional Chinese Medicine, Jinan, China; 3 School of Clinical Medicine, Chengdu University of Traditional Chinese Medicine, Chengdu, China; 4 Hospital of Chengdu University of Traditional Chinese Medicine, Chengdu, China; 5 Department of Traditional Chinese Medicine, Beijing Friendship Hospital, Capital Medical University, Beijing, China; 6 Department of Acupuncture, Datun Community Healthcare Service Center, Beijing, China; 7 Department of Acupuncture, Guangwai Hospital of Xicheng District, Beijing, China; 8 Traditional Chinese Medicine (Zhong Jing) School, Henan University of Chinese Medicine, Zhengzhou, China

**Keywords:** acupuncture – therapy, cancer-related pain, network meta-analysis, opioid-induced constipation, systematic review

## Abstract

**Objectives:**

To evaluate the efficacy and safety of acupuncture-related therapies for opioid-induced constipation (OIC) in patients with cancer through pairwise and network meta-analysis (NMA).

**Methods:**

We searched eight electronic databases from inception to June 28, 2025, and identified randomized controlled trials (RCTs) evaluating acupuncture-related therapies for OIC in cancer patients, with or without laxatives. Pairwise and Bayesian NMA were performed using R. Primary outcomes included constipation-related symptoms and stool form, assessed using the Cleveland Constipation Scale (CCS), weekly spontaneous bowel movements (weekly SBMs), Bowel Function Index (BFI), Patient Assessment of Constipation Symptoms (PAC-SYM), and Bristol Stool Form Scale (BSFS). Treatments were ranked using the surface under the cumulative ranking curve (SUCRA), and the certainty of evidence was evaluated using GRADE.

**Results:**

Thirty-six RCTs involving 3,351 participants and 15 acupuncture-related interventions were included. Direct evidence from pairwise meta-analyses and single-study comparisons suggested that some acupuncture-related therapies, alone or combined with osmotic laxatives (OSM), may improve CCS, weekly SBMs, BFI, PAC-SYM, and BSFS compared with OSM or other controls. In the NMA, AA+OSM, AP+OSM, and APA+OSM were associated with statistically significant reductions in BFI scores compared with OSM, although these estimates were supported by low-certainty evidence. No statistically significant differences were detected for CCS, weekly SBMs, PAC-SYM, BSFS, or PAC-QOL total score. Among studies reporting safety outcomes, adverse events were generally mild and infrequent, mainly gastrointestinal symptoms and local skin reactions. Most network estimates were based on sparse evidence and supported by low to very low certainty. SUCRA-based rankings were considered exploratory because several interventions were supported by only one trial and global inconsistency could not be formally assessed.

**Conclusion:**

Acupuncture-related therapies may improve selected constipation-related symptoms and aspects of constipation-related quality of life in patients with cancer-related OIC. Reported adverse events were generally mild. However, these findings should be considered exploratory because the available evidence was limited and generally of low certainty.

**Systematic review registration:**

The publicly accessible registration record is available at: https://www.crd.york.ac.uk/PROSPERO/view/CRD42024598002, PROSPERO CRD42024598002.

## 1 Introduction

Approximately 62% of patients with cancer worldwide suffer from opioid-induced constipation (OIC) (1). OIC is characterized by reduced stool frequency, increased straining, incomplete evacuation, and difficulty in defecation (1–3). Unlike other opioid-related side effects, OIC presents distinct clinical challenges, causing significant physiological, psychological, and social burdens (2–5). In cases of severe OIC, patients may feel compelled to voluntarily reduce or discontinue opioid therapy in an attempt to alleviate gastrointestinal symptoms. However, reducing or discontinuing opioid therapy may worsen pain control and further impair quality of life (6), particularly in patients with cancer who depend on long-term opioid treatment.

The clinical management of OIC typically involves a multimodal approach (7–9). While foundational lifestyle modifications remain essential, clinical guidelines usually recommend the use of standard laxatives and peripherally acting *μ*-opioid receptor antagonists (PAMORAs) for OIC management (8, 9). Standard laxatives are commonly prescribed as first-line therapies because of their easy availability (10). Some examples of these laxatives include osmotic laxatives (OSM; e.g., lactulose), stimulant laxatives (e.g., bisacodyl), lubricant or enema laxatives (e.g., glycerin suppositories), and surfactant laxatives (e.g., docusate sodium). However, these agents do not adequately address the underlying pathophysiological mechanisms of OIC and often provide insufficient symptom relief or are associated with adverse effects (11, 12). Although PAMORAs (e.g., naloxegol and naldemedine) are currently considered the best mechanism-based treatment for OIC, their widespread clinical use remains limited because of their high cost and a relatively higher incidence of adverse effects compared to those reported with standard laxatives (9, 10).

Acupuncture, a therapeutic technique based on the meridian and acupoint theory of traditional Chinese medicine (TCM), involves the insertion and manipulation of fine needles at specific body points to achieve therapeutic effects (13). Acupuncture has now evolved into a comprehensive treatment system encompassing multiple modalities, including needling techniques, herbal medicine, moxibustion (MOX), tuina (Chinese therapeutic massage), dietary adjustments, and lifestyle interventions, collectively forming the broader acupuncture and herbal medicine (AHM) system (14). This system includes a variety of acupuncture-related therapies, including classic acupuncture, electroacupuncture (EA), auricular acupuncture (AA), acupoint application (APA), acupressure (AP), press needle (PN), and MOX. It has been suggested in recent years that acupuncture for OIC offers a favorable safety profile, demonstrates a therapeutic potential, and is associated with fewer adverse events (15). Various systematic reviews and meta-analyses have evaluated the efficacy and safety of acupuncture-related therapies for OIC (15). However, previous reviews have mainly focused on pairwise comparisons and have not comprehensively compared the available acupuncture-related interventions. We therefore conducted an NMA to integrate direct and indirect evidence and compare their relative effects in patients with cancer-related OIC.

## 2 Methods

This systematic review was conducted in accordance with the Cochrane Handbook for Systematic Reviews of Interventions (16) and reported following the Preferred Reporting Items for Systematic Reviews and Meta-Analyses extension statement for NMA (PRISMA-NMA) (17). The study protocol was prospectively registered in PROSPERO (CRD42024598002).

### 2.1 Search strategy

A systematic literature search was conducted by a medical information specialist across eight electronic databases (four English databases: PubMed, Embase, Web of Science, and Cochrane Library, and four Chinese databases: Wanfang, China National Knowledge Infrastructure (CNKI), Chinese Biomedical Literature Database (CBM), and Chinese Scientific Journals Database (VIP)), from their inception to 28 June 2025. A rigorous search strategy was developed based on the PICOS (P: Population, I: Intervention, C: Comparison, O: Outcome, S: Study design) framework and incorporated a combination of medical subject heading terms, keywords, and free-text terms. The search terms focused on the following themes: (1) “opioid-induced constipation” or “constipation” combined with (2) “opioids” combined with (3) “acupuncture” or “electroacupuncture” or “moxibustion” or “acupressure” or “transcutaneous electric nerve stimulation” or “acupoint application” combined with (4) “randomized controlled trial.” The search strategies used herein are detailed in Supplementary material 1.

### 2.2 Inclusion criteria

Our participants, intervention, comparator, outcomes and study design (PICOS) were selected based on the following criteria:

1. Participants: Patients with cancer (≥18 years old) who experienced OIC and met the Rome III or IV criteria (18, 19), the Multidisciplinary Working Group Consensus Statement (20), or other constipation diagnostic criteria issued by Chinese authorities (21, 22). There were no restrictions on sex, geographic location, ethnicity, or cancer stage.
2. Interventions: Participants in the intervention group received acupuncture-related therapies, which, according to the AHM system (14), involved stimulation on the body surface and/or deep tissues. These included EA, AA, APA, AP, PN, MOX, manual acupuncture (MA), acupoint catgut embedding (ACE), and transcutaneous electrical acupoint stimulation (TEAS), with or without the combination of the same medication as the control group. We adopted a pragmatic inclusion strategy and prespecified that variability in acupuncture protocols (e.g., technique, acupoint selection, frequency, number of sessions, and session duration) would be documented in detail.
3. Comparisons of interest included the following: (1) Acupuncture versus blank (no-treatment) control (BC); (2) acupuncture versus sham/placebo acupuncture (SA); (3) acupuncture versus conventional treatment; (4) acupuncture combined with conventional treatment versus either sham/placebo acupuncture combined with the same conventional treatment or the same conventional treatment alone. Conventional treatment included guideline-recommended first-line laxative-based therapy (8, 9), or a combination of SA and laxatives. Laxatives were grouped according to their predominant pharmacological mechanisms into osmotic laxatives (OSM; lactulose and polyethylene glycol) and stimulant laxatives (STI; bisacodyl and phenolphthalein), which were retained as separate network nodes. Specific agents, doses, frequencies, and rescue treatments were considered in the transitivity assessment.
4. Outcomes: The study outcomes were informed by the patient-reported outcome measures (PROMs) for constipation (23). The primary outcomes included at least one symptom-related measure for clinical trials in constipation from among the following: bowel movement category (e.g., the Cleveland Constipation Scale (CCS), spontaneous bowel movements per week (weekly SBMs), the Bowel Function Index (BFI) total score, and the Patient Assessment of Constipation–Symptoms (PAC-SYM)) and stool category (e.g., the Bristol Stool Form Scale (BSFS)). The secondary outcomes included the BFI subscales, the Patient Assessment of Constipation Quality of Life questionnaire (PAC-QOL) total score and its subscales, and adverse events. Total scores were prioritized over subscale results in the main interpretation.
5. Study design: Published randomized controlled trials (RCTs) with two or more study groups, either in Chinese or English.

### 2.3 Exclusion criteria

1. Animal studies, literature reviews, observational studies, empirical summaries, conference abstracts, case reports, or study protocols.
2. Duplicate RCT publications or RCTs with incomplete data.
3. Clinical trials lacking randomization to mitigate substantial bias.
4. Studies with obvious confounding factors (e.g., concomitant non-standardized treatments or imbalanced co-interventions between groups).

### 2.4 Study selection

After removing duplicate records, two independent reviewers screened the identified studies in three stages: (i) title screening, (ii) abstract screening, and (iii) full-text review, according to the predefined eligibility criteria. The full texts of all potentially relevant studies were subsequently retrieved for further assessment. Any disagreements were resolved through discussion and consensus. If no consensus could be reached, a third reviewer acted as an arbiter.

### 2.5 Data extraction

All eligible studies were independently subjected to data extraction by two reviewers, and the extracted data were subsequently cross-checked by a third reviewer. The data extraction form captured a range of study characteristics, including the first author’s name, publication year, country or region, patient demographic (e.g., age and gender), sample size, details of interventions in both the treatment and control groups, treatment duration, primary outcome measures, and adverse events. Any discrepancies identified during data extraction were resolved by re-examining the original studies and through discussions with a third reviewer until a consensus was reached.

### 2.6 Risk of bias assessment

The methodological quality and risk of bias of the included studies were independently evaluated by two reviewers using the revised Cochrane Risk of Bias tool (RoB 2) (24). Assessments were conducted for each relevant study result rather than assigning a single judgment to the entire study. This tool assesses five key domains: (1) randomization process; (2) deviations from the intended interventions; (3) missing outcome data; (4) outcome measurement; and (5) selection of reported results. For deviations from the intended interventions, judgments were based on whether awareness of treatment allocation resulted in important deviations from the planned intervention. For patient-reported outcomes, the potential influence of unblinded participants on outcome reporting was considered in the outcome-measurement domain. The absence of reported missing data was not assumed to indicate complete outcome data; instead, the numbers randomized and analyzed, attrition, and reasons for missingness were considered. Each domain was categorized as having a “low risk,” “some concerns,” or “high risk” of bias. Any discrepancies between reviewers were resolved through discussion or, if necessary, adjudication by a third reviewer.

### 2.7 Statistical analysis

Pairwise meta-analysis was performed within a frequentist framework using the “meta” package in R (V. 4.5.1), initially comparing studies with similar interventions and outcome measures. Quantitative synthesis was performed only when at least two randomized controlled trials evaluated the same intervention–outcome comparison; otherwise, results were summarized descriptively. Risk ratios (RRs) with 95% confidence intervals (CIs) were used for dichotomous outcomes, and mean differences (MDs) with 95% CIs were used for continuous outcomes measured using the same instrument or scale. Outcome directions were harmonized, with lower scores indicating improvement for CCS, BFI, PAC-SYM, and PAC-QOL, and higher scores indicating improvement for weekly SBMs and BSFS. A BSFS comparison containing a study arm with a reported SD of zero was excluded from the primary quantitative analysis. In a sensitivity analysis, the zero SD was replaced with the pooled within-group SD derived from the non-zero BSFS arms of the other included studies, in accordance with established guidance for imputing unavailable SDs (16), and the pairwise meta-analysis and NMA were repeated. Random-effects models were used when *I*^2^ ≥ 50% or *p* < 0.10; otherwise, fixed-effect models were applied. Statistical significance was defined as *p* < 0.05.

Transitivity was assessed narratively across direct treatment comparisons using clinically relevant potential effect modifiers related to patient characteristics, opioid exposure, co-interventions, and acupuncture protocols (16). Comparisons were categorized as having “No concerns,” “Some concerns,” or “Major concerns” based on the completeness of reporting and the comparability of potential effect modifiers across comparisons. Single-study comparisons were not automatically categorized as having major concerns. Because most comparisons were supported by only a few studies, subgroup analyses, meta-regression, and sensitivity analyses based on these effect modifiers were not feasible.

The Bayesian NMA was performed using the “multinma” and “BUGSnet” packages in R with Markov chain Monte Carlo methods. The “multinma” package was used for primary effect estimation and treatment ranking, whereas “BUGSnet” was used for model diagnostics and graphical presentation. RRs were used for dichotomous outcomes and MDs for continuous outcomes, with 95% credible intervals (CrIs) reported for all network estimates. Priors implemented in “multinma” were used: normal distributions of N(0, 100^2^) and N(0, 10^2^) for baseline and relative treatment effects, respectively, and a half-normal distribution with a scale of 5 for the heterogeneity standard deviation. Four chains were run for 2,000 iterations each, including 1,000 warm-up iterations, with thinning set to 1. In BUGSnet, three chains were run with 1,000 adaptation iterations, 1,000 burn-in iterations, and 10,000 sampling iterations, using the package-default vague priors. Convergence was assessed using R̂/PSRF and effective sample size, with R̂/PSRF values close to 1 and adequate effective sample sizes indicating satisfactory convergence, and model fit was evaluated using DIC, residual deviance, and leverage plots. The BFI network was analyzed using a fixed-effect model because of its clinical and methodological homogeneity and negligible estimated heterogeneity, whereas random-effects models were used for the remaining outcomes to account for potential heterogeneity. Treatment rankings were summarized using SUCRA and interpreted together with effect estimates and uncertainty. Global inconsistency could not be formally assessed because the networks lacked closed loops. Comparison-adjusted funnel plots were generated for outcome networks including at least 10 studies to explore small-study effects. Egger’s test was planned for individual pairwise comparisons including at least 10 studies.

### 2.8 Certainty of evidence

The certainty of evidence was evaluated using the Grading of Recommendations Assessment, Development and Evaluation (GRADE) approach adapted for NMA (25, 26). Because all included studies were RCTs, the initial level of evidence for each outcome was rated as “high.” Direct-comparison evidence was assessed for risk of bias, inconsistency, indirectness, imprecision, and publication bias. For indirect comparisons, the lower certainty of the two direct comparisons contributing to the dominant first-order indirect pathway was used as the starting point, with further consideration of intransitivity. For network estimates, the higher certainty of the direct and indirect evidence was used as the starting point, followed by consideration of incoherence and imprecision. Certainty was categorized as high, moderate, low, or very low.

## 3 Results

### 3.1 Study search and description

**Figure 1:**
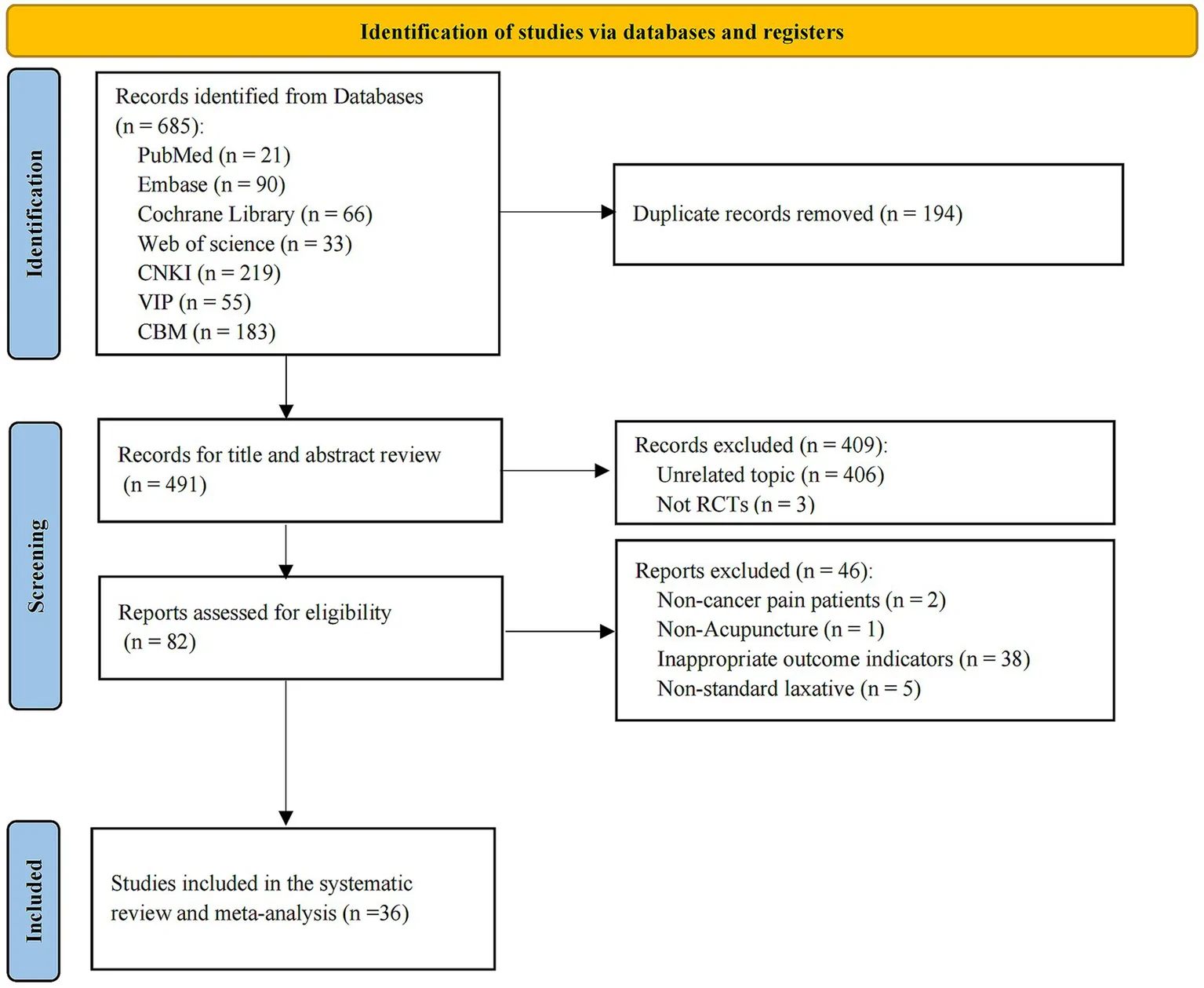
PRISMA flow diagram of the study selection process. CNKI, China National Knowledge Infrastructure; VIP, Chinese Scientific Journals Database; CBM, Chinese Biomedical Literature Database.

In total, 685 related studies were retrieved from the databases. Among these, 194 duplicate studies were removed. We also deleted 455 studies after reading their titles, abstracts, and full text. Thus, 36 studies (27–62), with a total of 3,351 participants, were included in the final analysis (Figure 1; Supplementary material 2).

The included 36 studies involved 15 distinct acupuncture-related interventions, comprising seven acupuncture-related monotherapies and eight combination therapies. APA was the most frequently used monotherapy and was employed in 14 of the 36 studies (28, 30, 32–35, 37, 41, 43, 45, 46, 49, 52, 54), followed by MA [one study (44)], EA [three studies (29, 31, 60)], AP [two studies (47, 61)], MOX [two studies (36, 56)], TEAS [one study (62)], and PN [one study (38)]. Twelve studies adopted combination therapies: APA+OSM [four studies (39, 40, 51, 57)], MA+OSM [one study (58)], AP+OSM [one study (27)], AA+OSM [two studies (42, 50)], TEAS+OSM [one study (59)], MOX+OSM [one study (48)], ACE+OSM [one study (55)], and PN+OSM [one study (53)] (Tables 1, 2).

**Table 1:** Study and participant characteristics of the included RCTs.

Study	Country	Diagnostic criteria	Sample size		Gender (male/female)		Age, years (Mean ± sd)		Intervention		Blank control method	Outcomes	Safety
			T	C	T	C	T	C	T	C			
Wang et al. (60)	China	Rome IV	50	50	29/21	27/23	63.6 ± 10.40	65.1 ± 10.6	EA	SA	—	② ④	R
Yildirim et al. (61)	Turkey	NR	100	100	50/20	38/32	60.93 ± 14.05	60.22 ± 12.81	AP	BC	Maintained usual diet, hydration, and use of stool softeners/laxatives	⑩ ⑪ ⑫ ⑬	R
Cai et al. (59)	China	MWGCS	130	138	74/56	72/66	55.3 ± 3.10	51.2 ± 3.40	TEAS+OSM	SA+OSM	—	⑥ ⑦ ⑧	R
Zhu et al. (62)	China	Rome IV	98	100	52/46	45/55	57.12 ± 9.93	59.54 ± 10.77	TEAS	OSM	—	① ⑨	R
Chen et al. (27)	China	Other	50	50	38/12	37/13	60.2 ± 11.00	61.3 ± 11.40	AP+OSM	OSM	—	⑤	NR
Deng (28)	China	Rome IV	45	45	25/20	26/19	57.84 ± 11.57	62.44 ± 12.62	APA	OSM	—	④ ⑨	R
Du et al. (29)	China	Rome IV	50	50	27/23	25/25	63.44 ± 13.35	62.12 ± 13.058	EA	OSM	—	①	R
He et al. (30)	China	Rome IV	60	60	31/23	33/19	61.43 ± 15.49	59.75 ± 15.81	APA	OSM	—	③	NR
Li (31)	China	Rome IV	22	22	11/11	9/11	68.86 ± 8.73	67.00 ± 10.33	EA	OSM	—	② ④	R
Li (32)	China	Other	45	45	25/20	24/21	54.23 ± 2.20	55.41 ± 2.01	APA	BC	Diet guidance, abdominal massage, emotional support, health education, and 30–60 min of moderate aerobic exercise daily.	②	NR
Liang et al. (33)	China	Rome IV	30	30	18/12	16/14	48 ~ 76	52 ~ 79	APA	OSM	—	② ⑥ ⑦ ⑧	R
Lin (34)	China	Rome IV	39	39	17/20	19/17	61.14 ± 11.36	58.36 ± 12.68	APA	OSM	—	① ② ⑨	R
Lin et al. (35)	China	Rome IV	22	22	12/10	13/9	43.33 ± 12.25	44.12 ± 11.96	APA	STI	—	①	R
Liu (36)	China	Rome IV	35	35	26/7	25/8	65.73 ± 7.072	66.67 ± 7.279	MOX	OSM	—	②	R
Ma and Zhao (37)	China	Other	45	45	21/24	26/19	46.16 ± 5.73	56.47 ± 4.95	APA	BC	Diet guidance, abdominal massage	③ ⑥ ⑦ ⑧ ⑩ ⑪ ⑫ ⑬	NR
Ma et al. (38)	China	Rome IV	36	36	20/16	19/17	52.2 ± 10.10	51.8 ± 10.30	PN	OSM	—	① ⑨	NR
Mao et al. (39)	China	Rome IV	29	29	21/8	17/12	56.9 ± 12.13	56.2 ± 10.9	APA+OSM	OSM	—	①	R
Ouyang et al. (40)	China	MWGCS	68	61	33/35	37/24	66.96 ± 7.93	64.32 ± 9.33	APA+OSM	OSM	—	① ② ⑨	NR
Qi (41)	China	Rome IV	35	35	19/14	15/17	63.33 ± 11.40	63.34 ± 9.20	APA	OSM	—	① ②	R
Qu and Ye (42)	China	Other	50	50	59/41		60.00 ± 9.50		AA+OSM	OSM	—	①	NR
Tang and Wang (43)	China	Other	37	37	22/15	15/22	47.58 ± 2.54	47.53 ± 2.51	APA	OSM	—	⑨	NR
Tian (44)	China	Rome IV	35	35	20/13	18/14	58.73 ± 7.74	60.16 ± 8.80	MA	OSM	—	① ⑨	R
Wang and Zhang (45)	China	Other	76	76	42/34	40/36	61.45 ± 5.32	60.45 ± 7.12	APA	OSM	—	④	R
Xia et al. (46)	China	Other	35	35	18/17	20/15	50.33 ± 5.13	50.24 ± 5.08	APA	BC	Diet guidance, abdominal massage, emotional support, and health education	③ ⑩ ⑪ ⑫ ⑬	NR
Xu (47)	China	Other	44	44	28/16	26/18	59.41 ± 9.38	60.81 ± 8.98	AP	BC	Diet guidance	②	NR
Xu et al. (48)	China	Rome IV	42	40	25/17	22/18	35.00 ~ 70.00	34.00 ~ 68.00	MOX+OSM	OSM	—	② ④ ⑨	NR
Xu and Lu (49)	China	Rome IV	43	43	46/40		51.36 ± 3.57		APA	OSM	—	①	NR
Yang et al. (50)	China	Rome IV	30	30	NR	NR	NR	NR	AA+OSM	OSM	—	② ⑤ ⑥ ⑦ ⑧ ⑨	R
Yang et al. (51)	China	Other	31	31	15/16	14/17	66.1 ± 8.5.	65.6 ± 8.3.	APA+OSM	OSM	—	⑤	NR
Yin et al. (52)	China	Rome IV	38	38	NR	NR	NR	NR	APA	OSM	—	①	R
Zhang et al. (53)	China	Rome IV	50	50	28/22	29/21	58.82 ± 9.64	58.43 ± 9.71	PN+OSM	OSM	—	① ② ⑨	NR
Zhao et al. (54)	China	Other	40	40	25/15	21/15	55.72 ± 8.01	55.18 ± 8.64	APA	SA	—	⑥ ⑦ ⑧ ⑩ ⑪ ⑫ ⑬	NR
Zheng (55)	China	Rome IV	35	35	17/16	17/16	52.27 ± 8.43	52.49 ± 10.71	ACE+OSM	OSM	—	②	R
Zhou (56)	China	Rome IV	35	35	22/13	21/14	57.26 ± 11.76	58.51 ± 12.29	MOX	STI	—	①	NR
Zhu et al. (57)	China	Other	35	35	19/16	21/14	57.5 ± 10.10	56.2 ± 9.20	APA+OSM	OSM	—	②	NR
Zhuo et al. (58)	China	Rome IV	30	30	13/17	14/16	62.87 ± 7.66	63.77 ± 8.32	MA+OSM	OSM	—	①	NR

R, reported; NR, not reported; Other, diagnostic criteria of constipation by Chinese official authorities.

MWGCS, Multidisciplinary Working Group Consensus Statement; OSM, osmotic laxatives; STI, stimulant laxatives; MA, manual acupuncture; EA, electroacupuncture; AA, auricular acupuncture; ACE, acupoint catgut embedding; TEAS, transcutaneous electrical acupoint stimulation; APA, acupoint application; AP, acupressure; MOX, moxibustion; PN, press needle; BC, blank control; SA, sham acupuncture. ① Cleveland Constipation Scales (CCS); ② Bristol Stool Form Scale (BSFS); ③ Patient Assessment of Constipation–Symptoms (PAC-SYM); ④ spontaneous bowel movements per week (weekly SBMs); ⑤ Bowel Function Index (BFI); ⑥ BFI: ease of defecation; ⑦ BFI: feeling of incomplete bowel evacuation; ⑧ BFI: personal judgment of constipation; ⑨ Patient Assessment of Constipation Quality of Life questionnaire (PAC-QOL); ⑩ PAC-QOL: physical discomfort; ⑪ PAC-QOL: psychosocial discomfort; ⑫ PAC-QOL: worries and concerns; ⑬ PAC-QOL: satisfaction.

**Table 2:** Characteristics of acupuncture-related interventions in the included RCTs.

Study	Acupoints	Treatment duration	Retaining time	Dose or frequency
Wang et al. (60)	Tianshu (ST25), Fujie (SP14), Shangjuxu (ST37)	8 weeks	30 min	3/week
Yildirim et al. (61)	Tianshu (ST25), Zhongwan (CV12), Guanyuan (CV4)	4 weeks	8 min	1/day
Cai et al. (59)	Main acupoint: Tianshu (ST25), Guanyuan (CV4), Qihai (CV6), Zusanli (ST36), Shangjuxu (ST37), Additional points: Ganshu (BL18), Shenshu (BL23), Sanyinjiao (SP6)	2 weeks	30 min	1/day
Zhu et al. (62)	Tianshu (ST25), Zhongwan (CV12)	2 weeks	30 min	1/day
Chen et al. (27)	Shenque (CV8), Zhongwan (CV12), Tianshu (ST25)	2 weeks	NR	1/day
Deng (28)	Shenque (CV8)	2 weeks	48 h	every 2 days
Du et al. (29)	Main acupoint: Tianshu (ST25) Additional points (based on syndrome differentiation): Qihai (CV6), Zusanli (ST36), Guanyuan (CV4), Taixi (KI3), Hegu (LI4), Sanyinjiao (SP6)	2 weeks	30 min	5/week
He et al. (30)	Shenque (CV8)	4 weeks	4 h	1/day
Li (31)	Tianshu (ST25), Shangjuxu (ST37), Zhigou (TE6), Zusanli (ST36)	2 weeks	20 min	1/day
Li (32)	Yongquan (KI1)	2 months	6 h	1/day
Liang et al. (33)	Shenque (CV8)	1 week	24 h	1/day
Lin (34)	Shenque (CV8), Zhongwan (CV12), Guanyuan (CV4)	2 weeks	4 h	1/day
Lin et al. (35)	Tianshu (ST25), Zusanli (ST36), Guanyuan (CV4), Sanyinjiao (SP6), Zhigou (TE6), Neiting (ST44), Qihai (CV6)	2 weeks	4–6 h	1/day
Liu (36)	Qihai (CV6), Guanyuan (CV4), Zhongwan (CV12), Shenque (CV8)	2 weeks	45 min	1/day
Ma and Zhao (37)	Shenque (CV8)	NR	NR	1/day
Ma et al. (38)	Zusanli (ST36), Hegu (LI4), Taichong (LR3), Zhigou (TE6), Shangjuxu (ST37)	3 weeks	1 min per session, once every 2–3 h	every 3 days
Mao et al. (39)	Shenque (CV8)	8 days	12 h	1/day
Ouyang et al. (40)	Shenque (CV8)	3 weeks	48 h	every 2 days
Qi (41)	Main acupoint: Tianshu (ST25), Guilai (ST29), Dachangshu (BL25), Zhigou (SJ6), Shangjuxu (ST37). Additional points (based on syndrome differentiation): Geshu (BL17), Zusanli (ST36), Neiting (ST44), Hegu (LI4), Qihai (CV6), Pishu (BL20), Guanyuan (CV4), Shenque (CV8), Sanyinjiao (SP6), Yinlingquan (SP9).	1 week	6 h	1/day
Qu and Ye (42)	Triple Burner (TF4), Spleen (CO13), Stomach (CO4), Liver (CO12), Lung (CO14), Large Intestine (CO7)	NR	Continuous pressure for 20 s, 4–6 presses per point, 5–6 times/d	Opposite side, every 3 days
Tang and Wang (43)	Shenque (CV8)	1 week	6 h	1/day
Tian (44)	Tianshu (ST25), Shangjuxu (ST37), Zusanli (ST36), Hegu (LI4), Dachangshu (BL25), Pishu (BL20), Zhigou (TE6)	2 weeks	30 min	5/week
Wang and Zhang (45)	Shenque (CV8)	2 weeks	4–6 h	1/day
Xia et al. (46)	Shenque (CV8)	1 week	6 h	1/day
Xu (47)	Zhigou (TE6), Hegu (LI4), Tianshu (ST25), Daheng (SP15), Zhongwan (CV12), Zusanli (ST36), Sanyinjiao (SP6)	2 weeks	1–2 s per stroke per acupoint, 50 strokes per session	2/day
Xu et al. (48)	Heat-sensitive acupoints	3 weeks	30–60 min	5/week
Xu and Lu (49)	Shenque (CV8)	1 week	6 h	1/day
Yang et al. (50)	Main auricular points: Subcortex (AT4), Brainstem, Abdomen (TF2), Large Intestine (CO7) Additional auricular points: Liver (CO12), Spleen (CO13), Triple Burner (TF4), Lung (CO14), Kidney (CO10)	2 weeks	Pressed 4 times/d (morning, noon, evening, bedtime), 2 min per acupoint	1/day
Yang et al. (51)	Shenque (CV8)	1 week	8 h	1/day
Yin et al. (52)	Shenque (CV8)	1 week	12 h	1/day
Zhang et al. (53)	Tianshu (ST25), Dachangshu (BL25), Shangjuxu (ST37), Zhigou (TE6), Zusanli (ST36)	2 weeks	24 h	1/day
Zhao et al. (54)	Shenque (CV8)	10 days	24 h	1/day
Zheng (55)	Tianshu (ST25), Dachangshu (BL25), Zusanli (ST36)	1 week	NR	1/week
Zhou (56)	Tianshu (ST25), Zhongwan (CV12), Shenque (CV8), Shangjuxu (ST37), Zusanli (ST36), (Bilateral) Zhigou (TE6), Guanyuan (CV4)	10 days	2–30 min per acupoint	1/day
Zhu et al. (57)	Shenque (CV8)	10 days	8 h	1/day
Zhuo et al. (58)	Baihui (GV20), Guanyuan (CV4), Qihai (CV6), Tianshu (ST25), Shangjuxu (ST37), Zusanli (ST36), Hegu (LI4), Taichong (LR3), Zhigou (TE6)	8 weeks	30 min	5/week

### 3.2 Risk of bias assessment

**Figure 2:**
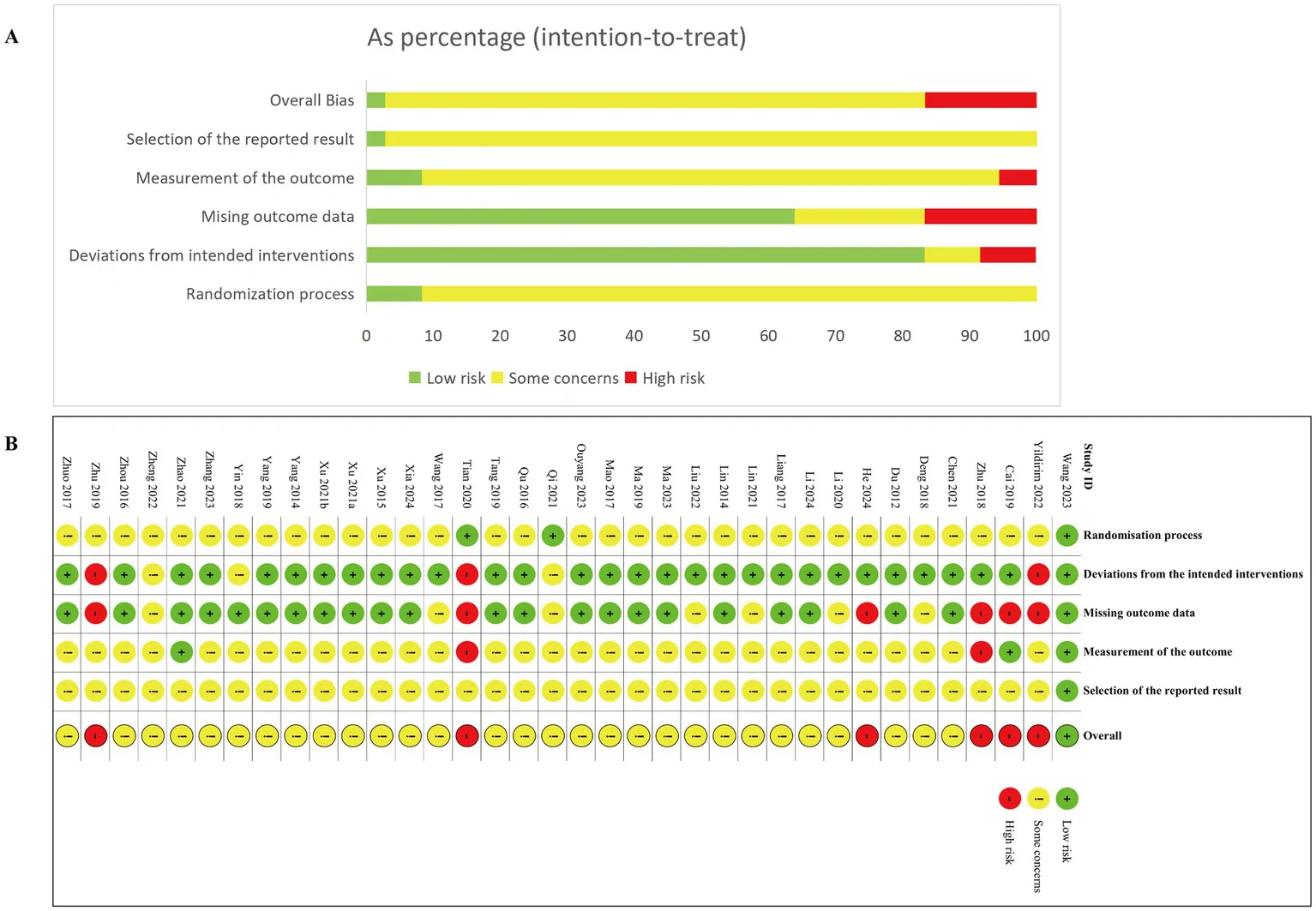
Risk of bias. **(A)** Risk of bias graph; **(B)** risk of bias summary.

Overall, 1 study (60) was judged as having a low risk of bias, 29 studies (27–29, 31–43, 45–56, 58) as having some concerns, and 6 studies (30, 44, 57, 59, 61, 62) as having a high risk of bias. Lack of participant blinding was not automatically considered a deviation from the intended intervention; however, its potential influence on patient-reported outcomes was considered in the outcome-measurement domain. For missing outcome data, 23 studies (27, 29, 32, 33, 35, 37–40, 42, 43, 46–54, 56, 58, 60) were rated as low risk, 7 studies (28, 31, 34, 36, 41, 45, 55) as having some concerns, and 6 studies (30, 44, 57, 59, 61, 62) as high risk. For outcome measurement, 3 studies (54, 59, 60) were rated as low risk, 31 studies (27–43, 45–53, 55–58, 61) as having some concerns, and 2 studies (44, 62) as high risk. Summary results are shown in Figure 2, and detailed study-, outcome-, and domain-specific judgments and supporting reasons are provided in Supplementary material 9.

### 3.3 Assessment of transitivity

Potential effect modifiers across the 20 direct treatment comparisons are summarized in Supplementary material 10. Within individual trials, the reported baseline characteristics were generally comparable between the treatment and control groups. However, comparability across different direct comparisons was limited by incomplete reporting and clinical and methodological variation. In particular, cancer stage and opioid type or dose were frequently not reported. Baseline constipation severity was assessed using different instruments and was unavailable in several comparisons. Variation was also observed in laxative regimens, rescue treatments, other concomitant care, acupoint selection, treatment frequency, retention or session time, and total treatment duration. Of the 20 direct comparisons, 9 were rated as having some concerns and 11 as having major concerns regarding transitivity. No comparison was rated as having no concerns. Therefore, although no clear systematic imbalance was consistently identified within individual trials, the transitivity assumption could not be confirmed with high confidence across the network. Because most comparisons were supported by few studies, formal subgroup analyses, meta-regression, and sensitivity analyses based on these effect modifiers were not feasible.

### 3.4 Effect estimates

Thirteen outcomes were included in the NMA, with varying numbers of interventions and network configurations. Model convergence was confirmed by PSRF values close to 1, indicating satisfactory convergence. However, global inconsistency could not be assessed because of the absence of closed loops. Across outcomes, the evidence networks were generally sparse, and several interventions were informed by only one trial. Therefore, NMA estimates and SUCRA-based rankings were interpreted as exploratory and were considered together with effect estimates, 95% CrIs, the number of contributing studies, and GRADE certainty.

#### 3.4.1 Primary outcomes

##### 3.4.1.1 CCS

**Figure 3:**
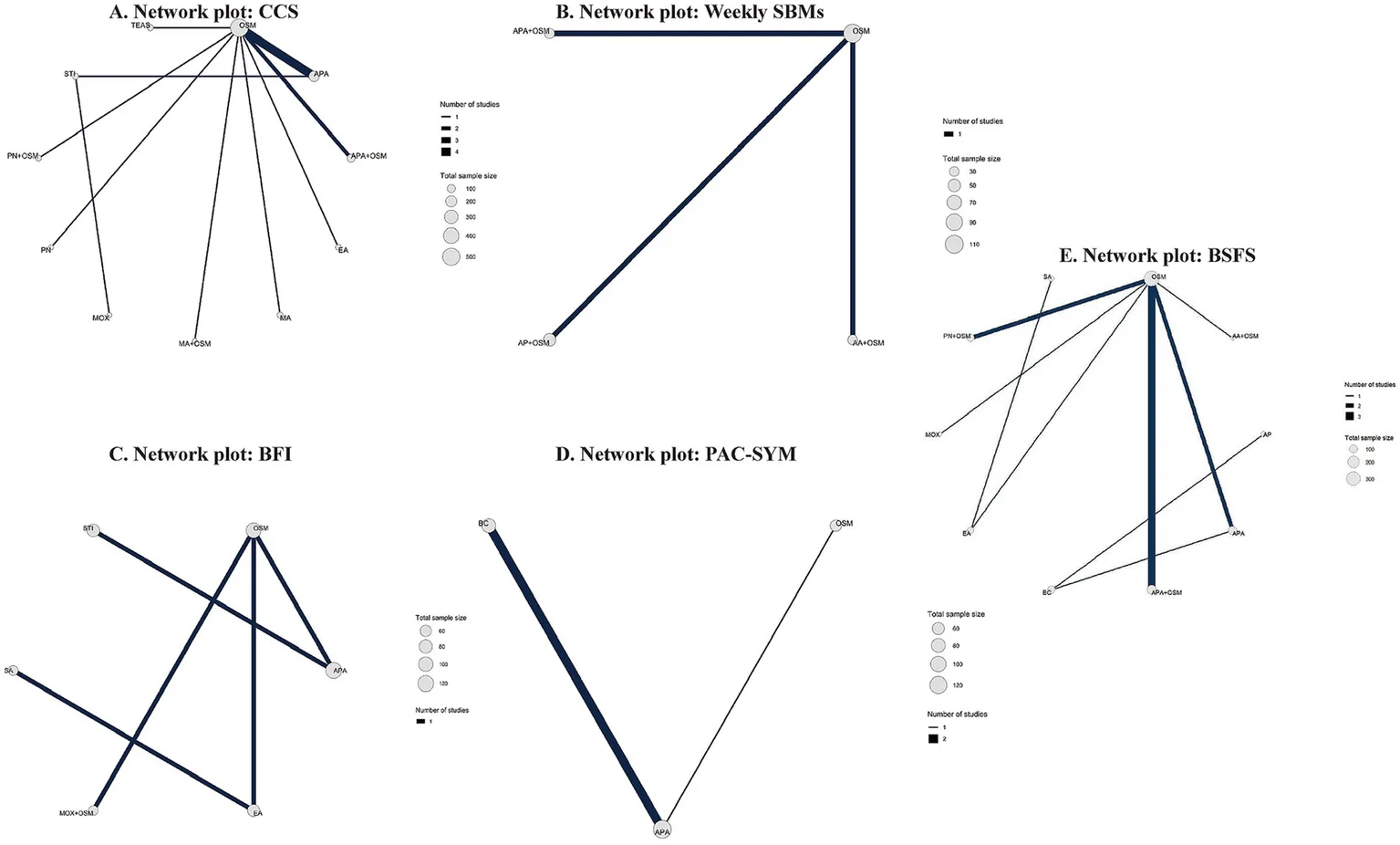
Network plot **(A–E)**. AA, auricular acupuncture; AP, acupressure; APA, acupoint application; BC, blank control; BFI, Bowel Function Index; BSFS, Bristol Stool Form Scale; CCS, Cleveland Constipation Scale; EA, electroacupuncture; MA, manual acupuncture; MOX, moxibustion; OSM, osmotic laxatives; PAC-SYM, Patient Assessment of Constipation-Symptoms; PN, press needle; SA, sham acupuncture; SBMs, spontaneous bowel movements per week; STI, stimulant laxatives; TEAS, transcutaneous electrical acupoint stimulation.

**Figure 4:**
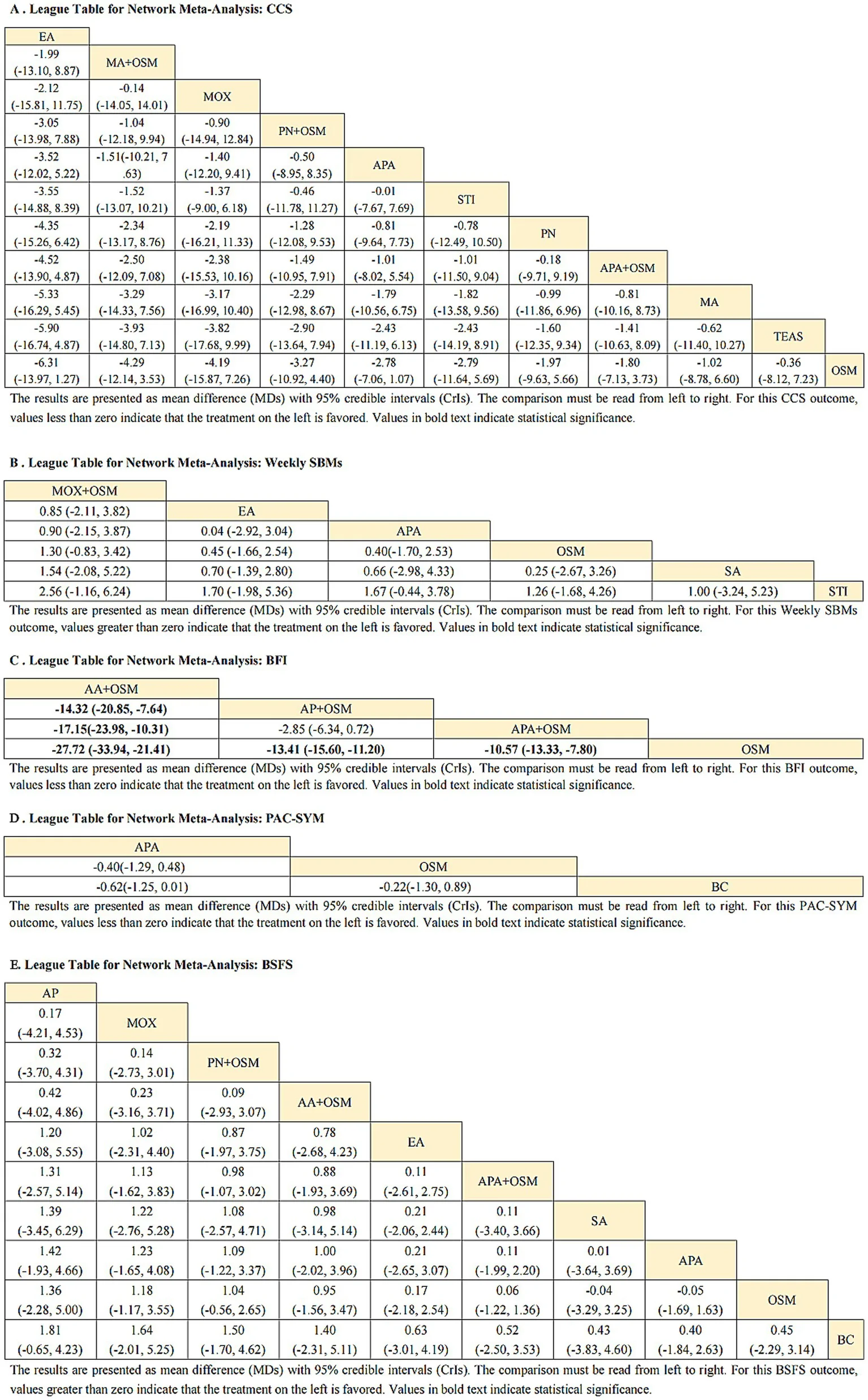
League table for network meta-analysis **(A–E)**. AA, auricular acupuncture; AP, acupressure; APA, acupoint application; BC, blank control; BFI, Bowel Function Index; BSFS, Bristol Stool Form Scale; CCS, Cleveland Constipation Scale; EA, electroacupuncture; MA, manual acupuncture; MOX, moxibustion; OSM, osmotic laxatives; PAC-SYM, Patient Assessment of Constipation-Symptoms; PN, press needle; SA, sham acupuncture; SBMs, spontaneous bowel movements per week; STI, stimulant laxatives; TEAS, transcutaneous electrical acupoint stimulation.

**Figure 5:**
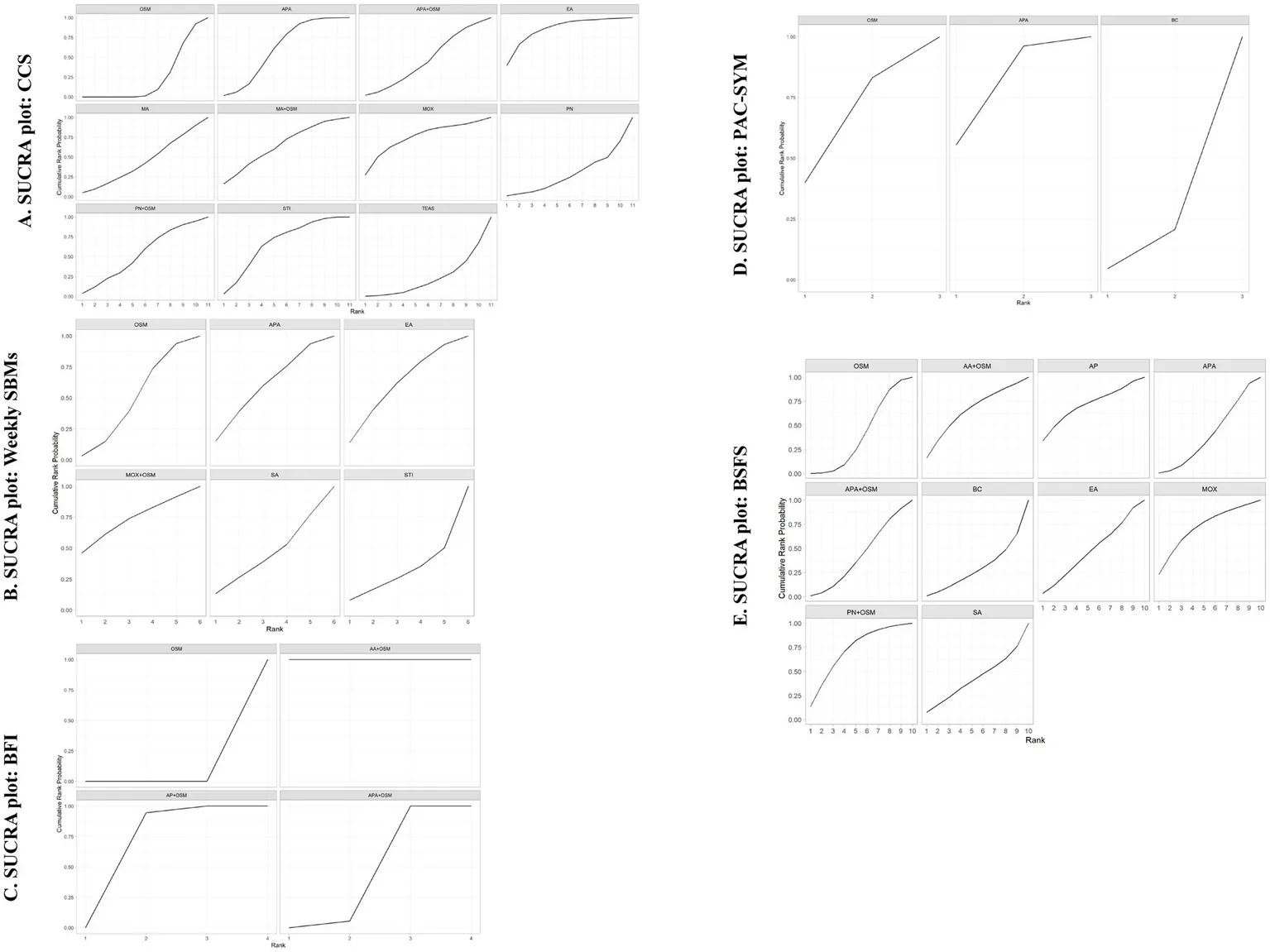
SUCRA plot **(A–E)**. AA, auricular acupuncture; AP, acupressure; APA, acupoint application; BC, blank control; BFI, Bowel Function Index; BSFS, Bristol Stool Form Scale; CCS, Cleveland Constipation Scale; EA, electroacupuncture; MA, manual acupuncture; MOX, moxibustion; OSM, osmotic laxatives; PAC-SYM, Patient Assessment of Constipation-Symptoms; PN, press needle; SA, sham acupuncture; SBMs, spontaneous bowel movements per week; STI, stimulant laxatives; TEAS, transcutaneous electrical acupoint stimulation.

The evidence was derived from both individual RCTs and pairwise meta-analyses. Compared with OSM, EA, PN, MA, PN+OSM, and MA+OSM each showed greater reductions in CCS scores in single studies. In contrast, APA and APA+OSM were evaluated in multiple trials and were synthesized using pairwise meta-analysis. However, substantial heterogeneity was observed, and the results should be interpreted with caution. Additionally, one trial reported that moxibustion was more beneficial than stimulant laxatives. Detailed results are presented in Supplementary materials 3A, 5A. Eleven interventions from 14 RCTs were included in the CCS network (Figure 3A). Compared with OSM, several interventions showed numerically greater reductions in CCS scores, but none of the network estimates reached statistical significance (Figure 4A). The SUCRA indicated that EA had the highest ranking probability for reducing CCS scores (SUCRA 0.85, Figure 5A).

##### 3.4.1.2 Weekly SBMs

The evidence was only derived from individual RCTs. Compared with OSM, EA, APA, and MOX+OSM each showed greater increases in weekly SBMs in single studies. In addition, EA was more beneficial than SA, and APA was more effective than STI. Detailed results are presented in Supplementary materials 3B, 5B. Five interventions from 5 RCTs were included in the weekly SBMs network (Figure 3B). The NMA results showed that MOX+OSM, EA, and APA showed numerically greater increases than OSM, but none of the comparisons reached statistical significance (Figure 4B). The SUCRA results suggested that MOX+OSM showed the highest ranking probability for increasing weekly SBMs (SUCRA 0.71, Figure 5B).

##### 3.4.1.3 BFI

The evidence was derived from individual RCTs. Compared with OSM, AA+OSM, APA+OSM, and AP+OSM each showed greater reductions in BFI scores in single studies, indicating improved bowel function. Four interventions from 3 RCTs were included in the BFI network (Figure 3C). Compared with OSM, AA+OSM (MD = −27.72, 95% CrI: −33.94, −21.41; low certainty of evidence), AP+OSM (MD = −13.34, 95% CrI: −15.60, −11.20; low certainty of evidence), and APA+OSM (MD = −10.57, 95% CrI: −13.33, −7.80; low certainty of evidence) showed statistically significant reductions in BFI scores (Figure 4C). According to the SUCRA results, AA+OSM showed the highest ranking probability for BFI improvement (SUCRA 1.00, Figure 5C).

##### 3.4.1.4 PAC-SYM

The evidence was derived from both individual RCTs and pairwise meta-analyses. In a single study, APA showed a statistically significant reduction in PAC-SYM scores compared with OSM, indicating an improvement in constipation-related symptoms. In addition, two trials comparing APA with BC were synthesized using pairwise meta-analysis, which demonstrated that APA (MD = −0.62, 95% CI: −0.89, −0.34; *I*^2^ = 87%; very low certainty of evidence) was associated with a greater reduction in PAC-SYM scores than BC. However, substantial heterogeneity was observed, and the pooled results should therefore be interpreted with caution. Detailed results are presented in Supplementary materials 3J, 5J. Three interventions from 3 RCTs were included in the PAC-SYM network (Figure 3D). According to the NMA results, none of the comparisons reached statistical significance (Figure 4D). The results of SUCRA suggested that APA had the highest ranking probability for PAC-SYM improvement (SUCRA 0.76, Figure 5D).

##### 3.4.1.5 BSFS

The evidence was assessed using both individual RCTs and pairwise meta-analyses. In single studies, MOX showed a statistically significant improvement in BSFS scores compared with OSM, while APA and AP showed statistically significant improvements compared with BC. Pairwise meta-analysis showed that PN+OSM (MD = 1.06, 95% CI: 0.73, 1.40; *I*^2^ = 0%; low certainty of evidence) was associated with a significantly greater improvement in BSFS scores compared with OSM. For APA versus OSM, the pooled estimate showed a small but statistically significant lower BSFS score in the APA group (MD = −0.30, 95% CI: −0.55, −0.04; *I*^2^ = 47%), which favored OSM. In contrast, APA+OSM did not show a statistically significant difference compared with OSM (MD = 0.07, 95% CI: −1.24, 1.39), and substantial heterogeneity was observed (*I*^2^ = 99%). Therefore, these findings should be interpreted with caution. Detailed results are presented in Supplementary materials 3K, 5K. Ten interventions from 13 RCTs were included in the BSFS network (Figure 3E). According to the NMA results, no comparison reached statistical significance (Figure 4E). The SUCRA results indicated that PN+OSM showed the highest ranking probability for BSFS improvement (SUCRA 0.71, Figure 5E). Sensitivity analysis using the imputed SD for Qi 2021 (41) produced a directionally similar but attenuated pairwise estimate whose 95% CI crossed the null, while the NMA estimate and treatment rankings remained essentially unchanged (Supplementary material 11).

#### 3.4.2 Secondary outcomes

##### 3.4.2.1 PAC-QOL

The evidence was assessed using both individual RCTs and pairwise meta-analyses. In single studies, PN, MOX+OSM, AA+OSM, APA+OSM, and PN+OSM showed statistically significant improvements in PAC-QOL scores compared with OSM. For APA versus OSM, three trials were available for pairwise meta-analysis; however, substantial heterogeneity was observed (*I*^2^ = 97%), and the pooled estimate did not indicate a statistically significant difference; therefore, these findings should be interpreted with caution. Detailed results of the PAC-QOL total score and its subscales are presented in Supplementary materials 3L–P, 5L–P. Nine interventions from 10 RCTs were included in the PAC-QOL network (Supplementary material 6G). According to the NMA results, none of the comparisons for the PAC-QOL total score reached statistical significance (Supplementary material 4F). Although AA+OSM, MOX+OSM, and PN+OSM showed numerically greater reductions in PAC-QOL scores than OSM, their 95% CrIs included the null value. The results of SUCRA indicated that MOX+OSM had the highest ranking probability for PAC-QOL improvement (SUCRA 1.00, Supplementary material 7G). Detailed results of the PAC-QOL subscales are presented in Supplementary materials 4G–J, 6H–K, 7H–K.

##### 3.4.2.2 BFI subscales

The BFI subscale analyses were considered secondary outcomes and are presented in Supplementary materials 3D–I, 4A–E, 5D–I, 6A–F, 7A–F.

### 3.5 Publication bias

Comparison-adjusted funnel plots were generated for CCS, BSFS, and PAC-QOL. Visual asymmetry was observed in some plots, suggesting possible small-study effects; however, these findings should be interpreted cautiously because the networks were sparse and included multiple treatment comparisons. Egger’s test was not performed because no individual pairwise comparison included at least 10 studies (Supplementary materials 8A–C).

### 3.6 Adverse events

Among the 36 included RCTs, 18 studies reported safety outcomes, of which 8 reported no adverse events. Overall, a numerically greater number of gastrointestinal adverse events was reported in the control groups than in the acupuncture groups. In 9 studies (28, 31, 33, 34, 39, 41, 45, 50, 52), the acupuncture groups mainly reported mild gastrointestinal symptoms and skin-related allergic reactions, including a total of 9 cases of diarrhea, 10 cases of vomiting, and 15 cases of skin allergic reactions. In contrast, the corresponding laxative groups reported a higher number of gastrointestinal adverse events, including 26 cases of diarrhea, 16 cases of vomiting, 4 cases of abdominal distension, and 2 cases of nausea. In addition, one study (60) recorded 2 cases of local hematoma and 1 case of bleeding at needling sites in the acupuncture group, whereas 5 adverse events were reported in the sham acupuncture group.

## 4 Discussion

### 4.1 Main findings

This systematic review included 36 RCTs involving 3,351 participants and evaluated the efficacy and safety of 15 acupuncture-related interventions for cancer-related OIC using pairwise meta-analysis and NMA.

Direct evidence from pairwise meta-analyses and single-study comparisons suggested that acupuncture-related therapies, either alone or combined with OSM, may improve CCS scores, weekly SBMs, BFI and its subscales, PAC-SYM, and BSFS compared with OSM or other controls. However, several estimates were based on limited evidence and showed substantial heterogeneity.

The NMA indicated that AA+OSM, AP+OSM, and APA+OSM were associated with statistically significant reductions in BFI scores compared with OSM alone. However, these estimates were derived from a sparse BFI network based on only three RCTs and were supported by low certainty of evidence. Although AA+OSM showed the highest SUCRA ranking probability for BFI improvement, this ranking should be interpreted as an exploratory signal rather than definitive evidence of comparative superiority. Therefore, greater emphasis should be placed on the effect estimates, 95% CrIs, number of contributing studies, and GRADE certainty rather than the ranking alone. APA was also associated with an improvement in the personal judgment of constipation item of the BFI compared with BC, although this finding was based on limited evidence.

Regarding constipation-related quality of life, this study provides novel evidence focusing on the “interference in life” dimension, as measured by the PAC-QOL total score and its subscales. Although several single-study direct comparisons suggested improvements in PAC-QOL total scores for PN, APA+OSM, MOX+OSM, AA+OSM, and PN+OSM compared with OSM, the NMA did not identify statistically significant differences between any intervention and OSM for the PAC-QOL total score. Some network estimates for selected PAC-QOL subscales suggested potential benefits compared with BC; however, these findings were based on sparse evidence and should be interpreted cautiously. To date, no systematic reviews or meta-analyses have specifically investigated the effects of acupuncture-related therapies on quality of life in cancer patients with OIC. Thus, our findings provide preliminary evidence addressing an important evidence gap, although further well-designed RCTs are required to confirm these findings.

Among the 18 RCTs reporting safety outcomes, adverse events associated with acupuncture-related interventions were generally mild and mainly included gastrointestinal symptoms and local skin reactions. However, because only half of the included RCTs reported safety outcomes, firm conclusions regarding comparative safety cannot be drawn.

### 4.2 Comparison with previous studies

In our study, both the direct evidence and NMA suggested that AA+OSM was associated with a reduction in BFI scores compared with OSM alone. Although AA+OSM had the highest SUCRA value for BFI improvement, this finding was derived from a sparse network and low-certainty evidence and should therefore be regarded as hypothesis-generating rather than as evidence that AA+OSM is the most effective intervention. Consistent with our findings, a previous meta-analysis on AA for adult functional constipation reported that AA combined with conventional treatment, including mosapride citrate, increased complete spontaneous bowel movements (CSBMs). However, differences in the study populations and comparators limit direct comparison with cancer-related OIC. APA also showed potential benefits for the personal judgment of constipation subscale of the BFI compared with BC, although this finding was based on limited evidence.

Notably, a previous meta-analysis evaluating APA for chronic functional constipation reported that APA monotherapy was significantly superior to laxatives in improving BSFS scores (63). However, our pairwise meta-analysis showed a small but statistically significant lower BSFS score for APA than for OSM, which favored OSM under the prespecified outcome direction, whereas the NMA did not show a statistically significant difference between APA and OSM. Several factors may explain this discrepancy. First, our study focused specifically on cancer-related OIC, which differs from chronic functional constipation in both pathophysiology and clinical context. OIC is primarily driven by opioid-mediated gastrointestinal dysmotility (8) and often coexists with multiple cancer-related factors, such as tumor burden, anticancer treatments, and reduced physical activity (64, 65), all of which may influence treatment response. Second, the relatively stringent inclusion criteria and the focus on a specific patient population resulted in a limited sample size. Third, unlike previous studies that grouped various laxatives into a single control category, our study subdivided laxatives based on their mechanisms of action. Although this classification improved the clinical specificity of the comparisons, it reduced the amount of evidence within individual comparisons and may have decreased the precision of the network estimates. Lastly, earlier studies typically focused on limited acupuncture techniques, restricting the generalizability of their results.

Previous RCTs have demonstrated that acupuncture-related therapies effectively improve stool-related outcomes (e.g., BSFS) (60, 66–70), bowel movement-related outcomes (e.g., CCS, weekly SBMs, ease of defecation score, feeling of incomplete bowel evacuation score in BFI, and PAC-SYM) (60, 62, 66–69, 71), and subscales of PAC-QOL (60, 66, 69, 71–73) in patients with constipation. In the present NMA, however, most comparisons for CCS, weekly SBMs, PAC-SYM, BSFS, and the PAC-QOL total score did not reach statistical significance. Therefore, the comparative effectiveness of individual acupuncture-related therapies remains uncertain and requires confirmation in larger, well-designed trials.

Given the multifactorial pathophysiology of OIC in patients with cancer, acupuncture-related therapies may exert their effects through multiple regulatory pathways rather than a single mechanism. Existing evidence suggests that acupuncture-related therapies may alleviate OIC symptoms primarily by enhancing intestinal motility and modulating neuro–enteric regulation (74, 75). Although different acupuncture-related techniques may share common pathways, they are likely to emphasize distinct modes of stimulation (76). Specifically, AA (77) has been reported to improve autonomic nervous function by activating the vagus nerve and modulating the brain–gut axis. APA (41) exerts its therapeutic effects by regulating the interstitial cells of Cajal, nitric oxide synthesis, and aquaporin expression. EA (78, 79) provides stronger and more consistent neuromodulatory input, enhancing enteric neural signaling and smooth muscle contractility. Complementarily, MOX (80) acts through thermal stimulation, promoting local microcirculation and activating visceral reflexes associated with bowel function. The specific mechanisms of acupuncture likely vary by technique and patient population, necessitating further mechanistic and translational investigation.

The measurement properties of the outcome instruments should also be considered when interpreting the findings. According to the COSMIN framework, relevant measurement properties include content validity, reliability, measurement error, responsiveness, and cross-cultural validity (81, 82). The outcomes included in this review assessed related but distinct constructs, including bowel movement frequency, stool form, constipation symptom severity, bowel function, and constipation-related quality of life. Although the BFI and PAC-SYM have supporting validation evidence in OIC (83, 84), and minimal clinically important differences have been proposed for PAC-SYM and PAC-QOL (85), not all instruments were validated specifically in patients with cancer-related OIC. Some instruments were developed or validated in broader constipation populations, functional gastrointestinal disorders, or specific language and cultural settings. Differences in constructs, response scales, recall periods, validated language versions, responsiveness, and clinically meaningful thresholds may therefore have contributed to measurement heterogeneity across trials.

This issue is particularly relevant because many outcomes were patient-reported or clinician-administered symptom scales. In acupuncture-related trials with limited participant blinding, subjective outcomes may be influenced by treatment expectations, preferences, patient–practitioner interactions, and cultural familiarity with acupuncture (86), in addition to physiological treatment effects. These factors may increase the risk of response bias or overestimation of treatment effects. Moreover, the included trials did not consistently report whether validated language versions were used or whether prespecified thresholds represented clinically meaningful improvement. Future trials should therefore use validated, disease-appropriate instruments, report their measurement properties and language versions, define minimal clinically important differences when available, and combine patient-reported outcomes with more objective indicators such as spontaneous bowel movements, rescue laxative use, and bowel diaries.

### 4.3 Strengths and limitations

This study offers several key strengths. First, the literature search was thorough and methodologically sound, covering both Chinese and English databases. Most of the included studies were published within the last decade and adopted internationally recognized diagnostic criteria. Second, this study maximally included acupuncture-related interventions, with clear definitions and systematic categorization. By excluding studies that involved combined use of Chinese herbal medicine, the study minimized potential confounding and enhanced the interpretability of results. Third, it adopted PROMs as the primary evaluation framework, ensuring data consistency and strong clinical relevance. As the first NMA to systematically integrate both direct and indirect evidence on acupuncture for OIC, it broadened the comparative evidence base, although the sparse networks limited the robustness of the conclusions. The entire research process adhered to rigorous methodological standards, with all reviewers undergoing formal training. A high level of consistency and accuracy was maintained throughout study selection, data extraction, and statistical analysis.

This study has several limitations as well. First, most included studies were conducted in Asian countries, which may limit the generalizability of the findings to populations in other regions and to different healthcare systems and sociocultural contexts. Regional variation in the distribution of cancer types may further limit the cross-regional applicability of these findings (87). Greater cultural familiarity with acupuncture in Asian settings may also influence treatment expectations, preferences, adherence, and responses to patient-reported outcomes (86). Second, most included RCTs had small sample sizes, and several interventions were supported by only a single trial. The evidence networks were consequently sparse, statistical precision was limited, and the certainty of evidence was generally low or very low. These limitations may have increased the risk of exaggerated treatment effects and reduced the reliability of indirect comparisons. In addition, because the networks contained no closed loops, global inconsistency could not be formally assessed. Third, substantial clinical and methodological variation was observed across studies. Acupuncture protocols differed in technique, acupoint selection, treatment frequency, stimulation or retention time, and treatment duration. Notable heterogeneity was observed in certain outcomes, particularly CCS, PAC-SYM, BSFS, and PAC-QOL. This heterogeneity is likely attributable to clinical and methodological differences across trials, such as baseline constipation severity, cancer types, and details of acupuncture-related interventions. Cancer stage, opioid type and dose, baseline constipation severity, concomitant treatments, and rescue regimens were also incompletely reported or varied across comparisons. As summarized in Supplementary material 10, most direct comparisons therefore raised some or major concerns regarding transitivity. Because the networks were sparse and many comparisons were informed by single studies, subgroup analyses, meta-regression, and sensitivity analyses based on potential effect modifiers were not feasible. Residual intransitivity and within-class heterogeneity related to differences in laxative agents, doses, frequencies, and rescue treatments cannot therefore be excluded. Finally, many outcomes were patient-reported and were assessed in trials in which participant blinding was limited or not feasible. Together with concerns regarding missing outcome data, this may have introduced measurement bias and affected the reliability of the estimates. SUCRA rankings may also be unstable in sparse networks and may be strongly influenced by single studies, small sample sizes, or imprecise estimates. They should therefore be regarded as exploratory and interpreted together with effect estimates, 95% CrIs, the number of contributing studies, and GRADE certainty rather than being used as standalone evidence of comparative superiority.

## 5 Conclusion

AA+OSM, AP+OSM, and APA+OSM may reduce BFI scores compared with OSM alone, and AP and APA may improve selected dimensions of constipation-related quality of life compared with BC in patients with cancer-related OIC. Among the evaluated interventions, AA+OSM had the highest probability of reducing BFI scores, whereas APA showed the highest ranking probabilities across the PAC-QOL subscales. However, the certainty of the evidence supporting these findings was low to very low, limiting confidence in the comparative effect estimates and treatment rankings. Further large-scale, high-quality RCTs with standardized protocols are needed to confirm these findings.

## Data Availability

The original contributions presented in the study are included in the article/Supplementary material, further inquiries can be directed to the corresponding authors.
