# Supplementary material for: Acupuncture-related therapies for opioid-induced constipation in patients with cancer: a systematic review and network meta-analysis
Source: Front Med (Lausanne). 2026 Aug 26;13:1869529. doi: 10.3389/fmed.2026.1869529 (PMC13553422; doi:10.3389/fmed.2026.1869529)
Supplement: Supplementary file 1 [file Supplementary_file_1.docx]

**Content**

[Supplement 1. Search strategy used in each database	4](#_Toc28798)

[The search strategy for the PubMed database	4](#_Toc15518)

[The search strategy for the Web of Science database	6](#_Toc29221)

[The search strategy for Embase database	9](#_Toc8461)

[The search strategy for the Cochrane Library database	10](#_Toc3655)

[The search strategy for China National Knowledge Infrastructure (CNKI) database	11](#_Toc24436)

[The search strategy for the Chinese Biological Medicine (CBM) database	13](#_Toc19789)

[The search strategy for the Chinese Science and Technology Periodical (VIP) database	15](#_Toc7119)

[The search strategy for Wan Fang database	17](#_Toc29591)

[Supplement 2. Excluded studies after full-text review	18](#_Toc16395)

[Supplement 3. Pairwise meta-analysis and single-study direct comparisons	26](#_Toc17975)

[Supplement 3A. CCS	26](#_Toc31816)

[Supplement 3B. Weekly SBMs	27](#_Toc7879)

[Supplement 3C. BFI	27](#_Toc19941)

[Supplement 3D. BFI: Feeling of incomplete bowel evacuation (1)	27](#_Toc3751)

[Supplement 3E. BFI: Feeling of incomplete bowel evacuation (2)	28](#_Toc27708)

[Supplement 3F. BFI: Ease of defecation (1)	28](#_Toc5472)

[Supplement 3G. BFI: Ease of defecation (2)	28](#_Toc12281)

[Supplement 3H. BFI: Personal judgement of constipation (1)	28](#_Toc19211)

[Supplement 3I. BFI: Personal judgement of constipation (2)	29](#_Toc29297)

[Supplement 3J. PAC-SYM	29](#_Toc9951)

[Supplement 3K. BSFS	29](#_Toc3172)

[Supplement 3L. PAC-QOL	30](#_Toc2909)

[Supplement 3M. PAC-QOL: Worries and concerns	30](#_Toc6151)

[Supplement 3N. PAC-QOL: Physical	31](#_Toc19637)

[Supplement 3O. PAC-QOL: Satisfaction	31](#_Toc30810)

[Supplement 3P. PAC-QOL: Psychosocial	31](#_Toc19275)

[Supplement 4. League Table for Network Meta-Analysis	32](#_Toc30728)

[Supplement 4A. BFI: Feeling of incomplete bowel evacuation (1)	32](#_Toc19346)

[Supplement 4B. BFI: Feeling of incomplete bowel evacuation (2)	32](#_Toc9314)

[Supplement 4C. BFI: Ease of defecation (2)	33](#_Toc15766)

[Supplement 4D. BFI: Personal judgement of constipation (1)	34](#_Toc27968)

[Supplement 4E. BFI: Personal judgement of constipation (2)	34](#_Toc31203)

[Supplement 4F. PAC-QOL	35](#_Toc6339)

[Supplement 4G. PAC-QOL: Worries and concerns	36](#_Toc26296)

[Supplement 4H. PAC-QOL: Physical	37](#_Toc21071)

[Supplement 4I. PAC-QOL: Satisfaction	38](#_Toc20378)

[Supplement 4J. PAC-QOL: Psychosocial	39](#_Toc5689)

[Supplement 5. Certainty of evidence (GRADE)	40](#_Toc19145)

[Supplement 5A. CCS	40](#_Toc2044)

[Supplement 5B. Weekly SBMs	50](#_Toc9005)

[Supplement 5C. BFI	53](#_Toc28397)

[Supplement 5D. BFI: Feeling of incomplete bowel evacuation (1)	54](#_Toc12963)

[Supplement 5E. BFI: Feeling of incomplete bowel evacuation (2)	54](#_Toc13772)

[Supplement 5F. BFI: Ease of defecation (1)	55](#_Toc14305)

[Supplement 5G. BFI: Ease of defecation (2)	55](#_Toc27556)

[Supplement 5H. BFI: Personal judgement of constipation (1)	56](#_Toc19525)

[Supplement 5I. BFI: Personal judgement of constipation (2)	56](#_Toc30932)

[Supplement 5J. PAC-SYM	58](#_Toc328)

[Supplement 5K. BSFS	59](#_Toc4407)

[Supplement 5L. PAC-QOL	67](#_Toc10869)

[Supplement 5M. PAC-QOL: Worries and concerns	73](#_Toc29899)

[Supplement 5N. PAC-QOL: Physical	75](#_Toc16392)

[Supplement 5O. PAC-QOL: Satisfaction	77](#_Toc23107)

[Supplement 5P. PAC-QOL: Psychosocial	79](#_Toc26538)

[Supplement 6. Network plot	81](#_Toc31587)

[Supplement 6A. BFI: Feeling of Incomplete Bowel Evacuation (1)	81](#_Toc23519)

[Supplement 6B. BFI: Feeling of Incomplete Bowel Evacuation (2)	81](#_Toc14625)

[Supplement 6C. BFI: Ease of Defecation (1)	82](#_Toc28712)

[Supplement 6D. BFI: Ease of Defecation (2)	82](#_Toc15833)

[Supplement 6E. BFI: Personal judgement of constipation (1)	83](#_Toc7908)

[Supplement 6F. BFI: Personal judgement of constipation (2)	83](#_Toc30233)

[Supplement 6G. PAC-QOL	84](#_Toc14189)

[Supplement 6H. PAC-QOL: Worries and concerns	85](#_Toc15563)

[Supplement 6I. PAC-QOL: Physical	86](#_Toc13840)

[Supplement 6J. PAC-QOL: Satisfaction	87](#_Toc15826)

[Supplement 6K. PAC-QOL: Psychosocial	88](#_Toc23756)

[Supplement 7. SUCRA plot	89](#_Toc27211)

[Supplement 7A. BFI: Feeling of Incomplete Bowel Evacuation (1)	89](#_Toc6908)

[Supplement 7B. BFI: Feeling of Incomplete Bowel Evacuation (2)	89](#_Toc29709)

[Supplement 7C. BFI: Ease of Defecation (1)	90](#_Toc856)

[Supplement 7D. BFI: Ease of Defecation (2)	90](#_Toc12849)

[Supplement 7E. BFI: Personal judgement of constipation (1)	91](#_Toc19180)

[Supplement 7F. SUCRA plot: BFI: Personal judgement of constipation (2)	91](#_Toc7827)

[Supplement 7G. PAC-QOL	92](#_Toc24835)

[Supplement 7H. PAC-QOL: Worries and concerns	93](#_Toc23733)

[Supplement 7I. PAC-QOL: Physical	94](#_Toc28429)

[Supplement 7J. PAC-QOL: Satisfaction	95](#_Toc31852)

[Supplement 7K. PAC-QOL: Psychosocial	96](#_Toc18695)

[Supplement 8. Funnel plot	97](#_Toc32573)

[Supplement 8A. Funnel plot: CCS	97](#_Toc23035)

[Supplement 8B. Funnel plot: BSFS	98](#_Toc7506)

[Supplement 8C. Funnel plot: PAC-QOL	99](#_Toc13701)

[Supplement 9. Detailed RoB 2 judgments and supporting reasons for each included study	100](#_Toc28531)

[Supplement 10. Assessment of transitivity and potential effect modifiers across treatment comparisons	110](#_Toc32390)

[Supplement 11. Handling of the zero standard deviation value and sensitivity analysis for BSFS	122](#_Toc3430)

**Supplement 1. Search strategy used in each database**

**The search strategy for the PubMed database**

The retrieval of the PubMed database was conducted on June 28, 2025, and 21 records was retrieved.

| **No** | **Search History** |
| --- | --- |
| #1 | "Opioid-Induced Constipation"[Mesh] OR "Constipation"[Mesh] OR "Gastrointestinal Transit"[Mesh] OR Constipation[tiab] OR "bowel dysfunction"[tiab] OR "Gastrointestinal Transit"[tiab] OR "slow transit"[tiab] |
| #2 | "Analgesics, Opioid"[Mesh] OR "Opiate Alkaloids"[Mesh] OR opioid[tiab] OR opiate*[tiab] OR narcotic-analgesic*[tiab] OR heroin*[tiab] OR diamorphine[tiab] OR codeine*[tiab] OR morphin*[tiab] OR Hydrocodone[tiab] OR Oxycodone[tiab] OR Oxymorphone[tiab] OR Fentanyl[tiab] OR Hydromorphone[tiab] OR Tapentadol[tiab] OR Methadone[tiab] OR Buprenorphine[tiab] OR Tramadol[tiab] OR Vicodin[tiab] OR Oxycontin[tiab] OR Percocet[tiab] OR Opana[tiab] OR Kadian[tiab] OR Avinza[tiab] |
| #3 | (#1 AND #2) |
| #4 | "Medicine, Chinese Traditional"[Mesh] OR "Acupuncture Therapy"[Mesh] OR "Acupuncture"[Mesh] OR "Electroacupuncture"[Mesh] OR "Acupuncture Points"[Mesh] OR "Acupuncture, Ear"[Mesh] OR "Moxibustion"[Mesh] OR "Acupressure"[Mesh] OR "transcutaneous electric nerve stimulation"[Mesh] OR "Massage"[Mesh] OR "Cupping Therapy"[Mesh] OR "Herbal Medicine"[Mesh] OR acupunc*[tiab] OR needl*[tiab] OR electroacupuncture*[tiab] OR electro-acupuncture*[tiab] OR acupoint*[tiab] OR "fire needle"[tiab] OR "scalp needle"[tiab] OR ((catgut[tiab] OR thread[tiab] OR acupoint[tiab] OR therap*[tiab]) AND embed*[tiab]) OR ((pharmaco*[tiab] OR filiform[tiab] OR three-edged[tiab] OR blood* [tiab] OR bleed* [tiab] OR plum-blossom[tiab] OR auricular[tiab] OR ear[tiab] OR cutaneous[tiab] OR intradermal[tiab] OR intracutaneous[tiab] OR subcutaneous[tiab] OR press[tiab] OR thumb-tack[tiab] OR dry[tiab] OR warm[tiab] OR heated[tiab] OR thermal[tiab] OR moxibus*[tiab] OR embed*[tiab] OR thread[tiab]) AND (needl*[tiab] OR acupunc*[tiab])) OR Moxabustion*[tiab] OR Moxibustion*[tiab] OR acupressure*[tiab] OR shiatsu[tiab] OR "zhi ya"[tiab] OR TENS[tiab] OR "transcutaneous electric nerve stimulation"[tiab] OR massage*[tiab] OR "tui na"[tiab] OR tui-na[tiab] OR tuina[tiab] OR "zone therap*"[tiab] OR cupping*[tiab] OR "gua sha"[tiab] OR "scraping therap*"[tiab] OR "auricular plaster*"[tiab] OR "auricular point sticking"[tiab] OR "ear seed*"[tiab] OR "ear acupressure"[tiab] OR "acupoint sticking"[tiab] OR "hot compress*"[tiab] OR "herbal compress*"[tiab] OR "acupoint patch*"[tiab] OR "plaster therap*"[tiab] OR "herbal plaster*"[tiab] OR "Acupoint Application"[tiab] OR "Medicinal Plaster"[tiab] OR "acupoint injection"[tiab] OR "herbal injection"[tiab] OR "pharmacological injection"[tiab] OR "medicated bath"[tiab] OR "herbal bath"[tiab] OR "foot bath"[tiab] OR "herbal foot bath"[tiab] |
| #5 | ("Controlled Clinical Trial"[Publication Type] OR "Randomized Controlled Trial"[Publication Type] OR "Controlled Clinical Trials as Topic"[Mesh] OR "Randomized Controlled Trials as Topic"[Mesh] OR "Randomized Controlled Trials"[tiab] OR random*[tiab] OR placebo[tiab] OR "trial"[tiab]) NOT ("Animals"[Mesh] NOT "Humans"[Mesh]) |
| #6 | (#5 NOT ("Systematic Review"[Publication Type] OR "Systematic Reviews as Topic"[Mesh] OR "Systematic Review"[tiab] OR "Meta-Analysis"[Publication Type] OR "Meta-Analysis as Topic"[Mesh] OR "Meta-Analysis"[tiab])) |
| #7 | (#3 AND #4 AND #6) |

**The search strategy for the Web of Science database**

The retrieval of the WOS database was conducted on June 28, 2025, and 33 records was retrieved.

| **No** | **Search History** |
| --- | --- |
| #1 | TS=(Constipation OR "Bowel Syndrome*" OR "Bowel Dysfunction*" OR "Gastrointestinal Transit" OR "Slow Transit") OR TI=(Constipation OR "Bowel Syndrome*" OR "Bowel Dysfunction*" OR "Gastrointestinal Transit" OR "Slow Transit") OR AB=(Constipation OR "Bowel Syndrome*" OR "Bowel Dysfunction*" OR "Gastrointestinal Transit" OR "Slow Transit") |
| #2 | TS=(opioid* OR opiate* OR narcotic-analgesic* OR heroin* OR diamorphine OR codeine* OR morphin* OR Hydrocodone OR Oxycodone OR Oxymorphone OR Fentanyl OR Hydromorphone OR Tapentadol OR Methadone OR Buprenorphine OR Tramadol OR Vicodin OR Oxycontin OR Percocet OR Opana OR Kadian OR Avinza) OR TI=(opioid* OR opiate* OR narcotic-analgesic* OR heroin* OR diamorphine OR codeine* OR morphin* OR Hydrocodone OR Oxycodone OR Oxymorphone OR Fentanyl OR Hydromorphone OR Tapentadol OR Methadone OR Buprenorphine OR Tramadol OR Vicodin OR Oxycontin OR Percocet OR Opana OR Kadian OR Avinza) OR AB=(opioid* OR opiate* OR narcotic-analgesic* OR heroin* OR diamorphine OR codeine* OR morphin* OR Hydrocodone OR Oxycodone OR Oxymorphone OR Fentanyl OR Hydromorphone OR Tapentadol OR Methadone OR Buprenorphine OR Tramadol OR Vicodin OR Oxycontin OR Percocet OR Opana OR Kadian OR Avinza) |
| #3 | (#1 AND #2) |
| #4 | TS=("Acupuncture Therapy" OR Acupuncture OR "acupuncture, ear" OR Electroacupuncture OR "Acupuncture Points" OR Moxibustion OR Acupressure OR "transcutaneous electric nerve stimulation" OR Massage OR "Cupping Therapy" OR "Herbal Medicine" OR acupunc* OR needl* OR electroacupuncture* OR "electro acupuncture*" OR acupoint* OR "fire needle" OR "scalp needle" OR ((catgut OR thread OR acupoint OR therap*) AND embed*) OR ((pharmaco* OR filiform OR three-edged OR blood* OR bleed* OR plum-blossom OR auricular OR ear OR cutaneous OR intradermal OR intracutaneous OR subcutaneous OR press OR thumb-tack OR dry OR warm OR heated OR thermal OR moxibus* OR embed* OR thread) AND (needl* OR acupunc*)) OR moxabustion* OR moxibustion* OR acupressure* OR shiatsu OR "zhi ya" OR TENS OR "transcutaneous electric nerve stimulation" OR massage* OR tui-na OR "tui na" OR tuina OR "zone therap*" OR cupping* OR "gua sha" OR "scraping therap*" OR "auricular plaster*" OR "auricular point sticking" OR "ear seed*" OR "ear acupressure" OR "acupoint sticking" OR "hot compress*" OR "herbal compress*" OR "acupoint patch*" OR "plaster therap*" OR "herbal plaster*" OR "Acupoint Application" OR "Medicinal Plaster" OR "acupoint injection" OR "herbal injection" OR "pharmacological injection" OR "medicated bath" OR "herbal bath" OR "foot bath" OR "herbal foot bath") OR TI=("Acupuncture Therapy" OR Acupuncture OR "acupuncture, ear" OR Electroacupuncture OR "Acupuncture Points" OR Moxibustion OR Acupressure OR "transcutaneous electric nerve stimulation" OR Massage OR "Cupping Therapy" OR "Herbal Medicine" OR acupunc* OR needl* OR electroacupuncture* OR "electro acupuncture*" OR acupoint* OR "fire needle" OR "scalp needle" OR ((catgut OR thread OR acupoint OR therap*) AND embed*) OR ((pharmaco* OR filiform OR three-edged OR blood* OR bleed* OR plum-blossom OR auricular OR ear OR cutaneous OR intradermal OR intracutaneous OR subcutaneous OR press OR thumb-tack OR dry OR warm OR heated OR thermal OR moxibus* OR embed* OR thread) AND (needl* OR acupunc*)) OR moxabustion* OR moxibustion* OR acupressure* OR shiatsu OR "zhi ya" OR TENS OR "transcutaneous electric nerve stimulation" OR massage* OR tui-na OR "tui na" OR tuina OR "zone therap*" OR cupping* OR "gua sha" OR "scraping therap*" OR "auricular plaster*" OR "auricular point sticking" OR "ear seed*" OR "ear acupressure" OR "acupoint sticking" OR "hot compress*" OR "herbal compress*" OR "acupoint patch*" OR "plaster therap*" OR "herbal plaster*" OR "Acupoint Application" OR "Medicinal Plaster" OR "acupoint injection" OR "herbal injection" OR "pharmacological injection" OR "medicated bath" OR "herbal bath" OR "foot bath" OR "herbal foot bath") OR AB=("Acupuncture Therapy" OR Acupuncture OR "acupuncture, ear" OR Electroacupuncture OR "Acupuncture Points" OR Moxibustion OR Acupressure OR "transcutaneous electric nerve stimulation" OR Massage OR "Cupping Therapy" OR "Herbal Medicine" OR acupunc* OR needl* OR electroacupuncture* OR "electro acupuncture*" OR acupoint* OR "fire needle" OR "scalp needle" OR ((catgut OR thread OR acupoint OR therap*) AND embed*) OR ((pharmaco* OR filiform OR three-edged OR blood* OR bleed* OR plum-blossom OR auricular OR ear OR cutaneous OR intradermal OR intracutaneous OR subcutaneous OR press OR thumb-tack OR dry OR warm OR heated OR thermal OR moxibus* OR embed* OR thread) AND (needl* OR acupunc*)) OR moxabustion* OR moxibustion* OR acupressure* OR shiatsu OR "zhi ya" OR TENS OR "transcutaneous electric nerve stimulation" OR massage* OR tui-na OR "tui na" OR tuina OR "zone therap*" OR cupping* OR "gua sha" OR "scraping therap*" OR "auricular plaster*" OR "auricular point sticking" OR "ear seed*" OR "ear acupressure" OR "acupoint sticking" OR "hot compress*" OR "herbal compress*" OR "acupoint patch*" OR "plaster therap*" OR "herbal plaster*" OR "Acupoint Application" OR "Medicinal Plaster" OR "acupoint injection" OR "herbal injection" OR "pharmacological injection" OR "medicated bath" OR "herbal bath" OR "foot bath" OR "herbal foot bath") |
| #5 | TS=("Randomized Controlled Trial" OR "Controlled Clinical Trial" OR "random allocation" OR RCT OR random* OR placebo*) OR TI=("Randomized Controlled Trial" OR "Controlled Clinical Trial" OR "random allocation" OR RCT OR random* OR placebo*) OR AB=("Randomized Controlled Trial" OR "Controlled Clinical Trial" OR "random allocation" OR RCT OR random* OR placebo*) OR AK=("Randomized Controlled Trial" OR "Controlled Clinical Trial" OR "random allocation" OR RCT OR random* OR placebo*) |
| #6 | (#5 NOT (TS=("Systematic Review" OR "Meta-Analysis") OR TI=("Systematic Review" OR "Meta-Analysis") OR AB=("Systematic Review" OR "Meta-Analysis") OR AK=("Systematic Review" OR "Meta-Analysis"))) |
| #7 | (#3 AND #4 AND #6) |

**The search strategy for Embase database**

The retrieval of the Embase database was conducted on June 28, 2025, and a total of 90 records were retrieved.

| **No** | **Search History** |
| --- | --- |
| #1 | 'opioid induced constipation'/exp OR 'constipation'/exp OR 'gastrointestinal transit'/exp OR (Constipation OR 'bowel dysfunction' OR 'Gastrointestinal transit' OR 'slow transit'):ti,ab,kw |
| #2 | 'narcotic analgesic agent'/exp OR 'opiate'/exp OR (opioid* OR narcotic-analgesic* OR heroin* OR diamorphine OR codeine* OR morphin* OR Hydrocodone OR Oxycodone OR Oxymorphone OR Fentanyl OR Hydromorphone OR Tapentadol OR Methadone OR Buprenorphine OR Tramadol OR Vicodin OR Oxycontin OR Percocet OR Opana OR Kadian OR Avinza):ti,ab,kw |
| #3 | (#1 AND #2) |
| #4 | 'acupuncture'/exp OR 'electroacupuncture'/exp OR 'acupuncture point'/exp OR 'moxibustion'/exp OR 'acupressure'/exp OR 'transcutaneous electrical nerve stimulation'/exp OR 'massage'/exp OR 'cupping therapy'/exp OR 'herbal medicine'/exp OR (acupun* OR needl* OR electroacupuncture* OR electro-acupuncture* OR acupoint* OR 'fire needle' OR 'scalp needle' OR ((catgut OR thread OR acupoint OR therap*) AND embed*) OR ((pharmaco* OR filiform OR three-edged OR blood* OR bleed* OR plum-blossom OR auricular OR ear OR cutaneous OR intradermal OR intracutaneous OR subcutaneous OR press OR thumb-tack OR dry OR warm OR heated OR thermal OR moxibus* OR embed* OR thread) AND (needl* OR acupunc*)) OR Moxabustion* OR Moxibustion* OR acupressure* OR shiatsu OR shiatzu OR 'zhi ya' OR 'chih ya' OR TENS OR 'transcutaneous electric nerve stimulation' OR massage* OR 'tui na' OR tui-na OR tuina OR 'zone therap*' OR cupping* OR 'gua sha' OR 'scraping therap*' OR 'auricular plaster*' OR 'auricular point sticking' OR 'ear seed*' OR 'ear pellet*' OR 'ear acupressure' OR 'acupoint sticking' OR 'hot compress*' OR 'herbal compress*' OR 'medicated compress*' OR 'acupoint patch*' OR 'plaster therap*' OR 'herbal plaster*' OR 'Acupoint Application' OR 'Acupoint Herbal Patch' OR 'Medicinal Plaster' OR 'TCM Patch Therapy' OR 'Traditional Chinese Medicine Patch Therapy' OR 'External Herbal Patch' OR 'acupoint cataplasm*' OR 'acupoint injection' OR 'herbal injection' OR 'pharmacological injection' OR 'medicated bath' OR 'herbal bath' OR 'foot bath' OR 'herbal foot bath'):ti,ab,kw |
| #5 | ('randomized controlled trial'/exp OR randomization/exp OR (random* OR rct OR "random allocation" OR placebo):ti,ab,kw) NOT ([animals]/lim NOT [humans]/lim) |
| #6 | (#5 NOT ('systematic review'/exp OR 'systematic review (topic)'/exp OR 'meta analysis'/exp OR 'meta analysis (topic)'/exp)) |
| #7 | (#3 AND #4 AND #6) |

**The search strategy for the Cochrane Library database**

The retrieval of the Cochrane Library was conducted on June 28, 2025, and a total of 66 records were retrieved.

| **No** | **Search History** |
| --- | --- |
| #1 | (Constipation OR "bowel dysfunction" OR "gastrointestinal transit" OR "slow transit"):ti,ab,kw |
| #2 | (opioid* OR opiate* OR narcotic-analgesic* OR heroin* OR diamorphine OR codeine* OR morphin* OR Hydrocodone OR Oxycodone OR Oxymorphone OR Fentanyl OR Hydromorphone OR Tapentadol OR Methadone OR Buprenorphine OR Tramadol OR Vicodin OR Oxycontin OR Percocet OR Opana OR Kadian OR Avinza):ti,ab,kw |
| #3 | (#1 AND #2) |
| #4 | ("Acupuncture Therapy" OR Acupuncture OR "acupuncture, ear" OR Electroacupuncture OR "Acupuncture Points" OR Moxibustion OR Acupressure OR "transcutaneous electric nerve stimulation" OR Massage OR "Cupping Therapy" OR "Herbal Medicine" OR acupunc* OR needl* OR electroacupuncture* OR (electro NEXT acupuncture*) OR acupoint* OR "fire needle" OR "scalp needle" OR ((catgut OR thread OR acupoint OR therap*) AND embed*) OR ((pharmaco* OR filiform OR three-edged OR blood* OR bleed* OR plum-blossom OR auricular OR ear OR cutaneous OR intradermal OR intracutaneous OR subcutaneous OR press OR thumb-tack OR dry OR warm OR heated OR thermal OR moxibus* OR embed* OR thread) AND (needl* OR acupunc*)) OR moxabustion* OR moxibustion* OR acupressure* OR shiatsu OR "zhi ya" OR TENS OR "transcutaneous electric nerve stimulation" OR massage* OR tui-na OR "tui na" OR tuina OR (zone NEXT therap*) OR cupping* OR "gua sha" OR (scraping NEXT therap*) OR (auricular NEXT plaster*) OR "auricular point sticking" OR (ear NEXT seed*) OR "ear acupressure" OR "acupoint sticking" OR (hot NEXT compress*) OR (herbal NEXT compress*) OR (acupoint NEXT patch*) OR (plaster NEXT therap*) OR (herbal NEXT plaster*) OR "Acupoint Application" OR "Medicinal Plaster" OR "acupoint injection" OR "herbal injection" OR "pharmacological injection" OR "medicated bath" OR "herbal bath" OR "foot bath" OR "herbal foot bath"):ti,ab,kw |
| #5 | MeSH descriptor: ["Randomized Controlled Trial"] explode all trees |
| #6 | ("Controlled Clinical Trial" OR random* OR placebo):ti,ab,kw |
| #7 | (#5 OR #6) |
| #8 | #7 NOT ("Systematic Review" OR "Meta-Analysis"):ti,ab,kw |
| #9 | (#3 AND #4 AND #8) |

**The search strategy for China National Knowledge Infrastructure (CNKI) database**

The retrieval of the CNKI database was conducted on June 28, 2025, and a total of 219 records were retrieved.

| **No** | **Search History** |
| --- | --- |
| #1 | SU=('阿片类药物诱导便秘' + '药物相关性便秘' + '药物性便秘' + '便秘') OR TI=('阿片类药物诱导便秘' + '药物相关性便秘' + '药物性便秘' + '便秘') OR KY=('阿片类药物诱导便秘' + '药物相关性便秘' + '药物性便秘' + '便秘') OR AB=('阿片类药物诱导便秘' + '药物相关性便秘' + '药物性便秘' + '便秘') |
| #2 | SU=('镇痛药, 阿片类' + '镇痛药' + '阿片' + '麻醉性镇痛药' + '中枢性镇痛药' + '海洛因' + '二醋吗啡' + '吗啡' + '美施康定' + '美菲康' + '可待因' + '甲基吗啡' + '氢可酮' + '羟考酮' + '羟吗啡酮' + '氧吗啡酮' + '芬太尼' + '氢吗啡酮' + '他喷他多' + '美沙酮' + '丁丙诺啡' + '曲马多' + '曲马朵' + '维柯丁' + '维可定' + '奥施康定' + '硫酸吗啡') OR TI=('镇痛药, 阿片类' + '镇痛药' + '阿片' + '麻醉性镇痛药' + '中枢性镇痛药' + '海洛因' + '二醋吗啡' + '吗啡' + '美施康定' + '美菲康' + '可待因' + '甲基吗啡' + '氢可酮' + '羟考酮' + '羟吗啡酮' + '氧吗啡酮' + '芬太尼' + '氢吗啡酮' + '他喷他多' + '美沙酮' + '丁丙诺啡' + '曲马多' + '曲马朵' + '维柯丁' + '维可定' + '奥施康定' + '硫酸吗啡') OR KY=('镇痛药, 阿片类' + '镇痛药' + '阿片' + '麻醉性镇痛药' + '中枢性镇痛药' + '海洛因' + '二醋吗啡' + '吗啡' + '美施康定' + '美菲康' + '可待因' + '甲基吗啡' + '氢可酮' + '羟考酮' + '羟吗啡酮' + '氧吗啡酮' + '芬太尼' + '氢吗啡酮' + '他喷他多' + '美沙酮' + '丁丙诺啡' + '曲马多' + '曲马朵' + '维柯丁' + '维可定' + '奥施康定' + '硫酸吗啡') OR AB=('镇痛药, 阿片类' + '镇痛药' + '阿片' + '麻醉性镇痛药' + '中枢性镇痛药' + '海洛因' + '二醋吗啡' + '吗啡' + '美施康定' + '美菲康' + '可待因' + '甲基吗啡' + '氢可酮' + '羟考酮' + '羟吗啡酮' + '氧吗啡酮' + '芬太尼' + '氢吗啡酮' + '他喷他多' + '美沙酮' + '丁丙诺啡' + '曲马多' + '曲马朵' + '维柯丁' + '维可定' + '奥施康定' + '硫酸吗啡')精确） |
| #3 | SU=('针灸疗法' + '针刺' + '电针' + '针刺穴位' + '耳针疗法' + '耳穴贴压疗法' + '灸法' + '穴位按压' + '经皮神经电刺激' + '按摩' + '拔罐疗法' + '药熨疗法' + '热敷疗法' + '灸' + '穴' + '针灸' + '穴位' + '电针' + '针刀' + '火针' + '毫针' + '干针' + '微针' + '手针' + '头针' + '腹针' + '腕踝针' + '眼针' + '鼻针' + '唇针' + '耳针' + '浮针' + '揿针' + '温针' + '埋针' + '埋线' + '刺络' + '皮*针' + '梅花针' + '三棱针' + '激光针' + '艾灸' + '经皮*电刺激' + '推拿' + '拔罐' + '刮痧' + '埋豆' + '耳穴' + '药浴' + '足浴') OR TI=('针灸疗法' + '针刺' + '电针' + '针刺穴位' + '耳针疗法' + '耳穴贴压疗法' + '灸法' + '穴位按压' + '经皮神经电刺激' + '按摩' + '拔罐疗法' + '药熨疗法' + '热敷疗法' + '灸' + '穴' + '针灸' + '穴位' + '电针' + '针刀' + '火针' + '毫针' + '干针' + '微针' + '手针' + '头针' + '腹针' + '腕踝针' + '眼针' + '鼻针' + '唇针' + '耳针' + '浮针' + '揿针' + '温针' + '埋针' + '埋线' + '刺络' + '皮*针' + '梅花针' + '三棱针' + '激光针' + '艾灸' + '经皮*电刺激' + '推拿' + '拔罐' + '刮痧' + '埋豆' + '耳穴' + '药浴' + '足浴') OR KY=('针灸疗法' + '针刺' + '电针' + '针刺穴位' + '耳针疗法' + '耳穴贴压疗法' + '灸法' + '穴位按压' + '经皮神经电刺激' + '按摩' + '拔罐疗法' + '药熨疗法' + '热敷疗法' + '灸' + '穴' + '针灸' + '穴位' + '电针' + '针刀' + '火针' + '毫针' + '干针' + '微针' + '手针' + '头针' + '腹针' + '腕踝针' + '眼针' + '鼻针' + '唇针' + '耳针' + '浮针' + '揿针' + '温针' + '埋针' + '埋线' + '刺络' + '皮*针' + '梅花针' + '三棱针' + '激光针' + '艾灸' + '经皮*电刺激' + '推拿' + '拔罐' + '刮痧' + '埋豆' + '耳穴' + '药浴' + '足浴') OR AB=('针灸疗法' + '针刺' + '电针' + '针刺穴位' + '耳针疗法' + '耳穴贴压疗法' + '灸法' + '穴位按压' + '经皮神经电刺激' + '按摩' + '拔罐疗法' + '药熨疗法' + '热敷疗法' + '灸' + '穴' + '针灸' + '穴位' + '电针' + '针刀' + '火针' + '毫针' + '干针' + '微针' + '手针' + '头针' + '腹针' + '腕踝针' + '眼针' + '鼻针' + '唇针' + '耳针' + '浮针' + '揿针' + '温针' + '埋针' + '埋线' + '刺络' + '皮*针' + '梅花针' + '三棱针' + '激光针' + '艾灸' + '经皮*电刺激' + '推拿' + '拔罐' + '刮痧' + '埋豆' + '耳穴' + '药浴' + '足浴') |
| #4 | SU=('临床研究' + '临床试验' + '随机对照试验' + '对照试验' + '随机' + '疗效评价') OR TI=('临床研究' + '临床试验' + '随机对照试验' + '对照试验' + '随机' + '疗效评价') OR KY=('临床研究' + '临床试验' + '随机对照试验' + '对照试验' + '随机' + '疗效评价') OR AB=('临床研究' + '临床试验' + '随机对照试验' + '对照试验' + '随机' + '疗效评价') |
| #5 | (#1 AND #2 AND #3 AND #4) |
| #6 | SU=('动物' + '鼠' + 'Meta分析') OR TI=('动物' + '鼠' + 'Meta分析') OR KY=('动物' + '鼠' + 'Meta分析') OR AB=('动物' + '鼠' + 'Meta分析') |
| #7 | (#5 NOT #6) |

**The search strategy for the Chinese Biological Medicine (CBM) database**

The retrieval of the CBM database was conducted on June 28, 2025, and a total of 183 records were retrieved.

| **No** | **Search History** |
| --- | --- |
| #1 | "阿片类药物诱导便秘"[不加权:扩展] OR "胃肠通过试验"[不加权:扩展] OR "药物相关性便秘"[常用字段:智能] OR "药物性便秘"[常用字段:智能] OR "便秘"[常用字段:智能] |
| #2 | "镇痛药, 阿片类"[不加权:扩展] OR "镇痛药"[常用字段:智能] OR "阿片"[常用字段:智能] OR "麻醉性镇痛药"[常用字段:智能] OR "中枢性镇痛药"[常用字段:智能] OR "海洛因"[常用字段:智能] OR "二醋吗啡"[常用字段:智能] OR "吗啡"[常用字段:智能] OR "美施康定"[常用字段:智能] OR "美菲康"[常用字段:智能] OR "二醋吗啡"[常用字段:智能] OR "可待因"[常用字段:智能] OR "甲基吗啡"[常用字段:智能] OR "氢可酮"[常用字段:智能] OR "羟考酮"[常用字段:智能] OR "羟吗啡酮"[常用字段:智能] OR "氧吗啡酮"[常用字段:智能] OR "芬太尼"[常用字段:智能] OR "氢吗啡酮"[常用字段:智能] OR "他喷他多"[常用字段:智能] OR "美沙酮"[常用字段:智能] OR "丁丙诺啡"[常用字段:智能] OR "曲马多"[常用字段:智能] OR "曲马朵"[常用字段:智能] OR "维柯丁"[常用字段:智能] OR "维可定"[常用字段:智能] OR "奥施康定"[常用字段:智能] OR "硫酸吗啡"[常用字段:智能] |
| #3 | (#1 AND #2) |
| #4 | "针灸疗法"[不加权:扩展] OR "针刺"[不加权:扩展] OR "电针"[不加权:扩展] OR "针刺穴位"[不加权:扩展] OR "耳针疗法"[不加权:扩展] OR "耳穴贴压疗法"[不加权:扩展] OR "灸法"[不加权:扩展] OR "穴位按压"[不加权:扩展] OR "经皮神经电刺激"[不加权:扩展] OR "按摩"[不加权:扩展] OR "拔罐疗法"[不加权:扩展] OR "药熨疗法"[不加权:扩展] OR "热敷疗法"[不加权:扩展] OR "灸"[常用字段:智能] OR "穴"[常用字段:智能] OR "针灸"[常用字段:智能] OR "穴位"[常用字段:智能] OR "电针"[常用字段:智能] OR "针刀"[常用字段:智能] OR "火针"[常用字段:智能] OR "毫针"[常用字段:智能] OR "干针"[常用字段:智能] OR "微针"[常用字段:智能] OR "手针"[常用字段:智能] OR "头针"[常用字段:智能] OR "腹针"[常用字段:智能] OR "腕踝针"[常用字段:智能] OR "眼针"[常用字段:智能] OR "鼻针"[常用字段:智能] OR "唇针"[常用字段:智能] OR "耳针"[常用字段:智能] OR "浮针"[常用字段:智能] OR "揿针"[常用字段:智能] OR "温针"[常用字段:智能] OR "埋针"[常用字段:智能] OR "埋线"[常用字段:智能] OR "刺络"[常用字段:智能] OR "皮%针"[常用字段:智能] OR "梅花针"[常用字段:智能] OR "三棱针"[常用字段:智能] OR "激光针"[常用字段:智能] OR "艾灸"[常用字段:智能] OR "经皮%电刺激"[常用字段:智能] OR "推拿"[常用字段:智能] OR "按摩"[常用字段:智能] OR "拔罐"[常用字段:智能] OR "刮痧"[常用字段:智能] OR "埋豆"[常用字段:智能] OR "耳穴"[常用字段:智能] OR "药浴"[常用字段:智能] OR "足浴"[常用字段:智能] |
| #5 | "临床研究"[常用字段:智能] OR "临床试验"[常用字段:智能] OR "随机对照试验"[常用字段:智能] OR "对照试验"[常用字段:智能] OR "随机"[常用字段:智能] OR "疗效评价"[常用字段:智能] |
| #6 | (#4 AND #5) |
| #7 | #5 NOT ("动物"[常用字段:智能] OR "鼠"[常用字段:智能] OR "Meta分析"[常用字段:智能]) |
| #8 | (#3 AND #6 AND #7) |

**The search strategy for the Chinese Science and Technology Periodical (VIP) database**

The retrieval of the VIP database was conducted on June 28, 2025, and a total of 55 records were retrieved.

| **No** | **Search History** |
| --- | --- |
| #1 | M=("阿片类药物诱导便秘" OR "药物相关性便秘" OR "药物性便秘" OR "便秘") OR R=("阿片类药物诱导便秘" OR "药物相关性便秘" OR "药物性便秘" OR "便秘") |
| #2 | M=("镇痛药, 阿片类" OR "镇痛药" OR "阿片" OR "海洛因" OR "吗啡" OR "美施康定" OR "美菲康" OR "可待因" OR "氢可酮" OR "羟考酮" OR "羟吗啡酮" OR "氧吗啡酮" OR "芬太尼" OR "氢吗啡酮" OR "他喷他多" OR "美沙酮" OR "丁丙诺啡" OR "曲马多" OR "曲马朵" OR "维柯丁" OR "维可定" OR "奥施康定") OR R=("镇痛药, 阿片类" OR "镇痛药" OR "阿片" OR "海洛因" OR "吗啡" OR "美施康定" OR "美菲康" OR "可待因" OR "氢可酮" OR "羟考酮" OR "羟吗啡酮" OR "氧吗啡酮" OR "芬太尼" OR "氢吗啡酮" OR "他喷他多" OR "美沙酮" OR "丁丙诺啡" OR "曲马多" OR "曲马朵" OR "维柯丁" OR "维可定" OR "奥施康定") |
| #3 | M=("针灸疗法" OR "针刺" OR "电针" OR "针刺穴位" OR "耳针疗法" OR "耳穴贴压疗法" OR "灸法" OR "穴位按压" OR "经皮神经电刺激" OR "按摩" OR "拔罐疗法" OR "药熨疗法" OR "热敷疗法" OR "灸" OR "穴" OR "针灸" OR "穴位" OR "电针" OR "针刀" OR "火针" OR "毫针" OR "干针" OR "微针" OR "手针" OR "头针" OR "腹针" OR "腕踝针" OR "眼针" OR "鼻针" OR "唇针" OR "耳针" OR "浮针" OR "揿针" OR "温针" OR "埋针" OR "埋线" OR "刺络" OR "皮%针" OR "梅花针" OR "三棱针" OR "激光针" OR "艾灸" OR "经皮%电刺激" OR "推拿" OR "拔罐" OR "刮痧" OR "埋豆" OR "耳穴" OR "药浴" OR "足浴") OR R=("针灸疗法" OR "针刺" OR "电针" OR "针刺穴位" OR "耳针疗法" OR "耳穴贴压疗法" OR "灸法" OR "穴位按压" OR "经皮神经电刺激" OR "按摩" OR "拔罐疗法" OR "药熨疗法" OR "热敷疗法" OR "灸" OR "穴" OR "针灸" OR "穴位" OR "电针" OR "针刀" OR "火针" OR "毫针" OR "干针" OR "微针" OR "手针" OR "头针" OR "腹针" OR "腕踝针" OR "眼针" OR "鼻针" OR "唇针" OR "耳针" OR "浮针" OR "揿针" OR "温针" OR "埋针" OR "埋线" OR "刺络" OR "皮%针" OR "梅花针" OR "三棱针" OR "激光针" OR "艾灸" OR "经皮%电刺激" OR "推拿" OR "拔罐" OR "刮痧" OR "埋豆" OR "耳穴" OR "药浴" OR "足浴") |
| #4 | M=("临床研究" OR "临床试验" OR "随机对照试验" OR "对照试验" OR "随机" OR "疗效评价") OR R=("临床研究" OR "临床试验" OR "随机对照试验" OR "对照试验" OR "随机" OR "疗效评价") |
| #5 | M=("动物" OR "鼠" OR "Meta分析") OR R=("动物" OR "鼠" OR "Meta分析") |
| #6 | (#1 AND #2 AND #3 AND #4 NOT #5) |

**The search strategy for Wan Fang database**

The retrieval of the WanFang database was conducted on June 28, 2025, and a total of 18 records were retrieved.

| **No** | **Search History** |
| --- | --- |
| #1 | 题名或关键词:"阿片类药物诱导便秘" OR "药物相关性便秘" OR "药物性便秘" OR "便秘" |
| #2 | 题名或关键词:"镇痛药, 阿片类" OR "镇痛药" OR "阿片" OR "海洛因" OR "吗啡" OR "美施康定" OR "美菲康" OR "可待因" OR "氢可酮" OR "羟考酮" OR "羟吗啡酮" OR "氧吗啡酮" OR "芬太尼" OR "氢吗啡酮" OR "他喷他多" OR "美沙酮" OR "丁丙诺啡" OR "曲马多" OR "曲马朵" OR "维柯丁" OR "维可定" OR "奥施康定" |
| #3 | 题名或关键词: "针灸疗法" OR "针刺" OR "电针" OR "针刺穴位" OR "耳针疗法" OR "耳穴贴压疗法" OR "灸法" OR "穴位按压" OR "经皮神经电刺激" OR "按摩" OR "拔罐疗法" OR "药熨疗法" OR "热敷疗法" OR "灸" OR "穴" OR "针灸" OR "穴位" OR "电针" OR "针刀" OR "火针" OR "毫针" OR "干针" OR "微针" OR "手针" OR "头针" OR "腹针" OR "腕踝针" OR "眼针" OR "鼻针" OR "唇针" OR "耳针" OR "浮针" OR "揿针" OR "温针" OR "埋针" OR "埋线" OR "刺络" OR "皮*针" OR "梅花针" OR "三棱针" OR "激光针" OR "艾灸" OR "经皮*电刺激" OR "推拿" OR "拔罐" OR "刮痧" OR "埋豆" OR "耳穴" OR "药浴" OR "足浴" |
| #4 | 题名或关键词: "临床研究" OR "临床试验" OR "随机对照试验" OR "对照试验" OR "随机" OR "疗效评价" |
| #5 | 题名或关键词: "动物" OR "鼠" OR "Meta分析" |
| #6 | (#1 AND #2 AND #3 AND #4 NOT #5) |

**Supplement 2. Excluded studies after full-text review**

| **No.** | **Study** | **Reason for exclusion** |
| --- | --- | --- |
| **Non-cancer pain patients: 2** | | |
| 1 | 陈燕. 耳穴压豆预防吗啡类药物所致便秘的疗效观察. *中医临床研究*. 2015;7(25):122, 125. | The study population did not comprise patients with cancer. |
| 2 | 付倩. 经皮穴位电刺激治疗海洛因使用障碍者便秘的疗效观察. 硕士学位论文. 贵州中医药大学; 2021. Accessed March 2, 2025. | The study population did not comprise patients with cancer. |
| **Non-Acupuncture: 1** | | |
| 3 | 黄秀芳, 熊丽萍, 许彩云. 小郁子油按摩预防癌痛患者药源性便秘的疗效观察. *当代护士（中旬刊）*. 2017;(8):130-131. | The intervention did not meet the eligibility criteria for acupuncture-related therapies. |
| **Data of outcome indicators unavailable: 38** | | |
| 4 | 邓海燕, 陈信义, 郑丽平, et al. 自拟中药通便散敷脐治疗阿片类止痛药所致便秘的临床观察. *中国医刊*. 2013;48(12):96-98. | The prespecified constipation outcomes were not reported. |
| 5 | 邓海燕, 陈信义, 刘丹, et al. 中药穴位贴敷神阙穴治疗阿片类药物所致便秘的临床观察. *中国医刊*. 2016;51(7):110-112. | The prespecified constipation outcomes were not reported. |
| 6 | 丰银平, 陈灵勇, 何周华. 芒硝穴位贴敷治疗肺癌患者止痛药物所致便秘疗效观察. *中文科技期刊数据库（文摘版）医药卫生*. 2021;(8):61-62. | Only an overall clinical response rate was reported, without data for the prespecified constipation outcomes. |
| 7 | 丰银平, 刘忠达, 李权, et al. 芒硝神阙穴贴敷治疗吗啡所致便秘30例. *浙江中医杂志*. 2017;52(10):757. doi:10.13633/j.cnki.zjtcm.2017.10.038 | The prespecified constipation outcomes were not reported. |
| 8 | 丰银平, 刘忠达, 李权, et al. 黄芪芒硝神阙穴贴敷治疗芬太尼贴剂所致便秘30例. *浙江中医杂志*. 2019;54(6):420. doi:10.13633/j.cnki.zjtcm.2019.06.023 | The relevant outcome was reported, but group-specific numerical data were unavailable. |
| 9 | 关丽, 葛楠, 杨中, et al. 中药穴位贴敷治疗肿瘤患者奥施康定相关性便秘的效果. *中国医药导报*. 2020;17(2):144-147. doi:10.20047/j.issn1673-7210.2020.02.036 | The relevant outcome was reported, but no extractable post-treatment numerical data were available. |
| 10 | 李丽, 苏琴, 高婷, et al. 中药穴位敷贴联合乳果糖治疗阿片类药物相关性便秘效果观察. *临床合理用药杂志*. 2020;13(10):63-64. doi:10.15887/j.cnki.13-1389/r.2020.10.036 | The prespecified constipation outcomes were not reported. |
| 11 | 李松伟. 揿针防治阿片类相关胃肠道反应的临床效果观察. 硕士学位论文. 安徽中医药大学; 2023. doi:10.26922/d.cnki.ganzc.2023.000426 | The relevant outcome was reported, but group-specific numerical data were unavailable. |
| 12 | 廖惠莲. 小郁子油子午流注穴位贴敷联合中医护理在预防奥施康定致便秘中的应用. *护理研究*. 2016;30(11):1333-1336. | The prespecified constipation outcomes were not reported. |
| 13 | 廖惠莲, 傅秀芳, 梁葵心, et al. 小郁子油子午流注穴位敷贴干预奥施康定所致便秘100例. *中医研究*. 2015;28(7):44-45. | The prespecified constipation outcomes were not reported. |
| 14 | 刘田田. 穴位埋线防治阿片类药物所致便秘的临床观察. 硕士学位论文. 安徽中医药大学; 2017. Accessed March 2, 2025. | The prespecified constipation outcomes were not reported. |
| 15 | 鲁叶云, 徐振晔, 邓海滨, et al. 大黄穴位贴敷治疗恶性肿瘤患者阿片类药物相关性便秘的临床观察. *结直肠肛门外科*. 2020;26(S01):18-19. | Only an overall clinical response rate was reported, without data for the prespecified constipation outcomes. |
| 16 | 容景瑜, 林清, 陈洁, et al. 基于子午流注纳子法予四子散外敷治疗阿片类药物所致便秘的临床研究. *中国实用医药*. 2018;13(4):79-81. doi:10.14163/j.cnki.11-5547/r.2018.04.048 | The relevant outcome was reported, but no extractable post-treatment numerical data were available. |
| 17 | 孙瑞瑞, 曾永蕾, 王茎, et al. 针刺治疗盐酸羟考酮缓释片所致便秘疗效观察. *上海针灸杂志*. 2016;35(7):827-829. doi:10.13460/j.issn.1005-0957.2016.07.0827 | The prespecified constipation outcomes were not reported. |
| 18 | 王文雯. 针灸治疗阿片类药物相关便秘临床观察. 硕士学位论文. 北京中医药大学; 2011. Accessed March 2, 2025. | The relevant outcome was reported, but no extractable post-treatment numerical data were available. |
| 19 | 魏莹, 韩丽君, 马媛, et al. 穴位贴敷联合中医护理治疗阿片类药物相关性便秘的临床观察. *世界最新医学信息文摘（连续型电子期刊）*. 2020;20(A1):305-306. doi:10.3969/j.issn.1671-3141.2020.101.152 | The prespecified constipation outcomes were not reported. |
| 20 | 吴如雷, 戴小桃, 陈翔. 通便散贴敷神阙穴辅治阿片类药物所致便秘临床研究. *实用中医药杂志*. 2018;34(10):1264. | The prespecified constipation outcomes were not reported. |
| 21 | 吴银华. 行气通便贴预防口服吗啡所致便秘中的应用. *国际护理学杂志*. 2012;31(11):2165-2166. | The relevant continuous outcome was reported without an SD or other measure of variability, and the required data could not be derived. |
| 22 | 夏超. 提肛运动配合中药穴位贴敷治疗阿片类药物所致便秘的疗效观察. *全科护理*. 2015;13(35):3598-3599. | The prespecified constipation outcomes were not reported. |
| 23 | 严妍, 程园园, 任晓琴. 艾灸对癌痛患者阿片类药物镇痛致便秘的疗效观察. *中医药临床杂志*. 2014;26(8):785-786. doi:10.16448/j.cjtcm.2014.08.007 | The prespecified constipation outcomes were not reported. |
| 24 | 晏晴艳. 穴位按压对癌痛患者阿片类药物镇痛致便秘的疗效观察. *西南军医*. 2017;19(6):577-578. | The prespecified constipation outcomes were not reported. |
| 25 | 杨利聪. 便秘方穴位贴敷治疗羟考酮缓释片所致便秘32例. *中医外治杂志*. 2015;24(3):21-22. | The prespecified constipation outcomes were not reported. |
| 26 | 周钰, 常湘涛, 张政军. 针灸防治阿片类镇痛药便秘不良反应的临床观察. *辽宁中医杂志*. 2010;37(2):338-339. doi:10.13192/j.ljtcm.2010.02.151.zhouy.004 | Only an overall clinical response rate was reported, without data for the prespecified constipation outcomes. |
| 27 | 朱俏婷, 丰银平. 耳穴压豆对吗啡引发便秘患者胃肠激素的影响及疗效观察. *中国现代医生*. 2019;57(2):74-76, 80. | Only an overall clinical response rate was reported, without data for the prespecified constipation outcomes. |
| 28 | 马亮, 张莹, 曾保霞, et al. 中药穴位贴敷神阙治疗晚期肺癌应用阿片类药物致便秘41例. *陕西中医药大学学报*. 2022;45(2):93-96. doi:10.13424/j.cnki.jsctcm.2022.02.021 | Only an overall clinical response rate was reported, without data for the prespecified constipation outcomes. |
| 29 | 陈滨芬. 乳果糖加穴位按摩治疗阿片类药物相关性便秘49例. *福建中医药*. 2022;53(12):60-62. doi:10.13260/j.cnki.jfjtcm.012609 | Only an overall clinical response rate was reported, without data for the prespecified constipation outcomes. |
| 30 | 蒋益兰, 王其美. 针刺治疗肿瘤服阿片类镇痛药致便秘患者30例疗效观察. *中国中医药科技*. 2010;17(6):544-545. doi:10.3969/j.issn.1005-7072.2010.06.059 | The prespecified constipation outcomes were not reported. |
| 31 | 孙红, 方莉亚, 卜亚美, et al. 沟通达标教育结合穴位按摩在阿片类药物性便秘患者中的应用. *中华全科医学*. 2024;22(9):1580-1583. doi:10.16766/j.cnki.issn.1674-4152.003687 | Only an overall clinical response rate was reported, without data for the prespecified constipation outcomes. |
| 32 | 王文雯, 李仝, 宋凤丽. 针刺治疗阿片类药物相关便秘临床观察. *中日友好医院学报*. 2017;31(1):18-21. | The relevant outcome was reported, but no extractable post-treatment numerical data were available. |
| 33 | 魏世民, 董宝强, 富昱, et al. 经筋刺法治疗肺癌癌性疼痛患者药源性便秘的临床研究. 辽*宁中医杂志*. 2019;46(9):1952-1954. doi:10.13192/j.issn.1000-1719.2019.09.049 | The prespecified constipation outcomes were not reported. |
| 34 | 杨静怡, 夏黎明. 俞募配穴针刺治疗肿瘤患者口服羟考酮所致便秘疗效观察. *中医药临床杂志*. 2022;34(4):767-770. doi:10.16448/j.cjtcm.2022.0440 | The prespecified constipation outcomes were not reported. |
| 35 | 赵晔, 龚欣, 王其美, et al. 针刺治疗肿瘤患者服用阿片类镇痛药所致便秘150例总结. *湖南中医杂志*. 2021;37(1):65-67. doi:10.16808/j.cnki.issn1003-7705.2021.01.023 | The prespecified constipation outcomes were not reported. |
| 36 | 张翔, 顾芳红, 张建林, et al. 耳穴贴压联合杜秘克预防阿片性便秘33例疗效观察. *云南中医中药杂志*. 2017;38(9):47-48. doi:10.16254/j.cnki.53-1120/r.2017.09.022 | The relevant outcome was reported, but group-specific numerical data were unavailable. |
| 37 | 张巧艺. 自拟中药方膏摩预防癌痛患者服用阿片类药物所致的便秘的临床观察. 硕士学位论文. 福建中医药大学; 2021. doi:10.27021/d.cnki.gfjzc.2021.000144 | The prespecified constipation outcomes were not reported. |
| 38 | 赵江花, 张国清, 才向华. 四磨汤组方穴位热敷防治麻醉品所致癌症患者便秘的疗效观察. *中国中医药科技*. 2012;19(4):341. | The relevant outcome was reported, but no extractable post-treatment numerical data were available. |
| 39 | 梁攀. 加味增液承气脐贴治疗阿片类药物相关性便秘阴虚肠燥证的临床观察. 硕士学位论文. 湖南中医药大学; 2021. doi:10.27138/d.cnki.ghuzc.2021.000050 | The relevant outcome was reported, but no extractable post-treatment numerical data were available. |
| 40 | 刘玉. 中药穴位贴敷联合延续性护理对癌性疼痛患者镇痛及便秘的影响. *中国伤残医学*. 2016;24(3):140-141. | The prespecified constipation outcomes were not reported. |
| 41 | 姚莉莉, 张琦, 李琦. 神阙穴贴敷对气虚型阿片类药物相关性便秘患者的临床疗效. *中国医药科学*. 2023;13(17):136-139, 178. doi:10.20116/j.issn2095-0616.2023.17.32 | The prespecified constipation outcomes were not reported. |
| **Non-standard laxative: 5** | | |
| 42 | 罗璐. 一捻金穴位贴敷治疗阿片类镇痛药物所致便秘的临床效果. *河南医学研究*. 2023;32(2):337-340. | The comparator did not meet the prespecified eligibility criteria because it consisted of a Chinese herbal medicine rather than an eligible conventional laxative. |
| 43 | 汪亚男, 候大妮, 孔凡铭, et al. 三焦灸技术在癌痛患者阿片类药物相关性便秘中的应用研究. 天*津中医药*. 2024;41(5):612-616. | The comparator did not meet the prespecified eligibility criteria because it consisted of a Chinese herbal medicine rather than an eligible conventional laxative. |
| 44 | 赵芳, 倪小青. 针刺治疗阿片类药物相关性便秘的疗效观察. *中医临床研究*. 2021;13(21):92-94. | The comparator did not meet the prespecified eligibility criteria because mosapride, a gastrointestinal prokinetic agent, was used instead of an eligible conventional laxative. |
| 45 | 张锋利, 林洪生, 何庆勇. 电针治疗肿瘤患者口服硫酸吗啡控释片所致便秘的临床研究. *中国中西医结合杂志*. 2009;29(10):922-925. | The comparator did not meet the prespecified eligibility criteria because mosapride, a gastrointestinal prokinetic agent, was used instead of an eligible conventional laxative. |
| 46 | 邹燕, 刘建阳. 揿针疗法干预阿片类药物所致顽固性便秘疗效观察. *上海针灸杂志*. 2019;38(6):638-641. doi:10.13460/j.issn.1005-0957.2019.06.0638 | The comparator did not meet the prespecified eligibility criteria because it consisted of a Chinese herbal medicine rather than an eligible conventional laxative. |

**Supplement 3. Pairwise meta-analysis and single-study direct comparisons**

**Supplement 3A. CCS**

| Comparison | Number | Intervention/Control  (n) | MD (95%) | *I*^2^ | *P* |
| --- | --- | --- | --- | --- | --- |
| TEAS VS OSM | 1 | 58/60 | -0.38 [-1.03; 0.27] | / | 0.2517 |
| EA VS OSM | 1 | 50/50 | -6.34 [-7.13; -5.55] | / | < 0.0001 |
| APA VS OSM | 4 | 151/149 | -0.75 [-4.38; 2.88] | 97% | 0.6857 |
| PN VS OSM | 1 | 36/36 | -2.00 [-2.85; -1.15] | / | < 0.0001 |
| MA VS OSM | 1 | 58/59 | -1.00 [-1.43; -0.57] | / | < 0.0001 |
| APA+OSM VS OSM | 2 | 97/90 | -1.75 [-7.08; 3.58] | 96% | 0.5205 |
| PN+OSM VS OSM | 1 | 50/50 | -3.27 [-3.99; -2.55] | / | < 0.0001 |
| MA+OSM VS OSM | 1 | 30/30 | -4.33 [-6.50; -2.16] | / | < 0.0001 |
| APA VS STI | 1 | 22/22 | 0.01 [-0.59; 0.61] | / | 0.9741 |
| MOX VS STI | 1 | 35/35 | -1.42 [-2.13; -0.71] | / | < 0.0001 |

Abbreviations: CCS, Cleveland Constipation Scale; OSM, osmotic laxatives; STI, stimulant laxatives; EA, electroacupuncture; MOX, moxibustion; PN, press needle; APA, acupoint application; MA, manual acupuncture; TEAS, transcutaneous electrical acupoint stimulation.

**Supplement 3B. Weekly SBMs**

| Comparison | Number | Intervention/Control  (n) | MD (95%) | *I*^2^ | *P* |
| --- | --- | --- | --- | --- | --- |
| EA VS OSM | 1 | 45/45 | 0.46 [0.09; 0.83] | / | 0.0142 |
| APA VS OSM | 1 | 22/20 | 0.40 [0.03; 0.77] | / | 0.0330 |
| MOX +OSM VS OSM | 1 | 42/40 | 1.30 [0.93; 1.67] | / | < 0.0001 |
| EA VS SA | 1 | 47/45 | 0.70 [0.43; 0.97] | / | < 0.0001 |
| APA VS STI | 1 | 76/76 | 1.67 [1.32; 2.02] | / | < 0.0001 |

Abbreviations: SBMs, spontaneous bowel movements per week; OSM, osmotic laxatives; STI, stimulant laxatives; MOX, moxibustion; EA, electroacupuncture; APA, acupoint application; SA, sham acupuncture.

**Supplement 3C. BFI**

| Comparison | Number | Intervention/Control  (n) | MD (95%) | *I*^2^ | *P* |
| --- | --- | --- | --- | --- | --- |
| AA+OSM VS OSM | 1 | 30/30 | -27.66 [-33.90; -21.42] | / | < 0.0001 |
| APA+OSM VS OSM | 1 | 31/31 | -10.58 [-13.35; -7.81] | / | < 0.0001 |
| AP+OSM VS OSM | 1 | 50/50 | -13.40 [-15.63; -11.17] | / | < 0.0001 |

Abbreviations: BFI, Bowel Function Index; OSM, osmotic laxatives; AA, auricular acupuncture; AP, acupressure; APA, acupoint application.

**Supplement 3D. BFI: Feeling of incomplete bowel evacuation (1)**

| Comparison | Number | Intervention/Control  (n) | MD (95%) | *I*^2^ | *P* |
| --- | --- | --- | --- | --- | --- |
| AA+OSM VS OSM | 1 | 30/30 | -29.00 [-35.78; -22.22] | / | < 0.0001 |

Abbreviations: BFI, Bowel Function Index; OSM, osmotic laxatives; AA, auricular acupuncture; APA, acupoint application.

**Supplement 3E. BFI: Feeling of incomplete bowel evacuation (2)**

| Comparison | Number | Intervention/Control  (n) | MD (95%) | *I*^2^ | *P* |
| --- | --- | --- | --- | --- | --- |
| APA VS BC | 2 | 75/75 | -8.10 [-17.86; 1.66] | 82% | 0.1038 |
| APA VS SA | 1 | 40/40 | -6.36 [-8.72; -4.00] | / | < 0.0001 |

Abbreviations: BFI, Bowel Function Index; BC, blank control; APA, acupoint application; SA, sham acupuncture.

**Supplement 3F. BFI: Ease of defecation (1)**

| Comparison | Number | Intervention/Control  (n) | MD (95%) | *I*^2^ | *P* |
| --- | --- | --- | --- | --- | --- |
| AA+OSM VS OSM | 1 | 30/30 | -32.07 [-39.41; -24.73] | / | < 0.0001 |

Abbreviations: BFI, Bowel Function Index; OSM, osmotic laxatives; AA, auricular acupuncture; APA, acupoint application.

**Supplement 3G. BFI: Ease of defecation (2)**

| Comparison | Number | Intervention/Control  (n) | MD (95%) | *I*^2^ | *P* |
| --- | --- | --- | --- | --- | --- |
| APA VS BC | 2 | 75/75 | -7.39 [-10.49; -4.30] | 46% | < 0.0001 |
| APA VS SA | 1 | 40/40 | -5.51 [-7.56; -3.46] | / | < 0.0001 |

Abbreviations: BFI, Bowel Function Index; BC, blank control; APA, acupoint application; SA, sham acupuncture.

**Supplement 3H. BFI: Personal judgement of constipation (1)**

| Comparison | Number | Intervention/Control  (n) | MD (95%) | *I*^2^ | *P* |
| --- | --- | --- | --- | --- | --- |
| AA+OSM VS OSM | 1 | 30/30 | -22.17 [-28.93; -15.41] | / | < 0.0001 |

Abbreviations: BFI, Bowel Function Index; OSM, osmotic laxatives; AA, auricular acupuncture; APA, acupoint application.

**Supplement 3I. BFI**: **Personal judgement of constipation (2)**

| Comparison | Number | Intervention/Control  (n) | MD (95%) | *I*^2^ | *P* |
| --- | --- | --- | --- | --- | --- |
| APA VS BC | 2 | 75/75 | -9.20 [-12.26; -6.14] | 0% | < 0.0001 |
| APA VS SA | 1 | 40/40 | -4.74 [-6.87; -2.61] | / | < 0.0001 |

Abbreviations: BFI, Bowel Function Index; BC, blank control; APA, acupoint application; SA, sham acupuncture.

**Supplement 3J. PAC-SYM**

| Comparison | Number | Intervention/Control  (n) | MD (95%) | *I*^2^ | *P* |
| --- | --- | --- | --- | --- | --- |
| APA VS OSM | 1 | 54/52 | -0.40 [-0.58; -0.22] | / | < 0.0001 |
| APA VS BC | 2 | 80/80 | -0.62 [-0.89; -0.34] | 87% | < 0.0001 |

Abbreviations: PAC-SYM, Patient Assessment of Constipation–Symptoms; OSM, osmotic laxatives; BC, blank control; APA, acupoint application.

**Supplement 3K. BSFS**

| Comparison | Number | Intervention/Control  (n) | MD (95%) | *I*^2^ | *P* |
| --- | --- | --- | --- | --- | --- |
| EA VS OSM | 1 | 22/20 | 0.17 [-0.63; 0.97] | / | 0.6786 |
| MOX VS OSM | 1 | 33/33 | 1.19 [0.42; 1.96] | / | 0.0024 |
| APA VS OSM | 2 | 67/66 | -0.30 [-0.55; -0.04] | 47% | 0.0228 |
| AA+OSM VS OSM | 1 | 30/30 | 0.94 [-0.22; 2.10] | / | 0.1136 |
| PN+OSM VS OSM | 2 | 83/83 | 1.06 [0.73; 1.40] | 0% | <0.0001 |
| APA+OSM VS OSM | 3 | 130/129 | 0.07 [-1.24; 1.39] | 99% | 0.9120 |
| APA VS BC | 1 | 45/45 | 0.42 [0.19; 0.65] | / | 0.0003 |
| AP VS BC | 1 | 44/44 | 1.82 [0.82; 2.82] | / | 0.0004 |
| EA VS SA | 1 | 47/45 | 0.20 [-0.06; 0.46] | / | 0.1323 |

Abbreviations: BSFS, Bristol Stool Form Scale; OSM, osmotic laxatives; BC, blank control; AP, acupressure; MOX, moxibustion; PN, press needle; AA, auricular acupuncture; EA, electroacupuncture; APA, acupoint application; SA, sham acupuncture.

**Supplement 3L. PAC-QOL**

| Comparison | Number | Intervention/Control  (n) | MD (95%) | *I*^2^ | *P* |
| --- | --- | --- | --- | --- | --- |
| TEAS VS OSM | 1 | 58/60 | -0.05 [-0.13; 0.03] | / | 0.2066 |
| PN VS OSM | 1 | 36/36 | -0.23 [-0.37; -0.09] | / | 0.0013 |
| MA VS OSM | 1 | 33/32 | -0.04 [-0.14; 0.06] | / | 0.4298 |
| APA VS OSM | 3 | 119/118 | -0.15 [-0.85; 0.54] | 97% | 0.6673 |
| APA+OSM VS OSM | 1 | 68/61 | -0.14 [-0.27; -0.01] | / | 0.0301 |
| MOX+OSM VS OSM | 1 | 42/40 | -0.49 [-0.61; -0.37] | / | < 0.0001 |
| AA+OSM VS OSM | 1 | 30/30 | -0.59 [-0.68; -0.50] | / | < 0.0001 |
| PN+OSM VS OSM | 1 | 50/50 | -0.27 [-0.33; -0.21] | / | < 0.0001 |

Abbreviations: PAC-QOL, Patient Assessment of Constipation Quality of Life questionnaire; MOX, moxibustion; PN, press needle; AA, auricular acupuncture; APA, acupoint application; MA, manual acupuncture; TEAS, transcutaneous electrical acupoint stimulation.

**Supplement 3M. PAC-QOL: Worries and concerns**

| Comparison | Number | Intervention/Control  (n) | MD (95%) | *I*^2^ | *P* |
| --- | --- | --- | --- | --- | --- |
| AP VS BC | 1 | 70/70 | -0.84 [-1.01; -0.67] | / | < 0.0001 |
| APA VS BC | 2 | 80/80 | -0.65 [-0.71; -0.58] | 0% | < 0.0001 |
| APA VS SA | 1 | 40/40 | -0.49 [-0.83; -0.15] | / | 0.0047 |

Abbreviations: PAC-QOL, Patient Assessment of Constipation Quality of Life questionnaire; BC, blank control; APA, acupoint application; SA, sham acupuncture; AP, acupressure.

**Supplement 3N. PAC-QOL: Physical**

| Comparison | Number | Intervention/Control  (n) | MD (95%) | *I*^2^ | *P* |
| --- | --- | --- | --- | --- | --- |
| AP VS BC | 1 | 70/70 | -0.95 [-1.12; -0.78] | / | < 0.0001 |
| APA VS BC | 2 | 80/80 | -0.68 [-0.79; -0.56] | 0% | < 0.0001 |
| APA VS SA | 1 | 40/40 | -0.58 [-0.93; -0.23] | / | 0.0011 |

Abbreviations: PAC-QOL, Patient Assessment of Constipation Quality of Life questionnaire; BC, blank control; APA, acupoint application; AP, acupressure; SA, sham acupuncture.

**Supplement 3O. PAC-QOL: Satisfaction**

| Comparison | Number | Intervention/Control  (n) | MD (95%) | *I*^2^ | *P* |
| --- | --- | --- | --- | --- | --- |
| AP VS BC | 1 | 70/70 | -0.22 [-0.42; -0.02] | / | 0.0289 |
| APA VS BC | 2 | 80/80 | -0.63 [-0.73; -0.53] | 38% | < 0.0001 |
| APA VS SA | 1 | 40/40 | -0.37 [-0.62; -0.12] | / | 0.0044 |

Abbreviations: PAC-QOL, Patient Assessment of Constipation Quality of Life questionnaire; BC, blank control; APA, acupoint application; SA, sham acupuncture; AP, acupressure.

**Supplement 3P. PAC-QOL: Psychosocial**

| Comparison | Number | Intervention/Control  (n) | MD (95%) | *I*^2^ | *P* |
| --- | --- | --- | --- | --- | --- |
| AP VS BC | 1 | 70/70 | -1.15 [-1.33; -0.97] | / | < 0.0001 |
| APA VS BC | 2 | 80/80 | -0.64 [-0.73; -0.54] | 0% | < 0.0001 |
| APA VS SA | 1 | 40/40 | -0.49 [-0.81; -0.17] | / | 0.0029 |

Abbreviations: PAC-QOL, Patient Assessment of Constipation Quality of Life questionnaire; BC, blank control; APA, acupoint application; SA, sham acupuncture; AP, acupressure.

**Supplement 4. League Table for Network Meta-Analysis**

**Supplement 4A. BFI: Feeling of incomplete bowel evacuation (1)**

| AA+OSM |  |  |
| --- | --- | --- |
| -15.36 (-66.04, 36.15) | APA+OSM |  |
| -29.10 (-65.52, 7.58) | -13.69 (-50.26, 22.36) | OSM |

The results are presented as mean difference (MDs) with 95% credible intervals (CrIs). The comparison must be read from left to right. For this BFI outcome, values less than zero indicate that the treatment on the left is favored. Values in bold text indicate statistical significance.

Abbreviations: BFI, Bowel Function Index; OSM, osmotic laxatives; AA, auricular acupuncture; APA, acupoint application.

**Supplement 4B. BFI: Feeling of incomplete bowel evacuation (2)**

| APA |  |  |
| --- | --- | --- |
| -6.35 (-22.69, 9.87) | SA | - |
| -8.51 (-19.85, 4.23) | -2.31 (-21.63, 18.92) | BC |

The results are presented as mean difference (MDs) with 95% credible intervals (CrIs). The comparison must be read from left to right. For this BFI outcome, values less than zero indicate that the treatment on the left is favored. Values in bold text indicate statistical significance.

Abbreviations: BFI, Bowel Function Index; BC, blank control; APA, acupoint application; SA, sham acupuncture.

**Supplement 4C. BFI: Ease of defecation (2)**

| APA |  |  |
| --- | --- | --- |
| -5.52 (-15.71, 4.44) | SA |  |
| -6.36 (-13.40, 2.79) | -0.95 (-12.54, 13.55) | BC |

The results are presented as mean difference (MDs) with 95% credible intervals (CrIs). The comparison must be read from left to right. For this BFI outcome, values less than zero indicate that the treatment on the left is favored. Values in bold text indicate statistical significance.

Abbreviations: BFI, Bowel Function Index; BC, blank control; APA, acupoint application; SA, sham acupuncture.

**Supplement 4D. BFI: Personal judgement of constipation (1)**

| AA+OSM |  |  |
| --- | --- | --- |
| -8.72 (-48.46, 30.29) | APA+OSM |  |
| -22.13 (-50.49, 5.84) | -13.33 (-41.84, 14.46) | OSM |

The results are presented as mean difference (MDs) with 95% credible intervals (CrIs). The comparison must be read from left to right. For this BFI outcome, values less than zero indicate that the treatment on the left is favored. Values in bold text indicate statistical significance.

Abbreviations: BFI, Bowel Function Index; OSM, osmotic laxatives; AA, auricular acupuncture; APA, acupoint application.

**Supplement 4E. BFI: Personal judgement of constipation (2)**

| APA |  |  |
| --- | --- | --- |
| -4.77 (-15.13, 5.29) | SA | - |
| **-9.07 (-16.83, -0.82)** | -4.39 (-17.06, 9.00) | BC |

The results are presented as mean difference (MDs) with 95% credible intervals (CrIs). The comparison must be read from left to right. For this BFI outcome, values less than zero indicate that the treatment on the left is favored. Values in bold text indicate statistical significance.

Abbreviations: BFI, Bowel Function Index; BC, blank control; APA, acupoint application; SA, sham acupuncture.

**Supplement 4F. PAC-QOL**

| AA+OSM |  |  |  |  |  |  |  |  |
| --- | --- | --- | --- | --- | --- | --- | --- | --- |
| -0.11 (-1.56, 1.35) | MOX+OSM |  |  |  |  |  |  |  |
| -0.32 (-1.76, 1.14) | -0.22 (-1.69, 1.22) | PN+OSM |  |  |  |  |  |  |
| -0.36 (-1.84, 1.07) | -0.26 (-1.74, 1.21) | -0.05 (-1.50, 1.41) | PN |  |  |  |  |  |
| -0.43 (-1.63, 0.75) | -0.32 (-1.54, 0.87) | -0.11 (-1.32, 1.08) | -0.06 (-1.27, 1.14) | APA |  |  |  |  |
| -0.44 (-1.91, 1.00) | -0.34 (-1.79, 1.10) | -0.12 (-1.60, 1.34) | -0.08 (-1.54, 1.39) | -0.02 (-1.19, 1.18) | APA+OSM |  |  |  |
| -0.54 (-1.98, 0.89) | -0.44 (-1.89, 1.01) | -0.23 (-1.68, 1.23) | -0.18 (-1.64, 1.29) | -0.12 (-1.31, 1.08) | -0.10 (-1.55, 1.36) | TEAS |  |  |
| -0.55 (-2.02, 0.91) | -0.44 (-1.91, 1.00) | -0.23 (-1.71, 1.22) | -0.19 (-1.65, 1.28) | -0.12 (-1.32, 1.07) | -0.11 (-1.56, 1.36) | 0.00 (-1.46, 1.43) | MA |  |
| -0.59 (-1.62, 0.43) | -0.49 (-1.53, 0.55) | -0.27 (-1.31, 0.75) | -0.23 (-1.27, 0.81) | -0.16 (-0.76, 0.44) | -0.14 (-1.18, 0.88) | -0.05 (-1.07, 0.97) | -0.04 (-1.07, 1.00) | OSM |

The results are presented as mean difference (MDs) with 95% credible intervals (CrIs). The comparison must be read from left to right. For this PAC-QOL outcome, values less than zero indicate that the treatment on the left is favored. Values in bold text indicate statistical significance.

Abbreviations: PAC-QOL, Patient Assessment of Constipation Quality of Life questionnaire; MOX, moxibustion; PN, press needle; AA, auricular acupuncture; APA, acupoint application; MA, manual acupuncture; TEAS, transcutaneous electrical acupoint stimulation.

**Supplement 4G. PAC-QOL: Worries and concerns**

| AP |  |  |  |
| --- | --- | --- | --- |
| -0.22 (-1.30, 0.81) | APA |  |  |
| -0.71 (-2.13, 0.63) | -0.49 (-1.37, 0.38) | SA |  |
| -0.84 (-1.68, 0.03) | -0.62 (-1.20, 0.02) | -0.13 (-1.14, 0.97) | BC |

The results are presented as mean difference (MDs) with 95% credible intervals (CrIs). The comparison must be read from left to right. For this PAC-QOL outcome, values less than zero indicate that the treatment on the left is favored. Values in bold text indicate statistical significance.

Abbreviations: PAC-QOL, Patient Assessment of Constipation Quality of Life questionnaire; BC, blank control; APA, acupoint application; SA, sham acupuncture; AP, acupressure.

**Supplement 4H. PAC-QOL: Physical**

| AP |  |  |  |
| --- | --- | --- | --- |
| -0.27 (-1.36, 0.83) | APA |  |  |
| -0.84 (-2.29, 0.62) | -0.57 (-1.51, 0.35) | SA |  |
| **-0.95 (-1.84, -0.04)** | **-0.68 (-1.33, -0.04)** | –0.11 (-1.26, 1.02) | BC |

The results are presented as mean difference (MDs) with 95% credible intervals (CrIs). The comparison must be read from left to right. For this PAC-QOL outcome, values less than zero indicate that the treatment on the left is favored. Values in bold text indicate statistical significance.

Abbreviations: PAC-QOL, Patient Assessment of Constipation Quality of Life questionnaire; BC, blank control; APA, acupoint application; AP, acupressure; SA, sham acupuncture.

**Supplement 4I. PAC-QOL: Satisfaction**

| APA |  |  |  |
| --- | --- | --- | --- |
| -0.36 (-1.23, 0.58) | SA |  |  |
| -0.38 (-1.12, 0.37) | -0.02 (-1.23, 1.13) | AP |  |
| **-0.58 (-1.06, -0.01)** | -0.22 (-0.94, 0.53) | -0.21 (-1.08, 0.77) | BC |

The results are presented as mean difference (MDs) with 95% credible intervals (CrIs). The comparison must be read from left to right. For this PAC-QOL outcome, values less than zero indicate that the treatment on the left is favored. Values in bold text indicate statistical significance.

Abbreviations: PAC-QOL, Patient Assessment of Constipation Quality of Life questionnaire; BC, blank control; APA, acupoint application; SA, sham acupuncture; AP, acupressure.

**Supplement 4J. PAC-QOL: Psychosocial**

| AP |  |  |  |
| --- | --- | --- | --- |
| -0.53 (-1.91, 0.83) | APA |  |  |
| -1.02 (-2.80, 0.77) | -0.49 (-1.63, 0.63) | SA |  |
| **-1.16 (-2.25, -0.03)** | -0.63 (-1.40, 0.18) | -0.13 (-1.49, 1.24) | BC |

The results are presented as mean difference (MDs) with 95% credible intervals (CrIs). The comparison must be read from left to right. For this PAC-QOL outcome, values less than zero indicate that the treatment on the left is favored. Values in bold text indicate statistical significance.

Abbreviations: PAC-QOL, Patient Assessment of Constipation Quality of Life questionnaire; BC, blank control; APA, acupoint application; SA, sham acupuncture; AP, acupressure.

**Supplement 5. Certainty of evidence (GRADE)**

**Supplement 5A. CCS**

| Comparison | | Direct evidence | Network meta-analysis evidence |
| --- | --- | --- | --- |
| EA | MA+OSM | - | Very Low  Risk of bias (-1)  Imprecision (-1)  Intransitivity (-1) |
| EA | MOX | - | Very Low  Risk of bias (-1)  Inconsistency (-1)  Imprecision (-1)  Intransitivity (-1) |
| EA | PN+OSM | - | Very Low  Risk of bias (-1)  Imprecision (-1)  Intransitivity (-1) |
| EA | APA | - | Very Low  Risk of bias (-1)  Inconsistency (-1)  Imprecision (-1)  Intransitivity (-1) |
| EA | STI | - | Very Low  Risk of bias (-1)  Inconsistency (-1)  Imprecision (-1)  Intransitivity (-1) |
| EA | PN | - | Very Low  Risk of bias (-1)  Imprecision (-1)  Intransitivity (-1) |
| EA | APA+OSM | - | Very Low  Risk of bias (-1)  Inconsistency (-1)  Imprecision (-1)  Intransitivity (-1) |
| EA | MA | - | Very Low  Risk of bias (-1)  Imprecision (-1)  Intransitivity (-1) |
| EA | TEAS | - | Very Low  Risk of bias (-1)  Imprecision (-1)  Intransitivity (-1) |
| EA | OSM | Low  Risk of bias (-1)  Imprecision (-1) | Low  Risk of bias (-1)  Imprecision (-1) |
| MA+OSM | MOX | - | Very Low  Risk of bias (-1)  Inconsistency (-1)  Imprecision (-1)  Intransitivity (-1) |
| MA+OSM | PN+OSM | - | Low  Risk of bias (-1)  Imprecision (-1) |
| MA+OSM | APA | - | Very Low  Risk of bias (-1)  Inconsistency (-1)  Imprecision (-1)  Intransitivity (-1) |
| MA+OSM | STI | - | Very Low  Risk of bias (-1)  Inconsistency (-1)  Imprecision (-1)  Intransitivity (-1) |
| MA+OSM | PN | - | Very Low  Risk of bias (-1)  Imprecision (-1)  Intransitivity (-1) |
| MA+OSM | APA+OSM | - | Very Low  Risk of bias (-1)  Inconsistency (-1)  Imprecision (-1) |
| MA+OSM | MA | - | Very Low  Risk of bias (-1)  Imprecision (-1)  Intransitivity (-1) |
| MA+OSM | TEAS | - | Low  Risk of bias (-1)  Imprecision (-1) |
| MA+OSM | OSM | Low  Risk of bias (-1)  Imprecision (-1) | Low  Risk of bias (-1)  Imprecision (-1) |
| MOX | PN+OSM | - | Very Low  Risk of bias (-1)  Inconsistency (-1)  Imprecision (-1)  Intransitivity (-1) |
| MOX | APA | - | Very Low  Risk of bias (-1)  Imprecision (-1)  Intransitivity (-1) |
| MOX | STI | Low  Risk of bias (-1)  Imprecision (-1) | Low  Risk of bias (-1)  Imprecision (-1) |
| MOX | PN | - | Very Low  Risk of bias (-1)  Inconsistency (-1)  Imprecision (-1)  Intransitivity (-1) |
| MOX | APA+OSM | - | Very Low  Risk of bias (-1)  Inconsistency (-1)  Imprecision (-1)  Intransitivity (-1) |
| MOX | MA | - | Very Low  Risk of bias (-1)  Inconsistency (-1)  Imprecision (-1)  Intransitivity (-1) |
| MOX | TEAS | - | Very Low  Risk of bias (-1)  Inconsistency (-1)  Imprecision (-1)  Intransitivity (-1) |
| MOX | OSM | - | Very Low  Risk of bias (-1)  Inconsistency (-1)  Imprecision (-1)  Intransitivity (-1) |
| PN+OSM | APA | - | Very Low  Risk of bias (-1)  Inconsistency (-1)  Imprecision (-1)  Intransitivity (-1) |
| PN+OSM | STI | - | Very Low  Risk of bias (-1)  Inconsistency (-1)  Imprecision (-1)  Intransitivity (-1) |
| PN+OSM | PN | - | Very Low  Risk of bias (-1)  Imprecision (-1)  Intransitivity (-1) |
| PN+OSM | APA+OSM | - | Very Low  Risk of bias (-1)  Inconsistency (-1)  Imprecision (-1) |
| PN+OSM | MA | - | Very Low  Risk of bias (-1)  Imprecision (-1)  Intransitivity (-1) |
| PN+OSM | TEAS | - | Low  Risk of bias (-1)  Imprecision (-1) |
| PN+OSM | OSM | Low  Risk of bias (-1)  Imprecision (-1) | Low  Risk of bias (-1)  Imprecision (-1) |
| APA | STI | Low  Risk of bias (-1)  Imprecision (-1) | Low  Risk of bias (-1)  Imprecision (-1) |
| APA | PN | - | Very Low  Risk of bias (-1)  Inconsistency (-1)  Imprecision (-1)  Intransitivity (-1) |
| APA | APA+OSM | - | Very Low  Risk of bias (-1)  Inconsistency (-1)  Imprecision (-1)  Intransitivity (-1) |
| APA | MA | - | Very Low  Risk of bias (-1)  Inconsistency (-1)  Imprecision (-1)  Intransitivity (-1) |
| APA | TEAS | - | Very Low  Risk of bias (-1)  Inconsistency (-1)  Imprecision (-1)  Intransitivity (-1) |
| APA | OSM | Very Low  Risk of bias (-1)  Inconsistency (-1)  Imprecision (-1) | Very Low  Risk of bias (-1)  Inconsistency (-1)  Imprecision (-1) |
| STI | PN | - | Very Low  Risk of bias (-1)  Inconsistency (-1)  Imprecision (-1)  Intransitivity (-1) |
| STI | APA+OSM | - | Very Low  Risk of bias (-1)  Inconsistency (-1)  Imprecision (-1)  Intransitivity (-1) |
| STI | MA | - | Very Low  Risk of bias (-1)  Inconsistency (-1)  Imprecision (-1)  Intransitivity (-1) |
| STI | TEAS | - | Very Low  Risk of bias (-1)  Inconsistency (-1)  Imprecision (-1)  Intransitivity (-1) |
| STI | OSM | - | Very Low  Risk of bias (-1)  Inconsistency (-1)  Imprecision (-1)  Intransitivity (-1) |
| PN | APA+OSM | - | Very Low  Risk of bias (-1)  Inconsistency (-1)  Imprecision (-1)  Intransitivity (-1) |
| PN | MA | - | Very Low  Risk of bias (-1)  Imprecision (-1)  Intransitivity (-1) |
| PN | TEAS | - | Very Low  Risk of bias (-1)  Imprecision (-1)  Intransitivity (-1) |
| PN | OSM | Low  Risk of bias (-1)  Imprecision (-1) | Low  Risk of bias (-1)  Imprecision (-1) |
| APA+OSM | MA | - | Very Low  Risk of bias (-1)  Inconsistency (-1)  Imprecision (-1)  Intransitivity (-1) |
| APA+OSM | TEAS | - | Very Low  Risk of bias (-1)  Inconsistency (-1)  Imprecision (-1) |
| APA+OSM | OSM | Very Low  Risk of bias (-1)  Inconsistency (-1)  Imprecision (-1) | Very Low  Risk of bias (-1)  Inconsistency (-1)  Imprecision (-1) |
| MA | TEAS | - | Very Low  Risk of bias (-1)  Imprecision (-1)  Intransitivity (-1) |
| MA | OSM | Low  Risk of bias (-1)  Imprecision (-1) | Low  Risk of bias (-1)  Imprecision (-1) |
| TEAS | OSM | Low  Risk of bias (-1)  Imprecision (-1) | Low  Risk of bias (-1)  Imprecision (-1) |

Abbreviations: CCS, Cleveland Constipation Scale; OSM, osmotic laxatives; STI, stimulant laxatives; EA, electroacupuncture; MOX, moxibustion; PN, press needle; APA, acupoint application; MA, manual acupuncture; TEAS, transcutaneous electrical acupoint stimulation.

**Supplement 5B. Weekly SBMs**

| Comparison | | Direct evidence | Network meta-analysis |
| --- | --- | --- | --- |
| MOX+OSM | EA | - | Very Low  Risk of bias (-1)  Imprecision (-1)  Intransitivity (-1) |
| MOX+OSM | APA | - | Very Low  Risk of bias (-1)  Imprecision (-1)  Intransitivity (-1) |
| MOX+OSM | OSM | Low  Risk of bias (-1)  Imprecision (-1) | Low  Risk of bias (-1)  Imprecision (-1) |
| MOX+OSM | SA | - | Very Low  Risk of bias (-1)  Imprecision (-1)  Intransitivity (-1) |
| MOX+OSM | STI | - | Very Low  Risk of bias (-1)  Imprecision (-1)  Intransitivity (-1) |
| EA | APA | - | Very Low  Risk of bias (-1)  Imprecision (-1)  Intransitivity (-1) |
| EA | OSM | Low  Risk of bias (-1)  Imprecision (-1) | Low  Risk of bias (-1)  Imprecision (-1) |
| EA | SA | Moderate  Imprecision (-1) | Moderate  Imprecision (-1) |
| EA | STI | - | Very Low  Risk of bias (-1)  Imprecision (-1)  Intransitivity (-1) |
| APA | OSM | Low  Risk of bias (-1)  Imprecision (-1) | Low  Risk of bias (-1)  Imprecision (-1) |
| APA | SA | - | Very Low  Risk of bias (-1)  Imprecision (-1)  Intransitivity (-1) |
| APA | STI | Low  Risk of bias (-1)  Imprecision (-1) | Low  Risk of bias (-1)  Imprecision (-1) |
| OSM | SA | - | Very Low  Risk of bias (-1)  Imprecision (-1)  Intransitivity (-1) |
| OSM | STI | - | Very Low  Risk of bias (-1)  Imprecision (-1)  Intransitivity (-1) |
| SA | STI | - | Very Low  Risk of bias (-1)  Imprecision (-1)  Intransitivity (-1) |

Abbreviations: SBMs, spontaneous bowel movements per week; OSM, osmotic laxatives; STI, stimulant laxatives; MOX, moxibustion; EA, electroacupuncture; APA, acupoint application; SA, sham acupuncture.

**Supplement 5C. BFI**

| Comparison | | Direct evidence | Network meta-analysis |
| --- | --- | --- | --- |
| AA+OSM | AP+OSM | - | Very Low  Risk of bias (-1)  Imprecision (-1)  Intransitivity (-1) |
| AA+OSM | APA+OSM | - | Very Low  Risk of bias (-1)  Imprecision (-1)  Intransitivity (-1) |
| AA+OSM | OSM | Low  Risk of bias (-1)  Imprecision (-1) | Low  Risk of bias (-1)  Imprecision (-1) |
| AP+OSM | APA+OSM | - | Low  Risk of bias (-1)  Imprecision (-1) |
| AP+OSM | OSM | Low  Risk of bias (-1)  Imprecision (-1) | Low  Risk of bias (-1)  Imprecision (-1) |
| APA+OSM | OSM | Low  Risk of bias (-1)  Imprecision (-1) | Low  Risk of bias (-1)  Imprecision (-1) |

Abbreviations: BFI, Bowel Function Index; OSM, osmotic laxatives; AA, auricular acupuncture; AP, acupressure; APA, acupoint application.

**Supplement 5D. BFI: Feeling of incomplete bowel evacuation (1)**

| Comparison | | Direct evidence | Network meta-analysis |
| --- | --- | --- | --- |
| AA+OSM | APA+OSM | - | Very Low  Risk of bias (-1)  Imprecision (-1)  Intransitivity (-1) |
| AA+OSM | OSM | Low  Risk of bias (-1)  Imprecision (-1) | Low  Risk of bias (-1)  Imprecision (-1) |

Abbreviations: BFI, Bowel Function Index; OSM, osmotic laxatives; AA, auricular acupuncture; APA, acupoint application.

**Supplement 5E. BFI: Feeling of incomplete bowel evacuation (2)**

| Comparison | | Direct evidence | Network meta-analysis |
| --- | --- | --- | --- |
| APA | SA | Low  Risk of bias (-1)  Imprecision (-1) | Low  Risk of bias (-1)  Imprecision (-1) |
| APA | BC | Low  Risk of bias (-1)  Imprecision (-1) | Low  Risk of bias (-1)  Imprecision (-1) |
| SA | BC | **-** | Very Low  Risk of bias (-1)  Imprecision (-1)  Intransitivity (-1) |

Abbreviations: BFI, Bowel Function Index; BC, blank control; APA, acupoint application; SA, sham acupuncture.

**Supplement 5F. BFI: Ease of defecation (1)**

| Comparison | | Direct evidence | Network meta-analysis |
| --- | --- | --- | --- |
| AA+OSM | APA+OSM | - | Very Low  Risk of bias (-1)  Imprecision (-1)  Intransitivity (-1) |
| AA+OSM | OSM | Low  Risk of bias (-1)  Imprecision (-1) | Low  Risk of bias (-1)  Imprecision (-1) |

Abbreviations: BFI, Bowel Function Index; OSM, osmotic laxatives; AA, auricular acupuncture; APA, acupoint application.

**Supplement 5G. BFI: Ease of defecation (2)**

| Comparison | | Direct evidence | Network meta-analysis |
| --- | --- | --- | --- |
| APA | SA | Low  Risk of bias (-1)  Imprecision (-1) | Low  Risk of bias (-1)  Imprecision (-1) |
| APA | BC | Very Low  Risk of bias (-1)  Inconsistency (-1)  Imprecision (-1) | Very Low  Risk of bias (-1)  Inconsistency (-1)  Imprecision (-1) |
| SA | BC | **-** | Very Low  Risk of bias (-1)  Inconsistency (-1)  Imprecision (-1)  Intransitivity (-1) |

Abbreviations: BFI, Bowel Function Index; BC, blank control; APA, acupoint application; SA, sham acupuncture.

**Supplement 5H. BFI: Personal judgement of constipation (1)**

| Comparison | | Direct evidence | Network meta-analysis |
| --- | --- | --- | --- |
| AA+OSM | APA+OSM | **-** | Very Low  Risk of bias (-1)  Imprecision (-1)  Intransitivity (-1) |
| AA+OSM | OSM | Low  Risk of bias (-1)  Imprecision (-1) | Low  Risk of bias (-1)  Imprecision (-1) |

Abbreviations: BFI, Bowel Function Index; OSM, osmotic laxatives; AA, auricular acupuncture; APA, acupoint application.

**Supplement 5I. BFI: Personal judgement of constipation (2)**

| Comparison | | Direct evidence | Network meta-analysis |
| --- | --- | --- | --- |
| APA | SA | Low  Risk of bias (-1)  Imprecision (-1) | Low  Risk of bias (-1)  Imprecision (-1) |
| APA | BC | Very Low  Risk of bias (-1)  Inconsistency (-1)  Imprecision (-1) | Very Low  Risk of bias (-1)  Inconsistency (-1)  Imprecision (-1) |
| SA | BC | **-** | Very Low  Risk of bias (-1)  Inconsistency (-1)  Imprecision (-1)  Intransitivity (-1) |

Abbreviations: BFI, Bowel Function Index; BC, blank control; APA, acupoint application; SA, sham acupuncture.

**Supplement 5J. PAC-SYM**

| Comparison | | Direct evidence | Network meta-analysis |
| --- | --- | --- | --- |
| APA | OSM | Very Low  Risk of bias (-2)  Imprecision (-1) | Very Low  Risk of bias (-2)  Imprecision (-1) |
| APA | BC | Very Low  Risk of bias (-1)  Inconsistency (-1)  Imprecision (-1) | Very Low  Risk of bias (-1)  Inconsistency (-1)  Imprecision (-1) |
| OSM | BC | **-** | Very Low  Risk of bias (-2)  Inconsistency (-1)  Imprecision (-1)  Intransitivity (-1) |

Abbreviations: PAC-SYM, Patient Assessment of Constipation–Symptoms; OSM, osmotic laxatives; BC, blank control; APA, acupoint application.

**Supplement 5K. BSFS**

| Comparison | | Direct evidence | Network meta-analysis |
| --- | --- | --- | --- |
| AP | MOX | - | Very Low  Risk of bias (-1)  Inconsistency (-1)  Imprecision (-1)  Intransitivity (-1) |
| AP | PN+OSM | - | Very Low  Risk of bias (-1)  Inconsistency (-1)  Imprecision (-1)  Intransitivity (-1) |
| AP | AA+OSM | - | Very Low  Risk of bias (-1)  Inconsistency (-1)  Imprecision (-1)  Intransitivity (-1) |
| AP | EA | - | Very Low  Risk of bias (-1)  Inconsistency (-1)  Imprecision (-1)  Intransitivity (-1) |
| AP | APA+OSM | - | Very Low  Risk of bias (-1)  Inconsistency (-1)  Imprecision (-1)  Intransitivity (-1) |
| AP | SA | - | Very Low  Risk of bias (-1)  Inconsistency (-1)  Imprecision (-1)  Intransitivity (-1) |
| AP | APA | - | Very Low  Risk of bias (-1)  Imprecision (-1)  Intransitivity (-1) |
| AP | OSM | - | Very Low  Risk of bias (-1)  Inconsistency (-1)  Imprecision (-1)  Intransitivity (-1) |
| AP | BC | Low  Risk of bias (-1)  Imprecision (-1) | Low  Risk of bias (-1)  Imprecision (-1) |
| MOX | PN+OSM | - | Very Low  Risk of bias (-1)  Imprecision (-1)  Intransitivity (-1) |
| MOX | AA+OSM | - | Very Low  Risk of bias (-1)  Imprecision (-1)  Intransitivity (-1) |
| MOX | EA | - | Very Low  Risk of bias (-1)  Imprecision (-1)  Intransitivity (-1) |
| MOX | APA+OSM | - | Very Low  Risk of bias (-1)  Inconsistency (-1)  Imprecision (-1)  Intransitivity (-1) |
| MOX | SA | - | Very Low  Risk of bias (-1)  Imprecision (-1)  Intransitivity (-1) |
| MOX | APA | - | Very Low  Risk of bias (-1)  Inconsistency (-1)  Imprecision (-1)  Intransitivity (-1) |
| MOX | OSM | Low  Risk of bias (-1)  Imprecision (-1) | Low  Risk of bias (-1)  Imprecision (-1) |
| MOX | BC | - | Very Low  Risk of bias (-1)  Inconsistency (-1)  Imprecision (-1)  Intransitivity (-1) |
| PN+OSM | AA+OSM | - | Very Low  Risk of bias (-1)  Imprecision (-1)  Intransitivity (-1) |
| PN+OSM | EA | - | Very Low  Risk of bias (-1)  Imprecision (-1)  Intransitivity (-1) |
| PN+OSM | APA+OSM | - | Very Low  Risk of bias (-1)  Inconsistency (-1)  Imprecision (-1) |
| PN+OSM | SA | - | Very Low  Risk of bias (-1)  Imprecision (-1)  Intransitivity (-1) |
| PN+OSM | APA | - | Very Low  Risk of bias (-1)  Inconsistency (-1)  Imprecision (-1)  Intransitivity (-1) |
| PN+OSM | OSM | Low  Risk of bias (-1)  Imprecision (-1) | Low  Risk of bias (-1)  Imprecision (-1) |
| PN+OSM | BC | - | Very Low  Risk of bias (-1)  Inconsistency (-1)  Imprecision (-1)  Intransitivity (-1) |
| AA+OSM | EA | - | Very Low  Risk of bias (-1)  Imprecision (-1)  Intransitivity (-1) |
| AA+OSM | APA+OSM | - | Very Low  Risk of bias (-1)  Inconsistency (-1)  Imprecision (-1)  Intransitivity (-1) |
| AA+OSM | SA | - | Very Low  Risk of bias (-1)  Imprecision (-1)  Intransitivity (-1) |
| AA+OSM | APA | - | Very Low  Risk of bias (-1)  Inconsistency (-1)  Imprecision (-1)  Intransitivity (-1) |
| AA+OSM | OSM | Low  Risk of bias (-1)  Imprecision (-1) | Low  Risk of bias (-1)  Imprecision (-1) |
| AA+OSM | BC | - | Very Low  Risk of bias (-1)  Inconsistency (-1)  Imprecision (-1)  Intransitivity (-1) |
| EA | APA+OSM | - | Very Low  Risk of bias (-1)  Inconsistency (-1)  Imprecision (-1)  Intransitivity (-1) |
| EA | SA | Low  Risk of bias (-1)  Imprecision (-1) | Low  Risk of bias (-1)  Imprecision (-1) |
| EA | APA | - | Very Low  Risk of bias (-1)  Inconsistency (-1)  Imprecision (-1)  Intransitivity (-1) |
| EA | OSM | Low  Risk of bias (-1)  Imprecision (-1) | Low  Risk of bias (-1)  Imprecision (-1) |
| EA | BC | - | Very Low  Risk of bias (-1)  Inconsistency (-1)  Imprecision (-1)  Intransitivity (-1) |
| APA+OSM | SA | - | Very Low  Risk of bias (-1)  Inconsistency (-1)  Imprecision (-1)  Intransitivity (-1) |
| APA+OSM | APA | - | Very Low  Risk of bias (-1)  Inconsistency (-1)  Imprecision (-1)  Intransitivity (-1) |
| APA+OSM | OSM | Very Low  Risk of bias (-1)  Inconsistency (-1)  Imprecision (-1) | Very Low  Risk of bias (-1)  Inconsistency (-1)  Imprecision (-1) |
| APA+OSM | BC | - | Very Low  Risk of bias (-1)  Inconsistency (-1)  Imprecision (-1)  Intransitivity (-1) |
| SA | APA | - | Very Low  Risk of bias (-1)  Inconsistency (-1)  Imprecision (-1)  Intransitivity (-1) |
| SA | OSM | - | Very Low  Risk of bias (-1)  Imprecision (-1)  Intransitivity (-1) |
| SA | BC | - | Very Low  Risk of bias (-1)  Inconsistency (-1)  Imprecision (-1)  Intransitivity (-1) |
| APA | OSM | Very Low  Risk of bias (-1)  Inconsistency (-1)  Imprecision (-1) | Very Low  Risk of bias (-1)  Inconsistency (-1)  Imprecision (-1) |
| APA | BC | Low  Risk of bias (-1)  Imprecision (-1) | Low  Risk of bias (-1)  Imprecision (-1) |
| OSM | BC | - | Very Low  Risk of bias (-1)  Inconsistency (-1)  Imprecision (-1)  Intransitivity (-1) |

Abbreviations: BSFS, Bristol Stool Form Scale; OSM, osmotic laxatives; BC, blank control; AP, acupressure; MOX, moxibustion; PN, press needle; AA, auricular acupuncture; EA, electroacupuncture; APA, acupoint application; SA, sham acupuncture.

**Supplement 5L. PAC-QOL**

| Comparison | | Direct evidence | Network meta-analysis |
| --- | --- | --- | --- |
| MOX+OSM | PN+OSM | - | Low  Risk of bias (-1)  Imprecision (-1) |
| MOX+OSM | AA+OSM | - | Very Low  Risk of bias (-1)  Imprecision (-1)  Intransitivity (-1) |
| MOX+OSM | PN | - | Very Low  Risk of bias (-1)  Imprecision (-1)  Intransitivity (-1) |
| MOX+OSM | APA | - | Very Low  Risk of bias (-1)  Inconsistency (-1)  Imprecision (-1)  Intransitivity (-1) |
| MOX+OSM | APA+OSM | - | Low  Risk of bias (-1)  Imprecision (-1) |
| MOX+OSM | MA | - | Very Low  Risk of bias (-2)  Imprecision (-1)  Intransitivity (-1) |
| MOX+OSM | TEAS | - | Very Low  Risk of bias (-2)  Imprecision (-1) |
| MOX+OSM | OSM | Low  Risk of bias (-1)  Imprecision (-1) | Low  Risk of bias (-1)  Imprecision (-1) |
| PN+OSM | AA+OSM | - | Very Low  Risk of bias (-1)  Imprecision (-1)  Intransitivity (-1) |
| PN+OSM | PN | - | Very Low  Risk of bias (-1)  Imprecision (-1)  Intransitivity (-1) |
| PN+OSM | APA | - | Very Low  Risk of bias (-1)  Inconsistency (-1)  Imprecision (-1)  Intransitivity (-1) |
| PN+OSM | APA+OSM | - | Low  Risk of bias (-1)  Imprecision (-1) |
| PN+OSM | MA | - | Very Low  Risk of bias (-2)  Imprecision (-1)  Intransitivity (-1) |
| PN+OSM | TEAS | - | Very Low  Risk of bias (-2)  Imprecision (-1) |
| PN+OSM | OSM | Low  Risk of bias (-1)  Imprecision (-1) | Low  Risk of bias (-1)  Imprecision (-1) |
| AA+OSM | PN | - | Very Low  Risk of bias (-1)  Imprecision (-1)  Intransitivity (-1) |
| AA+OSM | APA | - | Very Low  Risk of bias (-1)  Inconsistency (-1)  Imprecision (-1)  Intransitivity (-1) |
| AA+OSM | APA+OSM | - | Very Low  Risk of bias (-1)  Imprecision (-1)  Intransitivity (-1) |
| AA+OSM | MA | - | Very Low  Risk of bias (-2)  Imprecision (-1)  Intransitivity (-1) |
| AA+OSM | TEAS | - | Very Low  Risk of bias (-2)  Imprecision (-1)  Intransitivity (-1) |
| AA+OSM | OSM | Low  Risk of bias (-1)  Imprecision (-1) | Low  Risk of bias (-1)  Imprecision (-1) |
| PN | APA | - | Very Low  Risk of bias (-1)  Inconsistency (-1)  Imprecision (-1)  Intransitivity (-1) |
| PN | APA+OSM | - | Very Low  Risk of bias (-1)  Imprecision (-1)  Intransitivity (-1) |
| PN | MA | - | Very Low  Risk of bias (-2)  Imprecision (-1)  Intransitivity (-1) |
| PN | TEAS | - | Very Low  Risk of bias (-2)  Imprecision (-1)  Intransitivity (-1) |
| PN | OSM | Low  Risk of bias (-1)  Imprecision (-1) | Low  Risk of bias (-1)  Imprecision (-1) |
| APA | APA+OSM | - | Very Low  Risk of bias (-1)  Inconsistency (-1)  Imprecision (-1)  Intransitivity (-1) |
| APA | MA | - | Very Low  Risk of bias (-2)  Inconsistency (-1)  Imprecision (-1)  Intransitivity (-1) |
| APA | TEAS | - | Very Low  Risk of bias (-2)  Inconsistency (-1)  Imprecision (-1)  Intransitivity (-1) |
| APA | OSM | Very Low  Risk of bias (-1)  Inconsistency (-1)  Imprecision (-1) | Very Low  Risk of bias (-1)  Inconsistency (-1)  Imprecision (-1) |
| APA+OSM | MA | - | Very Low  Risk of bias (-2)  Imprecision (-1)  Intransitivity (-1) |
| APA+OSM | TEAS | - | Very Low  Risk of bias (-2)  Imprecision (-1) |
| APA+OSM | OSM | Low  Risk of bias (-1)  Imprecision (-1) | Low  Risk of bias (-1)  Imprecision (-1) |
| MA | TEAS | - | Very Low  Risk of bias (-2)  Imprecision (-1)  Intransitivity (-1) |
| MA | OSM | Very Low  Risk of bias (-2)  Imprecision (-1) | Very Low  Risk of bias (-2)  Imprecision (-1) |
| TEAS | OSM | Very Low  Risk of bias (-2)  Imprecision (-1) | Very Low  Risk of bias (-2)  Imprecision (-1) |

Abbreviations: PAC-QOL, Patient Assessment of Constipation Quality of Life questionnaire; MOX, moxibustion; PN, press needle; AA, auricular acupuncture; APA, acupoint application; MA, manual acupuncture; TEAS, transcutaneous electrical acupoint stimulation.

**Supplement 5M. PAC-QOL: Worries and concerns**

| Comparison | | Direct evidence | Network meta-analysis |
| --- | --- | --- | --- |
| APA | SA | Low  Risk of bias (-1)  Imprecision (-1) | Low  Risk of bias (-1)  Imprecision (-1) |
| APA | AP | - | Very Low  Risk of bias (-2)  Inconsistency (-1)  Imprecision (-1)  Intransitivity (-1) |
| APA | BC | Very Low  Risk of bias (-1)  Inconsistency (-1)  Imprecision (-1) | Very Low  Risk of bias (-1)  Inconsistency (-1)  Imprecision (-1) |
| SA | AP | - | Very Low  Risk of bias (-2)  Inconsistency (-1)  Imprecision (-1)  Intransitivity (-1) |
| SA | BC | - | Very Low  Risk of bias (-1)  Inconsistency (-1)  Imprecision (-1)  Intransitivity (-1) |
| AP | BC | Very Low  Risk of bias (-2)  Imprecision (-1) | Very Low  Risk of bias (-2)  Imprecision (-1) |

Abbreviations: PAC-QOL, Patient Assessment of Constipation Quality of Life questionnaire; BC, blank control; APA, acupoint application; SA, sham acupuncture; AP, acupressure.

**Supplement 5N. PAC-QOL: Physical**

| Comparison | | Direct evidence | Network meta-analysis |
| --- | --- | --- | --- |
| APA | SA | Low  Risk of bias (-1)  Imprecision (-1) | Low  Risk of bias (-1)  Imprecision (-1) |
| APA | AP | - | Very Low  Risk of bias (-2)  Inconsistency (-1)  Imprecision (-1)  Intransitivity (-1) |
| APA | BC | Very Low  Risk of bias (-1)  Inconsistency (-1)  Imprecision (-1) | Very Low  Risk of bias (-1)  Inconsistency (-1)  Imprecision (-1) |
| SA | AP | - | Very Low  Risk of bias (-2)  Inconsistency (-1)  Imprecision (-1)  Intransitivity (-1) |
| SA | BC | - | Very Low  Risk of bias (-1)  Inconsistency (-1)  Imprecision (-1)  Intransitivity (-1) |
| AP | BC | Very Low  Risk of bias (-2)  Imprecision (-1) | Very Low  Risk of bias (-2)  Imprecision (-1) |

Abbreviations: PAC-QOL, Patient Assessment of Constipation Quality of Life questionnaire; BC, blank control; APA, acupoint application; AP, acupressure; SA, sham acupuncture.

**Supplement 5O. PAC-QOL: Satisfaction**

| Comparison | | Direct evidence | Network meta-analysis |
| --- | --- | --- | --- |
| APA | SA | Low  Risk of bias (-1)  Imprecision (-1) | Low  Risk of bias (-1)  Imprecision (-1) |
| APA | AP | - | Very Low  Risk of bias (-2)  Inconsistency (-1)  Imprecision (-1)  Intransitivity (-1) |
| APA | BC | Very Low  Risk of bias (-1)  Inconsistency (-1)  Imprecision (-1) | Very Low  Risk of bias (-1)  Inconsistency (-1)  Imprecision (-1) |
| SA | AP | - | Very Low  Risk of bias (-2)  Inconsistency (-1)  Imprecision (-1)  Intransitivity (-1) |
| SA | BC | - | Very Low  Risk of bias (-1)  Inconsistency (-1)  Imprecision (-1)  Intransitivity (-1) |
| AP | BC | Very Low  Risk of bias (-2)  Imprecision (-1) | Very Low  Risk of bias (-2)  Imprecision (-1) |

Abbreviations: PAC-QOL, Patient Assessment of Constipation Quality of Life questionnaire; BC, blank control; APA, acupoint application; SA, sham acupuncture; AP, acupressure.

**Supplement 5P. PAC-QOL: Psychosocial**

| Comparison | | Direct evidence | Network meta-analysis |
| --- | --- | --- | --- |
| APA | SA | Low  Risk of bias (-1)  Imprecision (-1) | Low  Risk of bias (-1)  Imprecision (-1) |
| APA | AP | - | Very Low  Risk of bias (-2)  Inconsistency (-1)  Imprecision (-1)  Intransitivity (-1) |
| APA | BC | Very Low  Risk of bias (-1)  Inconsistency (-1)  Imprecision (-1) | Very Low  Risk of bias (-1)  Inconsistency (-1)  Imprecision (-1) |
| SA | AP | - | Very Low  Risk of bias (-2)  Inconsistency (-1)  Imprecision (-1)  Intransitivity (-1) |
| SA | BC | - | Very Low  Risk of bias (-1)  Inconsistency (-1)  Imprecision (-1)  Intransitivity (-1) |
| AP | BC | Very Low  Risk of bias (-2)  Imprecision (-1) | Very Low  Risk of bias (-2)  Imprecision (-1) |

Abbreviations: PAC-QOL, Patient Assessment of Constipation Quality of Life questionnaire; BC, blank control; APA, acupoint application; SA, sham acupuncture; AP, acupressure.

**Supplement 6. Network plot**

**Supplement 6A. BFI: Feeling of Incomplete Bowel Evacuation (1)**

Abbreviations: BFI, Bowel Function Index; OSM, osmotic laxatives; AA, auricular acupuncture; APA, acupoint application.

**Supplement 6B. BFI: Feeling of Incomplete Bowel Evacuation (2)**

Abbreviations: BFI, Bowel Function Index; BC, blank control; APA, acupoint application; SA, sham acupuncture.

**Supplement 6C. BFI: Ease of Defecation (1)**

Abbreviations: BFI, Bowel Function Index; OSM, osmotic laxatives; AA, auricular acupuncture; APA, acupoint application.

**Supplement 6D. BFI: Ease of Defecation (2)**

Abbreviations: BFI, Bowel Function Index; BC, blank control; APA, acupoint application; SA, sham acupuncture.

**Supplement 6E. BFI: Personal judgement of constipation (1)**

Abbreviations: BFI, Bowel Function Index; OSM, osmotic laxatives; AA, auricular acupuncture; APA, acupoint application.

**Supplement 6F. BFI: Personal judgement of constipation (2)**

Abbreviations: BFI, Bowel Function Index; BC, blank control; APA, acupoint application; SA, sham acupuncture.

**Supplement 6G. PAC-QOL**

Abbreviations: PAC-QOL, Patient Assessment of Constipation Quality of Life questionnaire; MOX, moxibustion; PN, press needle; AA, auricular acupuncture; APA, acupoint application; MA, manual acupuncture; TEAS, transcutaneous electrical acupoint stimulation.

**Supplement 6H. PAC-QOL: Worries and concerns**

Abbreviations: PAC-QOL, Patient Assessment of Constipation Quality of Life questionnaire; BC, blank control; APA, acupoint application; SA, sham acupuncture; AP, acupressure.

**Supplement 6I. PAC-QOL: Physical**

Abbreviations: PAC-QOL, Patient Assessment of Constipation Quality of Life questionnaire; BC, blank control; APA, acupoint application; SA, sham acupuncture; AP, acupressure.

**Supplement 6J. PAC-QOL: Satisfaction**

Abbreviations: PAC-QOL, Patient Assessment of Constipation Quality of Life questionnaire; BC, blank control; APA, acupoint application; SA, sham acupuncture; AP, acupressure.

**Supplement 6K. PAC-QOL: Psychosocial**

Abbreviations: PAC-QOL, Patient Assessment of Constipation Quality of Life questionnaire; BC, blank control; APA, acupoint application; SA, sham acupuncture; AP, acupressure.

**Supplement 7. SUCRA plot**

**Supplement 7A. BFI: Feeling of Incomplete Bowel Evacuation (1)**

Abbreviations: BFI, Bowel Function Index; OSM, osmotic laxatives; AA, auricular acupuncture; APA, acupoint application.

**Supplement 7B. BFI: Feeling of Incomplete Bowel Evacuation (2)**

Abbreviations: BFI, Bowel Function Index; BC, blank control; APA, acupoint application; SA, sham acupuncture.

**Supplement 7C. BFI: Ease of Defecation (1)**

Abbreviations: BFI, Bowel Function Index; OSM, osmotic laxatives; AA, auricular acupuncture; APA, acupoint application.

**Supplement 7D. BFI: Ease of Defecation (2)**

Abbreviations: BFI, Bowel Function Index; BC, blank control; APA, acupoint application; SA, sham acupuncture.

**Supplement 7E. BFI: Personal judgement of constipation (1)**

Abbreviations: BFI, Bowel Function Index; OSM, osmotic laxatives; AA, auricular acupuncture; APA, acupoint application.

**Supplement 7F. SUCRA plot: BFI: Personal judgement of constipation (2)**

Abbreviations: BFI, Bowel Function Index; BC, blank control; APA, acupoint application; SA, sham acupuncture.

**Supplement 7G. PAC-QOL**

Abbreviations: PAC-QOL, Patient Assessment of Constipation Quality of Life questionnaire; MOX, moxibustion; PN, press needle; AA, auricular acupuncture; APA, acupoint application; MA, manual acupuncture; TEAS, transcutaneous electrical acupoint stimulation.

**Supplement 7H. PAC-QOL: Worries and concerns**

Abbreviations: PAC-QOL, Patient Assessment of Constipation Quality of Life questionnaire; BC, blank control; APA, acupoint application; SA, sham acupuncture; AP, acupressure.

**Supplement 7I. PAC-QOL: Physical**

Abbreviations: PAC-QOL, Patient Assessment of Constipation Quality of Life questionnaire; BC, blank control; APA, acupoint application; SA, sham acupuncture; AP, acupressure.

**Supplement 7J. PAC-QOL: Satisfaction**

Abbreviations: PAC-QOL, Patient Assessment of Constipation Quality of Life questionnaire; BC, blank control; APA, acupoint application; SA, sham acupuncture; AP, acupressure.

**Supplement 7K. PAC-QOL: Psychosocial**

Abbreviations: PAC-QOL, Patient Assessment of Constipation Quality of Life questionnaire; BC, blank control; APA, acupoint application; SA, sham acupuncture; AP, acupressure.

**Supplement 8. Funnel plot**

**Supplement 8A. Funnel plot: CCS**

A, TEAS; B, OSM; C, EA; D, APA; E, STI; F, PN; G, MA; H, MOX; I, APA+OSM; J, PN+OSM; K, MA+OSM

Abbreviations: CCS, Cleveland Constipation Scale; OSM, osmotic laxatives; STI, stimulant laxatives; EA, electroacupuncture; MOX, moxibustion; PN, press needle; APA, acupoint application; MA, manual acupuncture; TEAS, transcutaneous electrical acupoint stimulation.

**Supplement 8B. Funnel plot: BSFS**

A, APA; B, BC; C, AP; D, EA; E, SA; F, OSM; G, MOX; H, APA+OSM; I, AA+OSM; J, PN+OSM

Abbreviations: BSFS, Bristol Stool Form Scale; OSM, osmotic laxatives; BC, blank control; AP, acupressure; MOX, moxibustion; PN, press needle; AA, auricular acupuncture; EA, electroacupuncture; APA, acupoint application; SA, sham acupuncture.

**Supplement 8C. Funnel plot: PAC-QOL**

A, TEAS; B, OSM; C, APA; D, PN; E, MA; F, APA+OSM; G, MOX+OSM; H, AA+OSM; I, PN+OSM

Abbreviations: PAC-QOL, Patient Assessment of Constipation Quality of Life questionnaire; MOX, moxibustion; PN, press needle; AA, auricular acupuncture; APA, acupoint application; MA, manual acupuncture; TEAS, transcutaneous electrical acupoint stimulation.

**Supplement 9. Detailed RoB 2 judgments and supporting reasons for each included study**

| **Study** | **Outcome** | **D1. Randomization process** | **D2. Deviations from intended interventions** | **D3. Missing outcome data** | **D4. Measurement of the outcome** | **D5. Selection of the reported result** | **Overall risk of bias** |
| --- | --- | --- | --- | --- | --- | --- | --- |
| Wang 2023 | ② ④ | **Low risk.** A centralized web-based randomization system with permuted blocks provided adequate sequence generation and allocation concealment. | **Low risk.** Blinding, comparable treatment schedules, prespecified rescue criteria, and intention-to-treat analyses minimized deviations from the assigned interventions. | **Low risk.** Limited missing stool-diary data were handled by multiple imputation, with consistent sensitivity analyses. | **Low risk.** Participants were blinded using a sham electroacupuncture procedure, and BSFS and weekly SBMs were prospectively recorded in standardized stool diaries using prespecified definitions, with no evidence that knowledge of allocation influenced outcome measurement. | **Low risk.** The protocol and prespecified analysis plan were available, and the reported outcomes and analyses were consistent with them. | **Low risk in all domains.** |
| Yildirim 2022 | ⑩ ⑪ ⑫ ⑬ | **Some concerns.** A computer-generated randomization list was used, but allocation concealment was not described and the list was prepared by a study researcher. | **High risk.** Participants were excluded after randomization because of adherence, and the analysis did not appropriately estimate the effect of assignment to intervention. | **High risk.** Thirty participants per group were excluded for differing reasons, and no imputation or sensitivity analysis was reported. | **Some concerns.** PAC-QOL subscale scores were participant-reported by unblinded participants, but no direct evidence showed that knowledge of allocation materially influenced reporting. | **Some concerns.** Trial registration was reported, but detailed prespecified analyses were unavailable for comparison. | **High risk due to D2 and D3.** |
| Cai 2019 | ⑥ ⑦ ⑧ | **Some concerns.** A random-number table was used, but allocation concealment and independence of enrolment were not described. | **Low risk.** Both groups received the same lactulose regimen and background care, with no important unbalanced co-interventions. | **High risk.** Participants who switched therapy because of severe constipation were excluded, making missingness outcome-related without appropriate analysis. | **Low risk**. A sham stimulation control was used, and the BFI subscale outcomes were assessed in both groups using the same standardized instruments and procedures, with no evidence of differential outcome measurement. | **Some concerns.** No prespecified analysis plan was available; selection among multiple eligible measurements or analyses could not be excluded. | **High risk due to D3.** |
| Zhu 2018 | ① ⑨ | **Some concerns.** An SPSS-generated random sequence was used, but allocation concealment and protection against foreknowledge were not reported. | **Low risk.** No important deviations arising from the trial context or imbalances in co-interventions were identified. | **High risk.** Completion and analysis counts were substantially inconsistent, and withdrawals and other losses were not reconciled or appropriately analysed. | **High risk.** CCS and PAC-QOL were participant-reported by unblinded participants, and the authors acknowledged that treatment expectations could influence assessment. | **Some concerns.** Trial registration was reported, but no prespecified statistical analysis plan was available for comparison. | **High risk due to D3 and D4.** |
| Chen 2021 | ⑤ | **Some concerns.** Randomization was reported, but allocation concealment was insufficiently described. | **Low risk.** No important deviations arising from the trial context or imbalances in co-interventions were identified. | **Low risk.** Outcome data were available for all randomized participants. | **Some concerns.** BFI was participant-reported by unblinded participants, but no clear evidence showed that knowledge of allocation materially influenced reporting. | **Some concerns.** No prespecified analysis plan was available; selection among multiple eligible measurements or analyses could not be excluded. | **Some concerns due to D1, D4 and D5.** |
| Deng 2018 | ④ ⑨ | **Some concerns.** Randomization was reported, but allocation concealment was insufficiently described. | **Low risk.** Rescue treatment was prespecified and no important deviations arising from the trial context were identified. | **Some concerns.** Four of 94 participants were excluded, but the reasons and group distribution were insufficiently reported. | **Some concerns.** Weekly SBMs were participant-recorded and PAC-QOL was participant-reported by unblinded participants, but no clear evidence showed that knowledge of allocation materially influenced reporting. | **Some concerns.** No prespecified analysis plan was available; selection among multiple eligible measurements or analyses could not be excluded. | **Some concerns due to D1, D3, D4 and D5.** |
| Du 2012 | ① | **Some concerns.** Randomization was reported, but allocation concealment was insufficiently described. | **Low risk.** No important deviations arising from the trial context or imbalances in co-interventions were identified. | **Low risk.** Outcome data were available for all randomized participants. | **Some concerns.** CCS was participant-reported by unblinded participants, but no clear evidence showed that knowledge of allocation materially influenced reporting. | **Some concerns.** No prespecified analysis plan was available; selection among multiple eligible measurements or analyses could not be excluded. | **Some concerns due to D1, D4 and D5.** |
| He 2024 | ③ | **Some concerns.** Randomization was reported, but allocation concealment was insufficiently described. | **Low risk.** No important deviations arising from the trial context or imbalances in co-interventions were identified. | **High risk.** Fourteen of 120 participants were excluded for disease deterioration or rescue-laxative use, including poor outcomes, without appropriate missing-data analysis. | **Some concerns.** PAC-SYM was participant-reported by unblinded participants, but no clear evidence showed that knowledge of allocation materially influenced reporting. | **Some concerns.** No prespecified analysis plan was available; selection among multiple eligible measurements or analyses could not be excluded. | **High risk due to D3.** |
| Li 2020 | ② ④ | **Some concerns.** Randomization was reported, but sequence generation and allocation concealment were insufficiently described. | **Low risk.** No important deviations arising from the trial context or imbalances in co-interventions were identified. | **Some concerns.** Two participants in the lactulose group were excluded for potentially outcome-related reasons; although the amount of missing data was small, its influence on the result could not be fully excluded. | **Some concerns.** BSFS and weekly SBMs were participant-recorded by unblinded participants, but no clear evidence showed that knowledge of allocation materially influenced reporting. | **Some concerns.** No prespecified analysis plan was available; selection among multiple eligible measurements or analyses could not be excluded. | **Some concerns due to D1, D3, D4 and D5.** |
| Li 2024 | ② | **Some concerns.** Randomization was reported, but allocation concealment was insufficiently described. | **Low risk.** No important deviations arising from the trial context or imbalances in co-interventions were identified. | **Low risk.** Outcome data were available for all randomized participants. | **Some concerns.** BSFS was participant-reported by unblinded participants, but no clear evidence showed that knowledge of allocation materially influenced reporting. | **Some concerns.** No prespecified analysis plan was available; selection among multiple eligible measurements or analyses could not be excluded. | **Some concerns due to D1, D4 and D5.** |
| Liang 2017 | ② ⑥ ⑦ ⑧ | **Some concerns.** Randomization was reported, but sequence generation and allocation concealment were insufficiently described. | **Low risk.** No important deviations arising from the trial context or imbalances in co-interventions were identified. | **Low risk.** Outcome data were available for all randomized participants. | **Some concerns.** BSFS and the BFI subscale outcomes were participant-reported by unblinded participants, but no clear evidence showed that knowledge of allocation materially influenced reporting. | **Some concerns.** No prespecified analysis plan was available; selection among multiple eligible measurements or analyses could not be excluded. | **Some concerns due to D1, D4 and D5.** |
| Lin 2021 | ① ② ⑨ | **Some concerns.** A computer-generated random sequence was used, but allocation concealment was not reported. | **Low risk.** No important deviations arising from the trial context or imbalances in co-interventions were identified. | **Some concerns.** Five of 78 participants were excluded for opioid-dose changes or loss to follow-up, with no imputation or sensitivity analysis. | **Some concerns.** CCS, BSFS and PAC-QOL were participant-reported by unblinded participants, but no clear evidence showed that knowledge of allocation materially influenced reporting. | **Some concerns.** No prespecified analysis plan was available; selection among multiple eligible measurements or analyses could not be excluded. | **Some concerns due to D1, D3, D4 and D5.** |
| Lin 2014 | ① | **Some concerns.** Randomization was reported, but allocation concealment was insufficiently described. | **Low risk.** No important deviations arising from the trial context or imbalances in co-interventions were identified. | **Low risk.** Outcome data were available for all randomized participants. | **Some concerns.** CCS was participant-reported by unblinded participants, but no clear evidence showed that knowledge of allocation materially influenced reporting. | **Some concerns.** No prespecified analysis plan was available; selection among multiple eligible measurements or analyses could not be excluded. | **Some concerns due to D1, D4 and D5.** |
| Liu 2022 | ② | **Some concerns.** Randomization was reported, but allocation concealment was insufficiently described. | **Low risk.** No important deviations arising from the trial context or imbalances in co-interventions were identified. | **Some concerns.** Four of 70 participants were excluded, but reasons and group distribution were insufficiently reported and no missing-data analysis was performed. | **Some concerns.** BSFS was participant-reported by unblinded participants, but no clear evidence showed that knowledge of allocation materially influenced reporting. | **Some concerns.** No prespecified analysis plan was available; selection among multiple eligible measurements or analyses could not be excluded. | **Some concerns due to D1, D3, D4 and D5.** |
| Ma 2023 | ③ ⑥ ⑦ ⑧ ⑩ ⑪ ⑫ ⑬ | **Some concerns.** Randomization was reported, but allocation concealment was not described and a baseline age imbalance was observed. | **Low risk.** No important deviations arising from the trial context or imbalances in co-interventions were identified. | **Low risk.** Outcome data were available for all randomized participants. | **Some concerns.** PAC-SYM, PAC-QOL and BFI were participant-reported by unblinded participants, but no clear evidence showed that knowledge of allocation materially influenced reporting. | **Some concerns.** No prespecified analysis plan was available; selection among multiple eligible measurements or analyses could not be excluded. | **Some concerns due to D1, D4 and D5.** |
| Ma 2019 | ① ⑨ | **Some concerns.** Randomization was reported, but sequence generation and allocation concealment were insufficiently described. | **Low risk.** No important deviations arising from the trial context or imbalances in co-interventions were identified. | **Low risk.** Outcome data were available for all randomized participants. | **Some concerns.** CCS and PAC-QOL were participant-reported by unblinded participants, but no clear evidence showed that knowledge of allocation materially influenced reporting. | **Some concerns.** No prespecified analysis plan was available; selection among multiple eligible measurements or analyses could not be excluded. | **Some concerns due to D1, D4 and D5.** |
| Mao 2017 | ① | **Some concerns.** Randomization was reported, but allocation concealment was insufficiently described. | **Low risk.** Rescue treatment after more than 3 days without defecation was prespecified, with no other important deviations identified. | **Low risk.** Outcome data were available for all randomized participants. | **Some concerns.** CCS was participant-reported by unblinded participants, but no clear evidence showed that knowledge of allocation materially influenced reporting. | **Some concerns.** No prespecified analysis plan was available; selection among multiple eligible measurements or analyses could not be excluded. | **Some concerns due to D1, D4 and D5.** |
| Ouyang 2023 | ① ② ⑨ | **Some concerns.** Randomization was reported, but allocation concealment was insufficiently described. | **Low risk.** No important deviations arising from the trial context or imbalances in co-interventions were identified. | **Low risk.** Outcome data were available for all randomized participants. | **Some concerns.** CCS, BSFS and PAC-QOL were participant-reported by unblinded participants, but no clear evidence showed that knowledge of allocation materially influenced reporting. | **Some concerns.** No prespecified analysis plan was available; selection among multiple eligible measurements or analyses could not be excluded. | **Some concerns due to D1, D4 and D5.** |
| Qi 2021 | ① ② | **Low risk.** A computer-generated random sequence and independently managed sequentially numbered opaque envelopes were used, with no important baseline imbalance. | **Some concerns.** Participants using other treatments after randomization were excluded, so the analysis did not fully follow the intention-to-treat principle. | **Some concerns.** Five of 70 participants were not analysed after transfer, discharge, or other medication use, with no imputation or sensitivity analysis. | **Some concerns.** CCS and BSFS were participant-reported by unblinded participants, but no clear evidence showed that knowledge of allocation materially influenced reporting. | **Some concerns.** No prespecified analysis plan was available; selection among multiple eligible measurements or analyses could not be excluded. | **Some concerns due to D2, D3, D4 and D5.** |
| Qu 2016 | ① | **Some concerns.** Randomization was reported, but allocation concealment was insufficiently described. | **Low risk.** No important deviations arising from the trial context or imbalances in co-interventions were identified. | **Low risk.** Outcome data were available for all randomized participants. | **Some concerns.** CCS was participant-reported by unblinded participants, but no clear evidence showed that knowledge of allocation materially influenced reporting. | **Some concerns.** No prespecified analysis plan was available; selection among multiple eligible measurements or analyses could not be excluded. | **Some concerns due to D1, D4 and D5.** |
| Tang 2019 | ⑨ | **Some concerns.** Randomization was reported, but sequence generation and allocation concealment were insufficiently described. | **Low risk.** No important deviations arising from the trial context or imbalances in co-interventions were identified. | **Low risk.** Outcome data were available for all randomized participants. | **Some concerns.** PAC-QOL was participant-reported by unblinded participants, but no clear evidence showed that knowledge of allocation materially influenced reporting. | **Some concerns.** No prespecified analysis plan was available; selection among multiple eligible measurements or analyses could not be excluded. | **Some concerns due to D1, D4 and D5.** |
| Tian 2020 | ① ⑨ | **Low risk.** A computer-generated random sequence and independently managed sealed opaque envelopes were used, with no important baseline imbalance. | **High risk.** Participants withdrew because of treatment expectations or perceived improvement, and the analysis excluded them rather than estimating the effect of assignment. | **High risk.** Five of 70 participants were not analysed for reasons related to treatment expectations or perceived outcomes, with no appropriate missing-data analysis. | **High risk.** CCS and PAC-QOL were participant-reported, and withdrawals related to treatment expectations or perceived improvement indicated likely material influence on reporting. | **Some concerns.** No prespecified analysis plan was available; selection among multiple eligible measurements or analyses could not be excluded. | **High risk due to D2, D3 and D4.** |
| Wang 2017 | ④ | **Some concerns.** Randomization was reported, but sequence generation and allocation concealment were insufficiently described. | **Low risk.** No important deviations arising from the trial context or imbalances in co-interventions were identified. | **Some concerns.** One withdrawal was reported, but the outcome tables still included all participants and its handling was unclear. | **Some concerns.** Weekly SBMs were participant-recorded by unblinded participants, but no clear evidence showed that knowledge of allocation materially influenced reporting. | **Some concerns.** No prespecified analysis plan was available; selection among multiple eligible measurements or analyses could not be excluded. | **Some concerns due to D1, D3, D4 and D5.** |
| Xia 2024 | ③ ⑩ ⑪ ⑫ ⑬ | **Some concerns.** Randomization was reported, but allocation concealment was insufficiently described. | **Low risk.** No important deviations arising from the trial context or imbalances in co-interventions were identified. | **Low risk.** Outcome data were available for all randomized participants. | **Some concerns.** PAC-SYM and PAC-QOL subscale scores were participant-reported by unblinded participants, but no clear evidence showed that knowledge of allocation materially influenced reporting. | **Some concerns.** No prespecified analysis plan was available; selection among multiple eligible measurements or analyses could not be excluded. | **Some concerns due to D1, D4 and D5.** |
| Xu 2015 | ② | **Some concerns.** Randomization was reported, but allocation concealment was insufficiently described. | **Low risk.** Rescue treatment after more than 3 days without defecation was prespecified, with no other important deviations identified. | **Low risk.** Outcome data were available for all randomized participants. | **Some concerns.** BSFS was participant-reported by unblinded participants, but no clear evidence showed that knowledge of allocation materially influenced reporting. | **Some concerns.** No prespecified analysis plan was available; selection among multiple eligible measurements or analyses could not be excluded. | **Some concerns due to D1, D4 and D5.** |
| Xu 2021a | ② ④ ⑨ | **Some concerns.** Randomization was reported, but sequence generation and allocation concealment were insufficiently described. | **Low risk.** Both groups received the assigned lactulose regimen, with no important deviations or imbalances in co-interventions identified. | **Low risk.** Outcome data were available for all randomized participants. | **Some concerns.** BSFS and PAC-QOL were participant-reported and weekly SBMs were participant-recorded by unblinded participants, but no clear evidence showed that knowledge of allocation materially influenced reporting. | **Some concerns.** No prespecified analysis plan was available; selection among multiple eligible measurements or analyses could not be excluded. | **Some concerns due to D1, D4 and D5.** |
| Xu 2021b | ① | **Some concerns.** Randomization was reported, but allocation concealment was insufficiently described. | **Low risk.** No important deviations arising from the trial context or imbalances in co-interventions were identified. | **Low risk.** Outcome data were available for all randomized participants. | **Some concerns.** CCS was participant-reported by unblinded participants, but no clear evidence showed that knowledge of allocation materially influenced reporting. | **Some concerns.** No prespecified analysis plan was available and the reported CCS values were internally inconsistent, so selective reporting or error could not be excluded. | **Some concerns due to D1, D4 and D5.** |
| Yang 2014 | ② ⑤ ⑥ ⑦ ⑧ ⑨ | **Some concerns.** Randomization was reported, but allocation concealment was insufficiently described. | **Low risk.** Both groups received the assigned treatment and background care, with no important deviations identified. | **Low risk.** Outcome data were available for all randomized participants. | **Some concerns.** BSFS, the BFI total and subscale outcomes, and PAC-QOL were participant-reported by unblinded participants, but no clear evidence showed that knowledge of allocation materially influenced reporting. | **Some concerns.** No prespecified analysis plan was available; selection among multiple eligible measurements or analyses could not be excluded. | **Some concerns due to D1, D4 and D5.** |
| Yang 2019 | ⑤ | **Some concerns.** Randomization was reported, but allocation concealment was insufficiently described. | **Low risk.** Both groups received the assigned interventions and comparable background care, with no important deviations identified. | **Low risk.** Outcome data were available for all randomized participants. | **Some concerns.** BFI was participant-reported by unblinded participants, but no clear evidence showed that knowledge of allocation materially influenced reporting. | **Some concerns.** No prespecified analysis plan was available; selection among multiple eligible measurements or analyses could not be excluded. | **Some concerns due to D1, D4 and D5.** |
| Yin 2018 | ① | **Some concerns.** Allocation concealment was not described, and a notable baseline imbalance in CCS scores was present. | **Some concerns.** Post-randomization treatment discontinuation and rescue-enema use were insufficiently reported by group. | **Low risk.** Outcome data were available for all randomized participants. | **Some concerns.** CCS was participant-reported by unblinded participants, but no clear evidence showed that knowledge of allocation materially influenced reporting. | **Some concerns.** No prespecified analysis plan was available, and selection among eligible time points or analyses could not be excluded. | **Some concerns due to D1, D2, D4 and D5.** |
| Zhang 2023 | ① ② ⑨ | **Some concerns.** Randomization was reported, but allocation concealment was insufficiently described. | **Low risk.** Both groups received the assigned interventions, with no important deviations or imbalances in co-interventions identified. | **Low risk.** Outcome data were available for all randomized participants. | **Some concerns.** CCS, BSFS and PAC-QOL were participant-reported by unblinded participants, but no clear evidence showed that knowledge of allocation materially influenced reporting. | **Some concerns.** No prespecified analysis plan was available; selection among multiple eligible measurements or analyses could not be excluded. | **Some concerns due to D1, D4 and D5.** |
| Zhao 2021 | ⑥ ⑦ ⑧ ⑩ ⑪ ⑫ ⑬ | **Some concerns.** Systematic randomization was reported, but sequence generation and allocation concealment were insufficiently described. | **Low risk.** Both groups received comparable background care and prespecified rescue treatment, with no important unbalanced deviations identified. | **Low risk.** Outcome data were available for all randomized participants. | **Low risk.** A placebo patch control was used, and BFI and PAC-QOL subscale outcomes were assessed in both groups using the same standardized instruments and procedures, with no evidence of differential outcome measurement. | **Some concerns.** No prespecified analysis plan was available; selection among multiple eligible measurements or analyses could not be excluded. | **Some concerns due to D1 and D5.** |
| Zheng 2022 | ② | **Some concerns.** Participants were assigned using odd or even random numbers, but allocation concealment was not reported. | **Some concerns.** Four participants were excluded after randomization, so the analysis did not fully follow the intention-to-treat principle. | **Some concerns.** Four of 70 participants were not analysed for varied reasons, and no imputation or sensitivity analysis was reported. | **Some concerns.** BSFS was participant-reported by unblinded participants, but no clear evidence showed that knowledge of allocation materially influenced reporting. | **Some concerns.** No prespecified analysis plan was available; selection among multiple eligible measurements or analyses could not be excluded. | **Some concerns due to D1-D5.** |
| Zhou 2016 | ① | **Some concerns.** An SPSS-generated random sequence and sealed envelopes were used, but envelope safeguards and independent management were unclear. | **Low risk.** The assigned interventions and rescue-treatment criteria were prespecified, with no important deviations identified. | **Low risk.** Outcome data were available for all randomized participants. | **Some concerns.** CCS was participant-reported by unblinded participants, but no clear evidence showed that knowledge of allocation materially influenced reporting. | **Some concerns.** No prespecified analysis plan was available; selection among multiple eligible measurements or analyses could not be excluded. | **Some concerns due to D1, D4 and D5.** |
| Zhu 2019 | ② | **Some concerns.** Randomization was reported, but sequence generation and allocation concealment were not described. | **High risk.** The analysis was restricted to participants who developed constipation after randomization and did not estimate the effect of assignment to intervention. | **High risk.** BSFS data were available only for participants developing constipation after randomization and differed substantially between groups. | **Some concerns.** BSFS was participant-reported by unblinded participants, but no clear evidence showed that knowledge of allocation materially influenced reporting. | **Some concerns.** No prespecified analysis plan was available; selection among multiple eligible measurements or analyses could not be excluded. | **High risk due to D2 and D3.** |
| Zhuo 2017 | ① | **Some concerns.** Randomization was reported, but sequence generation and allocation concealment were insufficiently described. | **Low risk.** Both groups received the assigned interventions, with no important deviations or imbalances in co-interventions identified. | **Low risk.** Outcome data were available for all randomized participants. | **Some concerns.** CCS was participant-reported by unblinded participants, but no clear evidence showed that knowledge of allocation materially influenced reporting. | **Some concerns.** No prespecified analysis plan was available; selection between week-4 and week-8 measurements or among analyses could not be excluded. | **Some concerns due to D1, D4 and D5.** |

Risk of bias was assessed for the end-of-treatment result(s) contributing to the review. Outcomes within the same study were grouped when the domain-level judgments were identical. The effect of interest was assignment to the intervention (the intention-to-treat effect).

Abbreviations: ①, Cleveland Constipation Scale (CCS); ②, Bristol Stool Form Scale (BSFS); ③, Patient Assessment of Constipation–Symptoms (PAC-SYM); ④, spontaneous bowel movements per week (weekly SBMs); ⑤, Bowel Function Index (BFI); ⑥, BFI: ease of defecation; ⑦, BFI: feeling of incomplete bowel evacuation; ⑧, BFI: personal judgment of constipation; ⑨, Patient Assessment of Constipation Quality of Life questionnaire (PAC-QOL); ⑩, PAC-QOL: physical discomfort; ⑪, PAC-QOL: psychosocial discomfort; ⑫, PAC-QOL: worries and concerns; ⑬, PAC-QOL: satisfaction; D1, bias arising from the randomization process; D2, bias due to deviations from the intended interventions; D3, bias due to missing outcome data; D4, bias in measurement of the outcome; D5, bias in selection of the reported result; RoB 2, revised Cochrane Risk of Bias tool for randomized trials.

**Supplement 10. Assessment of transitivity and potential effect modifiers across treatment comparisons**

| **Direct comparison** | **No. of RCTs** | **Cancer type/stage (T/C)** | **Opioid type/dose (T/C)** | **Baseline constipation severity (T/C)** | **Laxative type / concomitant treatment** | **Acupuncture technique** | **Acupoint selection** | **Treatment frequency/duration** | **Transitivity concern** |
| --- | --- | --- | --- | --- | --- | --- | --- | --- | --- |
| EA vs SA | 1 RCT: Wang 2023 | Various cancer types, including lung, liver, breast, pancreatic, cervical, nasopharyngeal, colorectal, gastric, other cancers; metastasis, 42.0% vs 54.0%; exact cancer stage NR. | Oxycodone: 50.0% vs 78.0%; morphine: 20.0% vs 12.0%; other opioids: 30.0% vs 10.0%; mean oral morphine-equivalent dose, 84.2 ± 83.8 vs 73.5 ± 50.9 mg/day. | Baseline PAC-SYM, 2.30 ± 0.80 vs 2.30 ± 0.70 | No routine laxative treatment; bisacodyl suppositories, 5–10 mg (maximum 20 mg/day), and/or a 110-mL glycerol enema were permitted as rescue treatment after ≥72 h without a bowel movement. | EA | Tianshu (ST25), Fujie (SP14), Shangjuxu (ST37) | Three times weekly for 8 weeks; retention time, 30 min. | Some concerns |
| AP vs BC | 2 RCTs:  Yildirim 2022;  Xu 2015 | Yildirim 2022: Various cancer types, including lung, genitourinary, breast, bone, hematological, soft tissue sarcoma; exact cancer stage NR.  Xu 2015: Lung cancer; exact cancer stage NR. | Yildirim 2022: Morphine: 46.38 ± 22.16 vs 42.60 ± 24.42 mg/day; transdermal fentanyl: 27.34 ± 14.73 vs 21.33 ± 15.26 μg/24 h; tramadol: 217.27 ± 101.75 vs 214.27 ± 106.18 mg/day.  Xu 2015: Morphine sulfate sustained-release tablets every 12 h; exact dose NR. | NR | No routine laxative treatment; Yildirim 2022: Both groups maintained their usual diet and fluid intake.  Xu 2015: Both groups received dietary, fluid-intake, and bowel-habit guidance. Glycerin enema or phenolphthalein tablets was permitted as rescue treatment after >72 h without bowel movement. | AP | Yildirim 2022: Tianshu (ST25), Zhongwan (CV12), Guanyuan (CV4).  Xu 2015: Zhigou (TE6), Hegu (LI4), Tianshu (ST25), Daheng (SP15), Zhongwan (CV12), Zusanli (ST36), Sanyinjiao (SP6). | Yildirim 2022: Once daily for 4 weeks; retention time, 8 min;  Xu 2015: Twice daily for 2 weeks; 1–2 s per stroke per acupoint, 50 strokes per session. | Major concerns |
| TEAS+OSM vs SA+OSM | 1 RCT: Cai 2019 | Various advanced malignant tumors, including lung, pancreatic, gastric, breast, cervical/ovarian, colon, hepatic, prostate, melanoma, lymphoma, mesothelioma, soft-tissue sarcoma, metastatic cancer of unknown primary; exact cancer stage NR. | Oxycodone hydrochloride prolonged-release tablets plus immediate-release morphine tablets or morphine injections; exact doses NR. | Baseline BFI, 75.90 ± 22.70 vs 63.50 ± 16.80. | Lactulose, 15 mL twice daily. | TEAS | Main acupoints: Tianshu (ST25), Guanyuan (CV4), Qihai (CV6), Zusanli (ST36), Shangjuxu (ST37), additional points: Ganshu (BL18), Shenshu (BL23), Sanyinjiao (SP6) | Once daily for 2 weeks; retention time, 30 min. | Some concerns |
| TEAS vs OSM | 1 RCT: Zhu 2018 | NR | Oxycodone hydrochloride prolonged-release tablets: ≥80 mg/day; baseline dose, 96.02 ± 16.97 vs 94.00 ± 13.78 mg/day. | Baseline CCS, 14.16 ± 2.03 vs 13.93 ± 1.83. | Lactulose, 10 mL three times daily. | TEAS | Tianshu (ST25), Zhongwan (CV12) | Once daily for 2 weeks; retention time, 30 min. | Some concerns |
| AP+OSM vs OSM | 1 RCT: Chen 2021 | Cancer type NR; Tumor–Node–Metastasis stage II, 8.0% vs 6.0%; stage III, 6.0% vs 8.0%; stage IV, 86.0% vs 86.0%. | Oxycodone hydrochloride prolonged-release tablets: 20–60 mg/day, 66.0% vs 62.0%; 61–100 mg/day, 28.0% vs 30.0%; >100 mg/day, 6.0% vs 8.0%. | Baseline BFI, 68.30 ± 12.10 vs 68.20 ± 12.20. | Lactulose, 15 mL twice daily | AP | Shenque (CV8), Zhongwan (CV12), Tianshu (ST25) | Once daily for 2 weeks; retention time NR. | Some concerns |
| APA vs OSM | 9 RCTs:  Deng 2018;  He 2024;  Liang 2017;  Lin 2021;  Qi 2021;  Tang 2019;  Wang 2017;  Xu 2021b;  Yin 2018 | Deng 2018, Liang 2017, Lin 2021, Qi 2021, Tang 2019, Wang 2017, Xu 2021b, Yin 2018: Various cancer types, mainly including lung, gastrointestinal, hepatobiliary, breast, gynecological, hematological, and other cancers; cancer stage NR.  He 2024: Advanced hepatocellular carcinoma, Barcelona Clinic Liver Cancer advanced or terminal stage. | Deng 2018: Oxycodone hydrochloride prolonged-release tablets: 70.44 ± 42.32 vs 69.11 ± 44.40 mg/day.  Lin 2021: Opioid type NR; <30 mg/day, 10/37 vs 10/36; 30–60 mg/day, 15/37 vs 15/36; >60 mg/day, 12/37 vs 11/36.  Qi 2021: Morphine sulfate sustained-release tablets: 42.4% vs 37.5%; Oxycodone hydrochloride prolonged-release tablets: 36.4% vs 31.3%; Bucinnazine hydrochloride tablets: 18.2% vs 21.9%; Fentanyl transdermal patches: 3.0% vs 9.4%; exact doses NR.  Tang 2019: Oxycodone hydrochloride prolonged-release tablets: every 12 h; exact dose NR.  Wang 2017: Morphine sulfate sustained-release tablets or Oxycodone hydrochloride prolonged-release tablets; exact doses NR.  He 2024, Liang 2017, Xu 2021b, and Yin 2018: NR. | Baseline CCS:  Deng 2018, 16.84 ± 4.58 vs 16.47 ± 4.26;  Lin 2021, 14.14 ± 2.86 vs 14.75 ± 3.09;  Qi 2021, 16.48 ± 3.11 vs 16.78 ± 2.70;  Xu 2021b, 12.62 ± 1.42 vs 12.69 ± 1.49;  Yin 2018, 22.4 ± 4.72 vs 19.5 ± 3.24.  Baseline PAC-SYM: He 2024, 2.49 ± 0.74 vs 2.57 ± 0.72.  Baseline BFI: Liang 2017, exact baseline values NR.  Baseline weekly SBMs: Wang 2017, 8.98 ± 1.68 vs 8.86 ± 1.79.  Tang 2019: NR. | Liang 2017, Qi 2021: Lactulose, 30 mL once daily.  Lin 2021, Yin 2018, and Wang 2017: Lactulose, 15 mL twice daily.  Tang 2019, Xu 2021b: Lactulose, 15 mL three times daily; Tang 2019 additionally permitted a saline enema as rescue treatment after >72 h without a bowel movement.  Deng 2018: Lactulose, 20 mL twice daily; a saline enema was permitted as rescue treatment after >72 h without a bowel movement.  He 2024: Lactulose, initial dose 30 mL/day and maintenance dose 10–25 mL/day. | APA | Deng 2018, He 2024, Liang 2017, Tang 2019, Wang 2017, Xu 2021b, Yin 2018: Shenque (CV8);  Lin 2021: Shenque (CV8), Zhongwan (CV12), Guanyuan (CV4);  Qi 2021: Tianshu (ST25), Guilai (ST29), Dachangshu (BL25), Zhigou (TE6), Shangjuxu (ST37) as main points, with additional syndrome-based points. | Deng 2018 used every 2 days for 2 weeks, retention time, 48 h;  He 2024 used once daily for 4 weeks, retention time, 4 h;  Liang 2017 used once daily for 1 week, retention time, 24 h;  Lin 2021 used once daily for 2 weeks, retention time, 4 h;  Qi 2021, Tang 2019, Xu 2021b used once daily for 1 week, retention time, 6 h;  Wang 2017 used once daily for 2 weeks, retention time, 4–6 h;  Yin 2018 used once daily for 1 week, retention time, 12 h. | Major concerns |
| EA vs OSM | 2 RCTs:  Du 2012;  Li 2020 | Du 2012: NR.  Li 2020: Various cancer types, including lung, pancreatic, breast, ovarian, prostate cancers. exact cancer stage NR. | Du 2012: Opioid type NR; 50.00 ± 33.20 vs 43.20 ± 17.20 mg/day.  Li 2020: Oxycodone hydrochloride prolonged-release tablets: 45.00 ± 23.45 vs 46.50 ± 15.99 mg/day. | Du 2012: Baseline CCS, 11.20 ± 2.58 vs 11.08 ± 1.35.  Li 2020: Baseline weekly SBMs, 1.14 ± 0.35 vs 1.35 ± 0.59. | Du 2012: Lactulose, 10 mL twice daily.  Li 2020: Lactulose, 30 mL once daily. | EA | Du 2012: Main acupoint: Tianshu (ST25), additional points based on syndrome differentiation: Qihai (CV6), Zusanli (ST36), Guanyuan (CV4), Taixi (KI3), Hegu (LI4), Sanyinjiao (SP6).  Li 2020: Tianshu (ST25), Shangjuxu (ST37), Zhigou (TE6), Zusanli (ST36) | Du 2012: Five times weekly for 2 weeks; retention time, 30 min.  Li 2020: Once daily for 2 weeks; retention time, 20 min. | Major concerns. |
| APA vs BC | 3 RCTs:  Li 2024;  Ma 2023;  Xia 2024 | Li 2024: Various cancer types, including Gastric, breast, lung, liver, esophageal cancers; cancer stage NR.  Ma 2023: NR.  Xia 2024: Advanced lung cancer, including non-small cell lung cancer, 29 vs 28 patients, and small-cell lung cancer, 6 vs 7 patients; exact cancer stage NR. | NR. | Li 2024: Baseline BSFS, 2.59 ± 0.55 vs 2.69 ± 0.02.  Ma 2023: NR.  Xia 2024: Baseline PAC-SYM, 3.69 ± 0.39 vs 3.72 ± 0.40. | No routine laxative treatment.  Li 2024: Both groups received dietary guidance, abdominal massage, bowel-habit training, emotional support, health education, and aerobic exercise.  Ma 2023: Both groups received dietary and fluid guidance, abdominal massage, bowel-habit training, and psychological support; use of a glycerin enema or oral lactulose was permitted as needed, and a cleansing enema was to be administered once after >72 h without a bowel movement.  Xia 2024: Both groups received health education, fluid intake of approximately 2,000–3,000 mL/day, dietary guidance, increased physical activity, and emotional support. | APA | Li 2024: Yongquan (KI1);  Ma 2023 and Xia 2024: Shenque (CV8); | Li 2024: Once daily for 2 months; retention time 6 h.  Ma 2023: Once daily; retention time NR.  Xia 2024: Once daily for 1 week; retention time 6 h. | Major concerns |
| APA vs STI | 1 RCT: Lin 2014 | NR | NR | Baseline CCS, 12.86 ± 1.20 vs 12.78 ± 1.50. | Phenolphthalein tablets, 200 mg three times daily | APA | Tianshu (ST25), Zusanli (ST36), Guanyuan (CV4), Sanyinjiao (SP6), Zhigou (TE6), Neiting (ST44), Qihai (CV6) | Once daily for 2 weeks; retention time, 4–6 h. | Major concerns |
| MOX vs OSM | 1 RCT: Liu 2022 | Various cancer types, including esophageal, gastric, liver, pancreatic cancers; liver cancer, 11 vs 9; gastric cancer, 12 vs 12; pancreatic cancer, 9 vs 10; esophageal cancer, 1 vs 2; exact cancer stage NR. | NR | NR; | Lactulose, 10 mL three times daily. | MOX | Qihai (CV6), Guanyuan (CV4), Zhongwan (CV12), Shenque (CV8) | Once daily for 2 weeks; retention time, 45 min. | Major concerns |
| PN vs OSM | 1 RCT: Ma 2019 | NR | NR | Baseline CCS, 12.3 ± 3.1 vs 12.4 ± 3.0. | Lactulose, 15 mL twice daily; a 110 mL glycerin enema was permitted as rescue treatment after >96 h without bowel movement. | PN | Zusanli (ST36), Hegu (LI4), Taichong (LR3), Zhigou (TE6), Shangjuxu (ST37) | Every 3 days for 3 weeks; 1 min per session, once every 2–3 h. | Major concerns |
| APA+OSM vs OSM | 4 RCTs:  Mao 2017;  Ouyang 2023;  Yang 2019;  Zhu 2019 | Mao 2017: Various cancer types, including Lung, liver, breast, pancreatic, nasopharyngeal cancers; exact cancer stage NR.  Ouyang 2023: Various advanced cancer types, including lung, gastrointestinal, hepatobiliary-pancreatic, gynecological/urinary, esophageal, breast, other cancers; exact cancer stage NR.  Yang 2019: Various cancer types, including digestive-system, respiratory-system, gynecological, hematological/lymphatic, other cancers; cancer stage NR.  Zhu 2019: Various cancer types, lung, breast, pancreatic, cervical, colorectal cancers; exact cancer stage NR. | Mao 2017: NR.  Ouyang 2023: Oxycodone hydrochloride prolonged-release tablets: initial dose 10–20 mg every 12 h, adjusted according to pain control; <30 mg/12 h, 53/68 vs 46/61; 30–80 mg/12 h, 13/68 vs 12/61; >80 mg/12 h, 2/68 vs 3/61.  Yang 2019: Oxycodone hydrochloride prolonged-release tablets: 40–80 mg/day.  Zhu 2019: Oxycodone hydrochloride prolonged-release tablets: initial dose, 36.6 ± 17.8 vs 35.4 ± 15.4 mg/day. | Baseline CCS:  Mao 2017, 18.41 ± 3.77 vs 18.61 ± 4.03.  Ouyang 2023, 20.32 ± 3.16 vs 20.30 ± 3.42.  Yang 2019: Baseline BFI, 68.56 ± 10.66 vs 65.83 ± 4.61.  Zhu 2019: NR. | **Mao 2017, Ouyang 2023, and Zhu 2019:** Lactulose, 15 mL twice daily.  **Yang 2019:** Lactulose, 15 mL three times daily. | APA | Shenque (CV8) | Mao 2017: Once daily, 12 h per session; four applications per course, with a 1-day interval between two courses.  Ouyang 2023: One application every 2 days for 14 days; retention time, 48 h.  Yang 2019: Once daily for 7 days; retention time, 8 h.  Zhu 2019: Once daily for 10 days; retention time, 8 h. | Some concerns |
| AA+OSM vs OSM | 2 RCTs:  Qu 2016;  Yang 2014 | Qu 2016: NR.  Yang 2014: Various cancer types, including gastric, lung, esophageal, prostate, breast cancers; exact cancer stage NR. | Qu 2016: NR.  Yang 2014: Morphine sulfate sustained-release tablets, oxycodone hydrochloride prolonged-release tablets, and fentanyl transdermal patches; exact doses NR. | Qu 2016: NR.  Yang 2014: Baseline BFI, 91.00 ± 5.23 vs 90.73 ± 5.45 | lactulose, 15 mL three times daily. | AA | Qu 2016: Triple Burner (TF4), Spleen (CO13), Stomach (CO4), Liver (CO12), Lung (CO14), Large Intestine (CO7);  Yang 2014: Main auricular points: Subcortex (AT4), Brainstem, Abdomen (TF2), Large Intestine (CO7), additional auricular points: Liver (CO12), Spleen (CO13), Triple Burner (TF4), Lung (CO14), Kidney (CO10). | Qu 2016: opposite side every 3 days; Continuous pressure for 20 s, 4–6 presses per point, 5–6 times/day;  Yang 2014: Once daily for 2 weeks; pressed 4 times/day, 2 min per acupoint. | Major concerns. |
| MA vs OSM | 1 RCT: Tian 2020 | Various cancer types, including nasopharyngeal, gastric, cholangiocarcinoma, lung, liver, ovarian, bladder, breast, esophageal, pancreatic, prostate cancers; exact cancer stage NR. | NR | NR | Lactulose, 15 mL twice daily; use of a glycerin enema or saline enema was permitted as rescue treatment when patients remained without bowel movement. | MA | Tianshu (ST25), Shangjuxu (ST37), Zusanli (ST36), Hegu (LI4), Dachangshu (BL25), Pishu (BL20), Zhigou (TE6) | Five times weekly for 2 weeks; retention time, 30 min. | Major concerns |
| MOX+OSM vs OSM | 1 RCT: Xu 2021a | Various non-digestive cancer types, mainly including lung, breast, nasopharyngeal, and prostate cancers; exact cancer stage NR. | specific opioid type NR; Mean daily morphine dose, 126.4 vs 132.0 mg/day. | Baseline BSFS, 1.68 ± 0.36 vs 1.74 ± 0.33; Baseline weekly SBMs, 2.92 ± 0.82 vs 2.88 ± 0.64 | Lactulose, 30 mL daily. | MOX | Heat-sensitive acupoints | Five times weekly for 3 weeks; retention time, 30–60 min. | Some concerns |
| PN+OSM vs OSM | 1 RCT: Zhang 2023 | Various cancer types, including lung, gastric, liver, esophageal, and ovarian cancers; exact cancer stage NR. | Morphine sulfate sustained-release tablets, oxycodone hydrochloride prolonged-release tablets, and fentanyl transdermal patches; exact doses NR. | Baseline CCS, 20.78 ± 3.21 vs 20.89 ± 2.84;  Baseline BSFS, 2.15 ± 0.54 vs 2.09 ± 0.41. | lactulose, 15 mL three times daily. | PN | Tianshu (ST25), Dachangshu (BL25), Shangjuxu (ST37), Zhigou (TE6), Zusanli (ST36) | Once daily for 2 weeks; retention time, 24 h. | Some concerns |
| APA vs SA | 1 RCT: Zhao 2021 | Various cancer types, including lung, breast, liver, and pancreatic cancers; cancer exact stage NR. | oxycodone hydrochloride prolonged-release tablets; exact doses NR. | NR | No routine laxative treatment.  Both groups received dietary guidance, fluid intake of ≥1,500 mL/day, appropriate exercise, abdominal massage, and bowel-habit training; A 500 mL saline cleansing enema was to be administered once after >72 h without a bowel movement. | APA | Shenque (CV8) | Once daily for 10 days; retention time, 24 h. | Major concerns |
| ACE+OSM vs OSM | 1 RCT: Zheng 2022 | Various cancer types, including lung, liver, esophageal, cervical, breast, ovarian, nasopharyngeal, and thymic cancers; stage III, 20/33 vs 18/33; stage IV, 13/33 vs 15/33. | NR | Baseline BSFS, 1.39 ± 0.23 vs 1.45 ± 0.18. | Lactulose, 15 mL twice daily; a glycerin enema was permitted as rescue treatment after >5 days without a bowel movement and with evident symptoms. | ACE | Tianshu (ST25), Dachangshu (BL25), Zusanli (ST36) | Once weekly for 1 week; retention time NR. | Some concerns |
| MOX vs STI | 1 RCT: Zhou 2016 | NR | Oxycodone hydrochloride prolonged-release tablets: 16 vs 19; fentanyl transdermal patches 14 vs 21; exact doses NR. | Baseline CCS, 14.14 ± 2.14 vs 13.71 ± 2.30. | Phenolphthalein tablets, 100–200 mg daily, with the dose and dosing interval adjusted according to constipation improvement; glycerin or saline enema was permitted as rescue treatment for severe constipation. | MOX | Tianshu (ST25), Zhongwan (CV12), Shenque (CV8), Shangjuxu (ST37), Zusanli (ST36), bilateral Zhigou (TE6), Guanyuan (CV4). | Once daily for 10 days; retention time, 2–30 min per acupoint. | Major concerns |
| MA+OSM vs OSM | 1 RCT: Zhuo 2017 | Various cancer types, including lung, liver, prostate, breast, and cholangiocarcinoma; stage II, 3/60; stage III, 8/60; stage IV, 49/60; group-specific distributions NR. | NR | Baseline CCS, 16.17 ± 3.83 vs 16.23 ± 3.87. | Lactulose, 15 mL three times daily | MA | Baihui (GV20), Guanyuan (CV4), Qihai (CV6), Tianshu (ST25), Shangjuxu (ST37), Zusanli (ST36), Hegu (LI4), Taichong (LR3), Zhigou (TE6). | Five times weekly for 8 weeks; retention time, 30 min. | Some concerns |

**Abbreviations:** RCT, randomized controlled trial; NR, not reported; OSM, osmotic laxatives; STI, stimulant laxatives; BC, blank control; SA, sham acupuncture; EA, electroacupuncture; APA, acupoint application; AP, acupressure; AA, auricular acupuncture; ACE, acupoint catgut embedding; TEAS, transcutaneous electrical acupoint stimulation; MOX, moxibustion; PN, press needle; MA, manual acupuncture. T/C indicates treatment/control group. CCS, Cleveland Constipation Scale; BSFS, Bristol Stool Form Scale; PAC-SYM, Patient Assessment of Constipation–Symptoms; weekly SBMs, spontaneous bowel movements per week; BFI, Bowel Function Index.

**Note:** Transitivity concerns were judged according to the availability and balance of key potential effect modifiers, including cancer type/stage, opioid type/dose, baseline constipation severity, laxative type/concomitant treatment, acupuncture technique, acupoint selection, treatment frequency, and treatment duration. Ratings were assigned at the direct-comparison level based primarily on the completeness of reporting and the comparability of potential effect-modifier distributions across different treatment comparisons in the network; within-study balance between treatment and control groups was considered as supplementary information. A rating of “No major concern” indicated that the main reported effect modifiers were broadly comparable and that no important imbalance was identified. A rating of “Some concerns” indicated incomplete reporting or moderate variation in one or more potential effect modifiers, without clear evidence of a substantial imbalance. A rating of “Major concerns” indicated substantial missing information and/or marked heterogeneity or imbalance in multiple potential effect modifiers, limiting the assessment of transitivity. Single-study comparisons provided limited information for assessing transitivity but were not automatically classified as having major concerns.

**Supplement 11. Handling of the zero standard deviation value and sensitivity analysis for BSFS**

| **Analysis** | **Outcome** | **Comparison** | **Arm with zero SD** | **Handling of the zero SD in**  **Qi 2021** | **No. of included RCTs** | **Intervention/control (n)** | **Pairwise result**  **MD (95% CI)** | ***I*²** | ***P*** | **NMA result**  **MD (95% CrI)** | **SUCRA for APA** | **Treatment with the highest ranking probability** |
| --- | --- | --- | --- | --- | --- | --- | --- | --- | --- | --- | --- | --- |
| **Sensitivity analysis** | BSFS | APA vs OSM | APA | Included using the imputed SD of 1.31 | 3 | 100/98 | -0.17 (-0.52, 0.18) | 28% | 0.3461 | -0.05 (-1.36, 1.28) | 0.65 | PN+OSM, SUCRA = 0.72 |

**Abbreviations:** BSFS, Bristol Stool Form Scale; APA, acupoint application; OSM, osmotic laxatives; BC, blank control.

**Note:** The imputed SD of 1.31 was calculated as the pooled within-group SD across all non-zero post-treatment BSFS arms from the other included studies, with arm-specific variances weighted by their degrees of freedom:$\text{ }\text{S}\text{D}_{\text{pooled}}\text{=}\sqrt{\sum\left( \text{n}_{\text{i}}\text{−1} \right)\text{S}\text{D}_{\text{i}}^{\text{2}}\text{/}\sum\left( \text{n}_{\text{i}}\text{−1} \right)}\text{=1.3121,}\text{ }\text{roundedto1.31.}$
